# Prominin 2 promotes the progression of breast cancer by inhibiting ferroptosis *via* MAPK signaling

**DOI:** 10.1515/jtim-2026-0061

**Published:** 2026-06-22

**Authors:** Mingchuan Zhao, Juan Jin, Zhonghua Tao, Yannan Zhao, Biyun Wang, Xichun Hu, Hongxia Wang

**Affiliations:** Department of Oncology, Shanghai Medical College, Fudan University, Shanghai, China; Department of Medical Oncology, Fudan University Shanghai Cancer Center, Shanghai, China

**Keywords:** prominin 2, ferroptosis, breast cancer, MAPK

## Abstract

**Background and Objectives:**

Ferroptosis is an iron-dependent form of programmed cell death, and its dysregulation has been implicated in the progression of breast cancer (BC). Prominin 2 (PROM2) has been reported as a negative regulator of ferroptosis. This study aims to investigate the role of PROM2 in BC progression and its underlying molecular mechanism.

**Methods:**

Bioinformatics analysis was performed to evaluate the expression and prognostic relevance of PROM2 in BC cohort from public database. The expression levels of PROM2 were assessed in BC tissues and cell lines. Knockdown of PROM2 was achieved using lentiviral-mediated shRNA, and the effect of PROM2 silencing in the tumor growth was evaluated in a xenograft model of nude mice.

**Results:**

The results demonstrated that PROM2 was overexpressed in BC tissues and its heightened expression was associated with poor prognosis. Knockdown of PROM2 in BC cell lines suppressed cell proliferation, migration, and invasion, while promoting ferroptosis, as evidenced by increased levels of reactive oxygen species (ROS), lipid peroxidation, and malondialdehyde, as well as decreased levels of glutathione and GPX4 expression. Furthermore, PROM2 was found to activate the mitogen-activated protein kinase (MAPK) signaling pathway, and inhibition of this pathway abrogated the pro-tumorigenic effects of PROM2 overexpression.

**Conclusions:**

Collectively, our findings pinpoint a crucial role of PROM2 in BC progression by inhibiting ferroptosis and activating the MAPK signaling pathway. Targeting PROM2 might represent a potential therapeutic strategy for BC treatment.

## Introduction

Breast cancer (BC) is a highly heterogeneous disease with a complex and multifaceted etiology. Its development is influenced by a diverse array of factors, including genetic predispositions, hormonal imbalances, and lifestyle choices.^[1, 2, 3, 4, 5]^ Despite the relatively favorable 5-year survival rates exceeding 60% for patients diagnosed with early-stage (I, II, or III) BC, a substantial proportion, approximately 20%–30%, will experience disease recurrence and metastasis.^[6]^ In United States, approximately 299,200 new BC cases are diagnosed with varying 5-year survival rates of 99% for localized, 86% for regional, and 33% for metastatic disease, resulting in an estimated 43,700 deaths in 2024.^[7]^ Current cancer treatment modalities encompass a personalized approach combining traditional methods (surgery, chemotherapy, and radiation) with advanced targeted therapies such as immunotherapy (including CAR-T cells and immune checkpoint inhibitors).^[8]^ However, the propensity for recurrence and dissemination poses a formidable challenge in the management of BC.^[6]^ Identifying novel therapeutic targets, prognostic biomarkers, and developing innovative and effective treatment strategies remain at the forefront of ongoing BC research endeavors.

Ferroptosis, a recently defined form of programmed cell death, is characterized by iron-dependent oxidative stress and lipid peroxidation (LPO).^[9,10]^ This distinct mode of cell death, marked by the accumulation of intracellular reactive oxygen species (ROS), exhibits morphological features that distinguish it from apoptosis and autophagy.^[11]^ The discovery of small molecules which are capable of triggering iron-dependent non-apoptotic cell death (such as RSL3 and sulforaphane) has positioned the induction of ferroptosis as a potential therapeutic strategy, particularly for patients unresponsive to conventional treatments.^[12,13]^ Accumulating evidence has underscored the link between ferroptosis and BC progression. Notably, the novel adjuvant neratinib has been demonstrated to promote ferroptosis and inhibit brain metastasis in a BC metastasis model.^[14]^ Moreover, certain triple-negative BC cells exhibit heightened sensitivity to ferroptosis induction.^[15,16]^ Several genes, including (Acyl-CoA Synthetase Long-Chain Family Member 4) ACSL4, which modulates cellular sensitivity to ferroptosis by altering lipid composition, have been identified as crucial regulators of BC development.^[17]^ Targeted therapies directed at aberrant mitogen-activated protein kinase (MAPK) signaling pathways, which are frequently dysregulated in aggressive cancers, have demonstrated therapeutic efficacy.^[18]^ Targeting ferroptosis-related regulators, including iron metabolism proteins, LPO mediators, and antioxidant systems, has been proposed as a therapeutic strategy for highly aggressive BC through modulation of cell death pathways.^[15,16]^

Prominin 2 (PROM2) is a protein involved in the formation of multivesicular bodies and exosome secretion. Brown *et al*. demonstrated that PROM2 promotes ferroptosis resistance in cancer cells by stimulating the export of iron *via* multivesicular bodies (MVBs), thereby reducing intracellular iron levels and preventing iron-dependent LPO.^[19]^ Notably, PROM1 and PROM2 (prominin genes) seem to differentially modulate clinical prognosis across various cancer types.^[20]^ Specifically, high PROM1 expression was associated with better prognosis, while high PROM2 expression correlated with poorer prognosis in certain cancers like BC.^[20]^ Furthermore, suppressing the transcription of PROM2 sensitizes cancer cells to ferroptosis induction, suggesting that targeting PROM2 could be a potential therapeutic strategy to circumvent ferroptosis resistance.^[21,22]^ The objective of this study is to comprehensively investigate the expression pattern and prognostic relevance of PROM2 in BC. We delineated the functional effects of PROM2 on BC cell proliferation, migration, invasion, and ferroptosis sensitivity through *in vitro* experiments involving PROM2 knockdown. Mechanistic insights into the potential involvement of the MAPK signaling pathway in PROM2-mediated ferroptosis resistance was explored. The findings from this study may unravel a novel regulatory mechanism underlying BC progression and provide a rationale for targeting PROM2 as a therapeutic strategy against this malignancy.

## Materials and methods

### Bioinformatic analyses to elucidate the relationship between PROM2 and breast cancer progression

Leveraging the comprehensive gene expression data from The Cancer Genome Atlas (TCGA-BC) database (https://portal.gdc.cancer.gov/projects/TCGA-BRCA) ^[23]^ and the Genotype-Tissue Expression (GTEx) database (https://gtexportal.org/home/) for normal breast tissue samples, we analyzed PROM2 expression levels across 1073 breast cancer tumor samples and 293 normal breast tissue samples (112 from TCGA-BC and 180 from GTEx). All datasets were subjected to unified batch correction and normalization using to account for potential batch effects. Log2-transformed FPKM values were used for subsequent analyses. To corroborate these findings, PROM2 expressions in BC tissues were further examined using expression profile datasets from Gene Expression Omnibus (GEO) (https://www.ncbi.nlm.nih.gov/geo/) with RMA normalization applied. Univariate and multivariate Cox regression analyses were performed using R software (version 4.0.0, survival package version 3.2-7) with hazard ratios calculated at 95% confidence intervals to evaluate the association between PROM2 expression and BC progression. Additionally, CIBERSORTx algorithm (https://cibersortx.stanford.edu/) was employed with default parameters (1000 permutations, quantile normalization disabled) to quantify the infiltration levels of 22 immune cell populations, and results were further analyzed using the TIMER database (https://cistrome.shinyapps.io/timer/) based on PROM2 expression levels. The ESTIMATE software package (R package, version 1.0.13) was utilized to compute stromal and immune scores using the estimateScore function with default parameters, which serve as surrogate measures of tumor microenvironment composition.^[24]^ Finally, Spearman’s correlation analyses were conducted using R software (cor. test function with method = “spearman”) to assess the relationship between PROM2 gene expression and stromal and immune scores, providing insights into the potential link between PROM2 and tumor-immune interactions in BC.

### Clinical tissue collection

To elucidate the potential role of PROM2 in BC development and progression, our study utilized a clinically relevant cohort of 100 BC patients who underwent surgical resection at our hospital between September 2019 and September 2023. From each patient, both tumor tissue and adjacent non-tumorous breast tissue specimens were collected. The cohort comprised exclusively female patients ranging in age from 45 to 68 years, with a mean age of 53.1 years. Patients who received neoadjuvant therapy or presented with metastatic disease were excluded from this study. Only treatment-naive BC patients undergoing primary surgical resection at our institution during the study period were included. All procedures adhered to ethical guidelines established by the Institutional Review Board and were conducted in accordance with the principles outlined in the Declaration of Helsinki.^[25]^ Informed consent was obtained from each participant prior to enrollment, with patients providing written consent for the use of their clinical samples in research investigations. Stringent measures were taken to ensure the privacy and confidentiality of patient information throughout the study.

### RT-PCR detection of PROM2 expression

Total RNA was isolated from 10 mg tissue or 1 × 10^6^ cells using Trizol reagent from Thermo Fisher Scientific (Waltham, MA, USA). The RNA concentration and purity were meticulously assessed and quantified using a UV spectrophotometer. Subsequently, the RNA was reverse-transcribed into complementary DNA (cDNA) using the Novizen Reverse Transcription Kit (Novizen Inc., Seoul, South Korea), following the manufacturer’s instructions. The synthesized cDNA was diluted 20-fold and utilized as a template for quantitative real-time polymerase chain reaction (qPCR) analysis. The qPCR reactions were performed using the Premix Ex TaqTM PCR kit from Novizen (Novizen Inc., Seoul, South Korea), with the following cycling conditions: an initial denaturation step at 95 °C for 30 s, followed by 42 cycles of denaturation at 95 °C for 5 s and combined annealing/ extension at 60 °C for 30 s. The amplification curves were acquired on a QuantStudio 5 Real-Time PCR System (Applied Biosystems, Foster City, CA, USA) to quantify the relative expression levels of the target mRNA transcripts in the samples. The results were analyzed by the 2^-ΔΔCt^ method. Forward primer sequence of *PROM2* was 5’-GGGCCACAGACTGCAAGTT-3’, and the reverse primer sequence was 5’-AGCTCATTCA GTAGGGCCTTTA-3’. Forward primer sequence of *GAPDH* was 5’-CGACCACTTTGTCAAGCT CA-3’, and the reverse primer sequence was 5’-GGTTGAGCACAGGGTACTTTATT-3’. *GAPDH* was used as the internal reference gene. PCR was repeated three times.

### Cell culture

BC cell lines (MDA-MB-231, Hs578T, MDA-MB-453, and MCF7) and human normal mammary epithelial cells MCF-10A (Cell Bank, Shanghai, China) were cultivated in Dulbecco’s Modified Eagle’s Medium (DMEM) supplemented with 10% fetal bovine serum (FBS), 100 kU/L penicillin, and 100 mg/L streptomycin (GIBCO, Gaithersburg, MD, USA). All cell lines were authenticated by short tandem repeat (STR) profiling at Cell Bank (Shanghai Institute of Cell Biology, Chinese Academy of Sciences) and regularly tested for mycoplasma contamination using MycoAlert™ Mycoplasma Detection Kit (Lonza, Basel, Switzerland). The cells were maintained in a humidified incubator at 37 °C with 5% CO_2_ atmosphere. For p38 inhibition, cell were treated with 10 μmol/L SB202190 (HY-10295, MedChemExpress, Shanghai, China).

### Immunohistochemistry

Formalin-fixed, paraffin-embedded tumor tissues and adjacent non-tumorous tissues obtained from BC patients were sectioned at a thickness of 4 μm and mounted onto glass slides. The tissue sections were deparaffinized and rehydrated using standard protocols. To quench endogenous peroxidase activity and block non-specific protein binding, the sections were treated with 3% hydrogen peroxide and goat serum, respectively, and incubated for 20 min. Subsequently, the sections were incubated overnight at 4 °C with a primary antibody against Prominin 2 (ab118492, Abcam, Cambridge, MA, USA) and phospho-p38 antibody (ab47363, Abcam) diluted at 1:250 in blocking buffer. After rinsing and equilibrating to room temperature, the sections were incubated with a horseradish peroxidase-conjugated secondary antibody (ab205718, Abcam, Cambridge, MA, USA) diluted at 1:50 in blocking buffer. The colorimetric signal was developed by applying 3,3’-diaminobenzidine (DAB) chromogen solution, and the reaction was monitored under a microscope. Five randomly selected high-magnification fields of view were scored for the percentage of positive staining. The protein expression levels of PROM2 were quantified using Image J software (National Institutes of Health, Bethesda, MD, USA).

### Western blot assay

MDA-MB-231, Hs578T, MDA-MB-453, MCF-7 and human normal mammary epithelial cells MCF-10A cells were inoculated into 6-well plates at 2 × 10^5^ cells/well. After 24 h of incubation, the cells were lysed using RIPA buffer, and the total protein was collected for quantification using the BCA protein assay kit (Beyotime Biotechnology, Beijing, China). Equal amounts of protein (30 μg) from each sample were loaded onto polyacrylamide gels for sodium dodecyl sulfate-polyacrylamide gel electrophoresis (SDS-PAGE). Subsequently, the separated proteins were transferred onto polyvinylidene difluoride (PVDF) membranes, which were then blocked with 5% non-fat dry milk in Tris-buffered saline containing 0.1% Tween-20 (TBST) for 1 h at room temperature. The membranes were probed overnight at 4 °C with primary antibodies against Prominin 2 (ab74997, Abcam, Cambridge, MA, USA) diluted at 1:1000 and GAPDH (sc-365062, Santa Cruz Biotechnology, Dallas, TX, USA) diluted at 1:4000 as a loading control. After washing, the membranes were incubated with a fluorescent-labeled secondary antibody (ab7090, Abcam, Cambridge, MA, USA) diluted at 1:2000 in TBST. The chemiluminescent signals were captured using ECL Plus reagent (Life Technologies, Carlsbad, CA, USA), and the band intensities were quantified using ImageJ software (National Institutes of Health, Bethesda, MD, USA). The relative expression levels of the target proteins were calculated by normalizing to the GAPDH loading control.

### Knockdown of PROM2 expression in breast cancer cells

Hs578T and MDA-MB-453 BC cell lines were used for subsequent experiments. Each cell line was divided into two groups: a control group and an interference group. To achieve PROM2 gene silencing, lentiviral particles carrying short hairpin RNA (shRNA) sequences targeting PROM2 (obtained from Genscript Inc., Piscataway, NJ, USA) were employed for RNA interference (RNAi) experiments. The cells in the interference group were transduced with the lentiviral particles carrying PROM2-specific shRNA, while the control group received a non-targeting scrambled shRNA sequence. The efficiency of PROM2 knockdown in the interference group was subsequently evaluated by qRT-PCR analysis. The following sequences of shRNA were used in the experiment:

Scramble shRNA: 5’-ATGAGCATACGCACCAATTGC-3’; shPROM#1 5’-GCACCTGGATATCAACCAGTA-3’; shPROM2#2 5’-CCTCCAAATACTTCCGTCCTA-3’; shPROM2#3 5’-GCCTGAAAGTAGACACACAGA-3’.

### CCK8 assay for cell growth measurement

Hs578T and MDA-MB-453 cells transduced with either sh-NC (non-targeting control) or sh-PROM2 lentiviral particles were harvested during the logarithmic growth phase and subjected to trypsin-EDTA dissociation. The cells were seeded into 96-well plates at a density of 1,500 cells per well in a volume of 200 μL, with four replicates per condition. The plates were then incubated in a humidified incubator at 37 °C with 5% CO_2_ for 12, 24, 48, and 72 h. At each time point, 10 μL of the Cell Counting Kit-8 (CCK-8) reagent (Dojindo Molecular Technologies, Kumamoto, Japan) was added to each well, and the plates were further incubated for 2–4 h. The optical density (OD) of the resulting formazan product was measured at a wavelength of 450 nm using a microplate reader to assess cell viability and proliferation.

### Transwell assay

For the cell migration assays, Hs578T and MDA-MB-453 cells were dissociated with trypsin-EDTA, washed, and resuspended in serum-free medium. Approximately 6 × 10^4^ cells in a volume of 250 μL were seeded into the upper chambers of Transwell inserts (24-well format, 8-μm pore size; Corning, Inc., Corning, NY, USA). For invasion assay, the upper chambers of Transwell inserts were coated with Matrigel to mimic extracellular matrix. Complete growth medium supplemented with serum (750 μL) was added to the lower chambers, serving as a chemoattractant. The Transwell plates were then incubated in a humidified incubator at 37 °C with 5% CO_2_ for 48 h to allow for cell migration through the porous membrane. After the incubation period, the non-migrated cells remaining on the upper surface of the membrane were gently removed with a cotton swab. The migrated cells on the lower surface of the membrane were fixed and stained with 0.5% crystal violet solution (Sigma-Aldrich, St. Louis, MO, USA) for 10 min. Excess stain was carefully washed off with phosphate-buffered saline (PBS). The membranes were then visualized and imaged using an inverted microscope. The number of migrated cells was quantified using ImageJ software (National Institutes of Health, Bethesda, MD, USA).

### Ferrous ion detection

Phen Green SK (PGSK) diacetate is a fluorescent heavy metal indicator that reacts with various metal ions, including ferrous iron (Fe^2+^). The intracellular Fe^2+^ levels were determined using a commercially available Fe^2+^ assay kit (Elabscience Biotechnology Co., Ltd., Wuhan, China), based on PGSK diacetate staining. A standard curve was generated using the provided standard samples to quantify the Fe^2+^ levels in the cell samples. Each experimental condition was set up in triplicate wells, and the experiment was independently repeated three times to ensure reproducibility and statistical significance. The fluorescent signal emitted by the PGSK diacetate probe, indicative of Fe^2+^ levels, was measured using a fluorescence microplate reader (SpectraMax iD3, Molecular Devices, San Jose, CA, USA).

### Detection of intracellular reactive oxygen species, glutathione, lipid peroxidation, and malondialdehyde

Intracellular ROS levels were measured using the 2′,7′-dichlorodihydrofluorescein-diacetate (DCFH-DA) assay. Cells were seeded at 5,000 cells/well in a 96-well plate and were incubated with 10 μmol/L DCFH-DA at 37 °C for 30 min. After incubation, the cells were washed three times with PBS and co-stained with Hoechst 33342 solution for 15 min. Following three additional washes with PBS, the cells were analyzed using a laser confocal scanning microscope (excitation: 488 nm, emission: 510–530 nm) to observe the fluorescence intensity, indicative of intracellular ROS levels. All values were normalized to total protein content.

Reduced glutathione (GSH) levels were measured using a GSH colorimetric assay kit (Elabscience, Catalog No.: E-BC-K030-S). This assay is based on the principle that GSH reacts with 2-nitrobenzoic acid (DTNB) to form a yellow-colored compound, which was detected colorimetrically at a wavelength of 420 nm, allowing for the quantitative determination of reduced GSH.

LPO levels were detected according to the manufacturer’s instructions (Elabscience, item number: E-BC-F003) using a fluorescent probe that reacts with lipid free radicals in the LPO pathway. This fluorescent probe emits red fluorescence under normal conditions upon light excitation, and the fluorescence changes from red to green as LPO proceeds. The formation of LPO was detected with high sensitivity by monitoring the increase in green fluorescence intensity.

Malondialdehyde (MDA), a byproduct of LPO, was measured according to the instructions provided by the kit manufacturer (Elabscience, Catalog No. E-BC-K025). MDA reacts with thiobarbituric acid (TBA) under high temperature and acidic conditions to form a reddish-brown compound, trimethyloxazolidine (3,5,5-trimethyloxazolidine-2,4-dione), which exhibits a peak absorption at 532 nm. All the data were normalized againt the protein contents

### Animal experiments

The animal studies were approved by the Institutional Animal Care and Use Committee of the hospital. Eight-week-old female BALB/c nude mice were purchased from Vital River Beijing Viton Lihua Laboratory Animal Science and Technology Co., Ltd. (Beijing, China). To validate the role of PROM2, ten mice were randomly divided into two groups: sh-NC group (inoculated with Hs578T cells transduced with non-targeting control shRNA) and sh-PROM2 group (inoculated with Hs578T cells transduced with PROM2-targeting shRNA). Another experiment was set up with 5 mice in the control group and another five animals in p38 inhibition group, with both groups inoculated with Hs578T cells. Animals in the control group were treated with normal saline, and p38 inhibition was achieved by i.p. injection of 10 mg/kg/d SB202190. Cells with cell viability greater than 98% were collected and subcutaneously inoculated into the mice near the axillary region at a density of 1 × 10^6^ cells in 100 μL of cell suspension per mouse. Once palpable tumor masses were formed, the tumor dimensions (long diameter, L; and short diameter, W) were measured every 4 d using a Vernier caliper to calculate the tumor volume using the formula: (L × W^2^)/2. Twenty-two days after inoculation, the tumor-bearing mice were euthanized, and the tumors were excised and weighed.

The tumor tissues were further sectioned and subjected to immunohistochemical staining to evaluate the expression levels of the antioxidant enzymes SOD1 and Gpx4. Primary antibodies used for immunohistochemistry (IHC) were anti-SOD1 (ab51254, Abcam, Cambridge, MA, USA) and anti-Gpx4 (ab125066, Abcam, Cambridge, MA, USA). PROM2 immunohistochemical staining was evaluated using the IRS score (Immunoreactive score), where both staining intensity and percentage of positive cells were considered. Staining intensity was scored as follows: 0 (no staining), 1 (weak staining, light brown), 2 (moderate staining, medium brown), and 3 (strong staining, dark brown). The percentage of positive cells was evaluated as: 1 (1%–10%), 2 (11%–50%), 3 (51%–80%), and 4 (81%–100%). The final IHC score was calculated by multiplying the intensity score by the percentage score, resulting in a range of 0–12 scale. All IHC slides were independently evaluated by two pathologists blinded to clinical information, and any discrepancies were resolved through consensus review.

### Statistical analysis

All experiments were repeated at least three times. Data were analyzed using SPSS software (Version 28.0, IBM Corp, IBM SPSS 22.0, SPSS IncArmonk, NY, USAChigaco, IL). The results were expressed as mean ± standard deviation (SD), and comparisons between two groups were made by independent *t*-test, and comparisons of multiple groups were performed by one-way ANOVA. Correlations between clinical characteristics and gene expression were determined by χ^2^ test. The correlation between PROM2 mRNA expression and OS in BC patients was analyzed using Kaplan-Meier (KM) curves. *P* < 0.05 was considered as a statistically significant difference.

## Results

### PROM2 expression and prognostic relevance in BC cohort from public database

**Figure 1:**
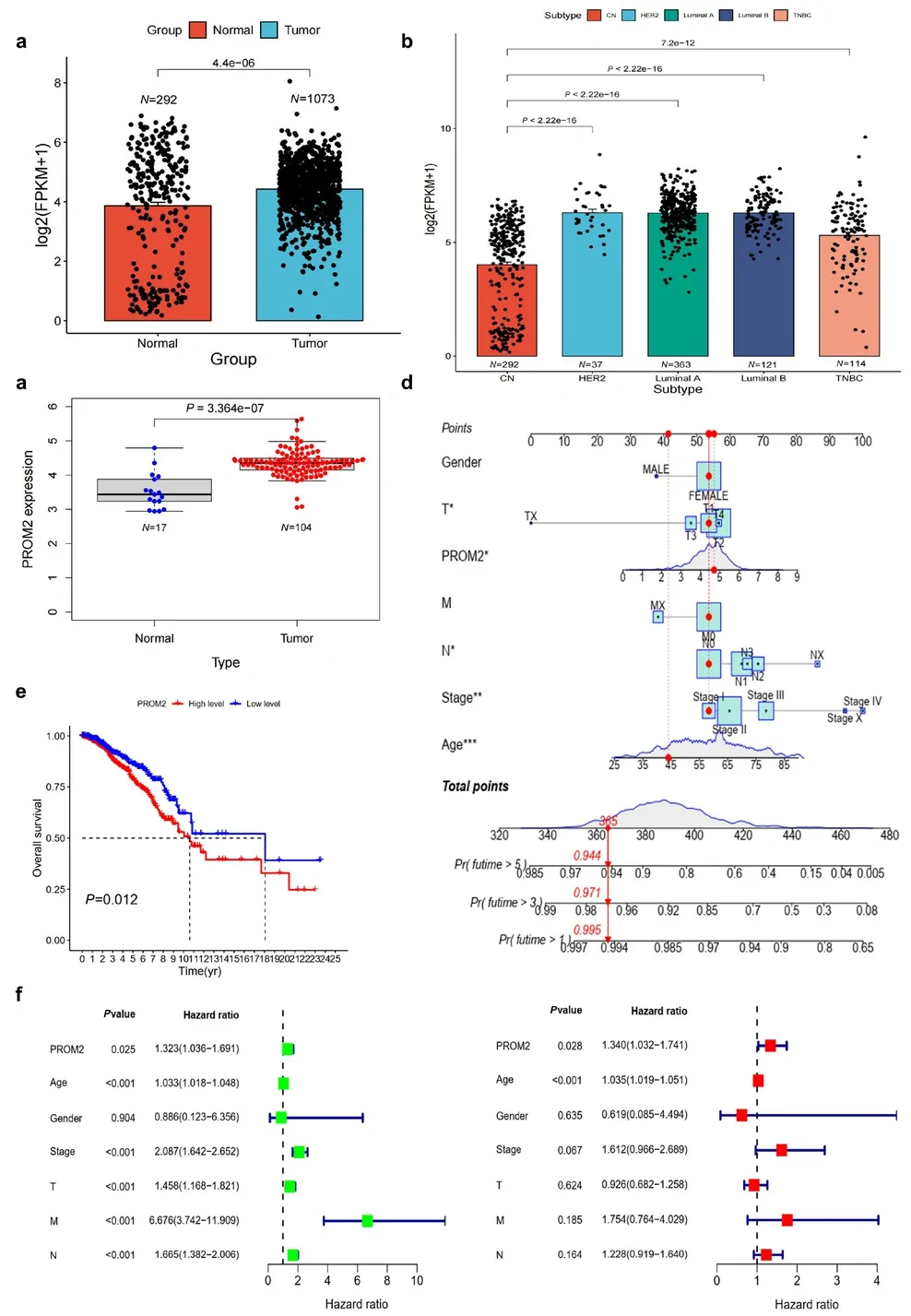
Biosignal analysis revealed that PROM2 was highly expressed in BC tissues and cells. (a) Analysis of TCGA-BC and GTEx datasets revealed high expression of PROM2 in BC tumor tissues compared to normal breast tissues (*P* = 4.4e-06). (b) PROM2 expression across different BC subtypes (ER-positive, HER2, Luminal A, Luminal B, and triple-negative) showed significantly higher levels compared to normal tissues. (c) Analysis of the GEO dataset GSE42568 using GEO profiles revealed that PROM2 expression was significantly higher in BC tissues compared to normal tissues (*P* = 3.364e-07). (d) Nomogram for predicting overall survival in BC patients based on clinical variables including gender, T stage, PROM2 expression level, M stage, N stage, clinical stage, and age. The nomogram allows for the calculation of total points and corresponding survival probabilities at 3-year, 5-year, and 10-year time points. (e) Kaplan-Meier survival analysis (https://kmplot.com/analysis/) showed that overall survival (OS) was worse in BC patients with high expression of PROM2 (*P* = 0.012). (f) Univariate (left panel) and multivariate (right panel) Cox regression analyses reveal PROM2 as an independent prognostic factor for BC. ^**^P < 0.05, ^**^P < 0.01, ^***^P < 0.001. BC: breast cancer; GEO: gene expression omnibus; PROM2: prominin 2.

Upon analyzing the comprehensive gene expression data from The Cancer Genome Atlas (TCGA) database, which included 1,073 BC tissues and 292 normal breast tissues (112 from TCGA and 180 from GTEx), we uncovered a significantly elevated expression of PROM2 in BC tissues compared to normal tissues (Figure 1A, *P* = 4.4e-06). We further stratified the BC samples by subtypes, specifically estrogen receptor-positive (ER+), HER2, Luminal A, Luminal B, and triple-negative breast cancer (TNBC), and found that PROM2 expression was significantly higher in all subtypes compared to normal tissues (Figure 1B). Additionally, analysis of the GSE42568 dataset reinforced our findings, showing a statistically significant upregulation of PROM2 expression in BC tissues relative to normal tissues (Figure 1C, *P* = 3.364e-07). To elucidate the prognostic implications of PROM2 in BC, we performed a KM survival analysis. High PROM2 expression was associated with a significant reduction in the overall survival of BC patients (Figure 1E, *P* = 0.012). Furthermore, we assessed the predictive power of PROM2 expression combined with clinical features on prognosis by constructing a nomogram. The nomogram demonstrates that PROM2 expression level is a significant contributor to the overall survival prediction model, with higher PROM2 expression associated with worse prognosis when combined with other clinical variables including T stage, N stage, M stage, clinical stage, gender, and age. The model shows good predictive accuracy with survival probabilities calculated at 3-year, 5-year, and 10-year time points, indicating that PROM2 can serve as a valuable prognostic biomarker in BC patients (Figure 1D). Corroborating these findings, both univariate and multivariate Cox regression analyses identified PROM2 as an independent prognostic factor significantly associated with BC progression and survival (Figure 1F). These findings underscore the potential clinical utility of PROM2 as a prognostic biomarker and therapeutic target in BC management.

### PROM2 affects immune infiltration in BC

**Figure 2:**
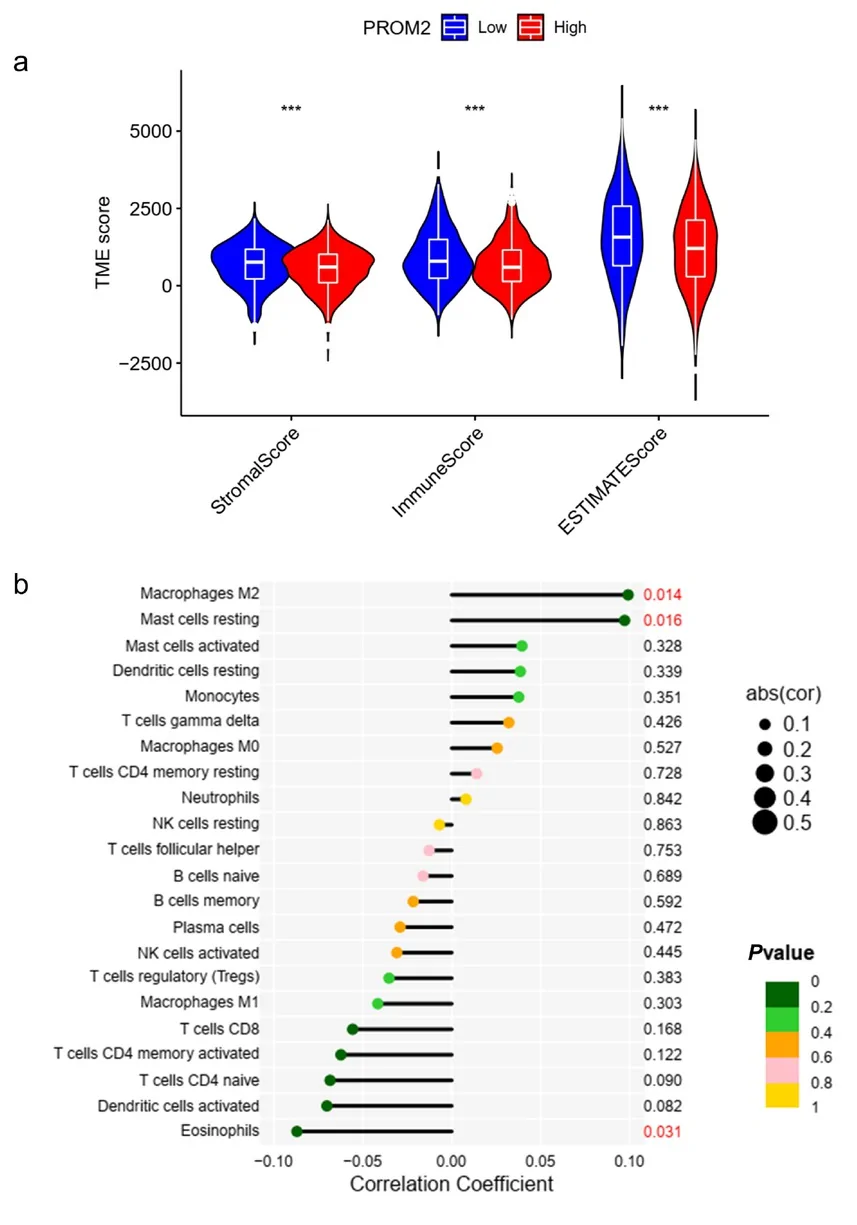
PROM2 affects BC immune infiltration. (a) Analysis of immune infiltration scores in the high and low-PROM2 expression groups revealed that PROM2 may influence stromal scores, and immune scores (*P* < 0.001). (b) PRM2 expression is positively or negatively associated with the infiltration of different immune cells. ^***^P < 0.001, compared with low-expression group. BC: breast cancer; PROM2: prominin 2.

CIBERSORT algorithm was employed to quantify immune cell infiltration levels in the TIMER database, and ESTIMATE software package was used to compute stromal and immune scores.^[24]^ A significant association between PROM2 expression levels and immune infiltration scores was found in BC. Notably, the PROM2-high expression group exhibited lower stromal and immune scores compared to the PROM2-low expression group (Figure 2A, File S1, *P* < 0.001). Furthermore, comprehensive immune deconvolution analyses with Spearman correlation analysis unveiled a positive correlation between M2 macrophage and mast cells with PROM2 expression, while the infiltration of CD4+ T cells and CD8+ T cells tended to negatively correlate with PROM2 expression, although the correlation was not statistically significant (Figure 2B, File S1). These findings collectively suggest a potential link between PROM2 expression and the tumor immune microenvironment, wherein PROM2 may preferentially modulate the infiltration of specific immune cell populations (such as M2 macrophages and mast cells) in BC microenvironment.^[27,28]^

### Validation of PROM2 overexpression in BC clinical samples

**Figure 3:**
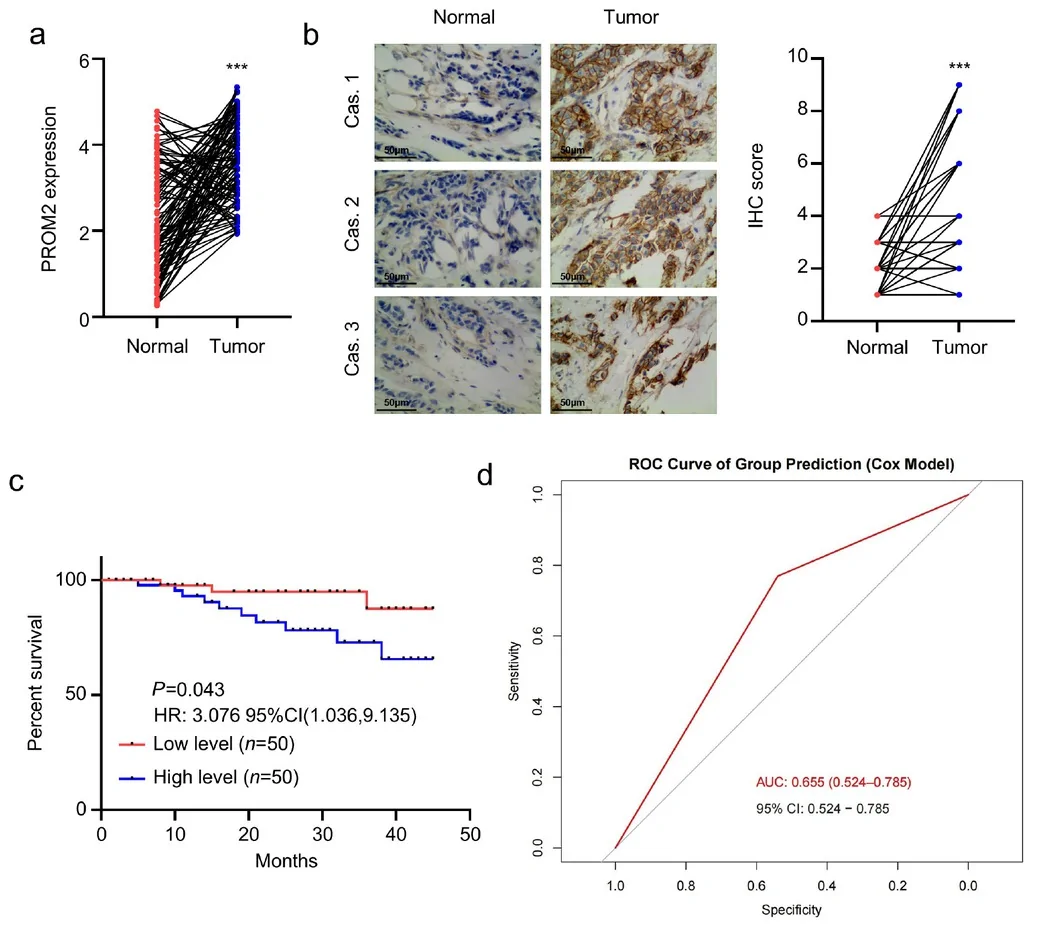
PROM2 is highly expressed in BC tissues. (a) qRT-PCR method was used to detect the expression level of PROM2 in 100 pairs of BC tissues and corresponding paracancerous tissues. (b): Ten pairs of BC cancer tissues and corresponding paracancerous tissues were subjected to PROM2 immunohistochemistry (IHC) staining. (c) According to the median value of PROM2 mRNA, 100 patients were classified into the PROM2 high-expression group (*n* = 50) and high-expression group (*n* = 50), and the association of PROM2 expression with the ovrall survival of the patients was plotted. (d) ROC curve of the Cox model for predicting group classification based on PROM2 expression levels. ^*^P < 0.05, ^***^P < 0.001, compared with normal tissues. BC: breast cancer; PROM2: prominin 2.

To substantiate the clinical relevance of PROM2 in BC, we extracted RNA from 100 pairs of BC tissues and adjacent non-cancerous paraneoplastic tissues for RT-qPCR analysis. There was a significantly higher expression of PROM2 in BC tissues compared to their paraneoplastic counterparts (Figure 3A, *P* < 0.001). Furthermore, we evaluated PROM2 protein levels in 10 pairs of BC tissues and matched paracancerous normal tissues using IHC. Quantitative analysis of the immunohistochemical results, performed using ImageJ software, unveiled a markedly elevated expression of PROM2 in tumor tissues relative to adjacent normal tissues (Figure 3B, *P* < 0.05). To explore the prognostic implications of PROM2 expression, we stratified 100 BC patients into PROM2 high-expression (*n* = 50) and low-expression (*n* = 50) groups based on the median PROM2 mRNA level. Kaplan-Meier survival analysis demonstrated a strong association between high PROM2 expression and reduced overall survival in BC patients (Figure 3C, *P* = 0.043, HR: 3.076, 95% CI: 1.036–9.135). Additionally, we evaluated the predictive performance of PROM2 expression using ROC analysis, yielding an AUC of 0.655 with a 95% CI: 0.524–0.785 (Figure 3D). Furthermore, correlation analysis showed that high PROM2 expression level was significantly correlated with larger tumor size, advanced clinical stage, and increased lymph node metastasis (Table 1). Additionally, multivariate Cox regression analysis indicated that PROM2 is an independent prognostic factor (Table 2). These findings further corroborate the clinical relevance of PROM2 in BC progression, which is consistent with a recent report implicating the role of PROM2 in BC.^[20]^

**Table 1:** Correlations between PROM2 expression and clinical characteristics in breast cancer patients

	Total (*n* = 100)	PROM2 expression Total		*P* value
Characteristic		Low (*n* = 50)	High (*n* = 50)	
Age (years)				0.677
≤50	36	17	19	
>50	64	33	31	
Menopausal status				0.157
Premenopausal	57	25	32	
Postmenopausal	43	25	18	
Tumor size (cm)				0.023^*^
≤2	63	37	26	
>2	37	13	24	
Pathological type				0.065
carcinoma Invasive ductal	39	15	24	
ductal Non-invasive carcinoma	61	35	26	
Clinical stage				0.026^*^
I-II	28	19	9	
III-IV	72	31	41	
Lymph node metastasis				0.002^**^
Negative	59	37	22	
Positive	41	13	28	
Histological grade				0.102
Low grade	16	5	11	
High grade	84	45	39	
TNM stage				0.087
I	23	15	8	
II	44	24	20	
III	28	10	18	
IV	5	1	4	
ER status				0.534
Negative	37	20	17	
Positive	63	30	33	
PR status				0.316
Negative	47	21	26	
Positive	53	29	24	
HER2 status				0.155
Negative	59	26	33	
Positive	41	24	17	

^*^P < 0.05, and ^**^P < 0.01. TNM: tumor node metastasis classification; ER: estrogen receptor; PR: progesterone receptor; HER2: human epidermal growth factor receptor 2.

**Table 2:** Univariable and multivariable regression analysis

	Univariable				Multivariable	
Characteristic	HR	95%CI	*P* value	HR	95%CI	*P* value
Age	1.073	1.005–1.145	0.036	1.043	0.983–1.107	0.160
Menopausal status	1.085	0.355–3.322	0.886			
Tumor size (cm)	3.210	1.540-6.681	0.002	1.450	0.622-3.390	0.392
Pathological type	0.594	0.183–1.933	0.387			
Clinical stage	0.318	0.070–1.438	0.137			
Lymph node metastasis	4.320	2.154-8.682	0.000	3.873	1.765-8.527	0.001
Histological grade	2.563	1.321-4.972	0.005	1.213	0.584-2.533	0.612
TNM stage	1.878	0.953–3.702	0.069			
ER status	1.909	0.640–5.689	0.246			
PR status	0.900	0.302–2.681	0.850			
HER2 status	0.583	0.196–1.737	0.332			
PROM2 expression	6.090	2.493–14.876	0.000	2.551	1.044–6.234	0.040

### Knockdown of PROM2 inhibits proliferation, apoptosis, migration and invasion of BC cells

**Figure 4:**
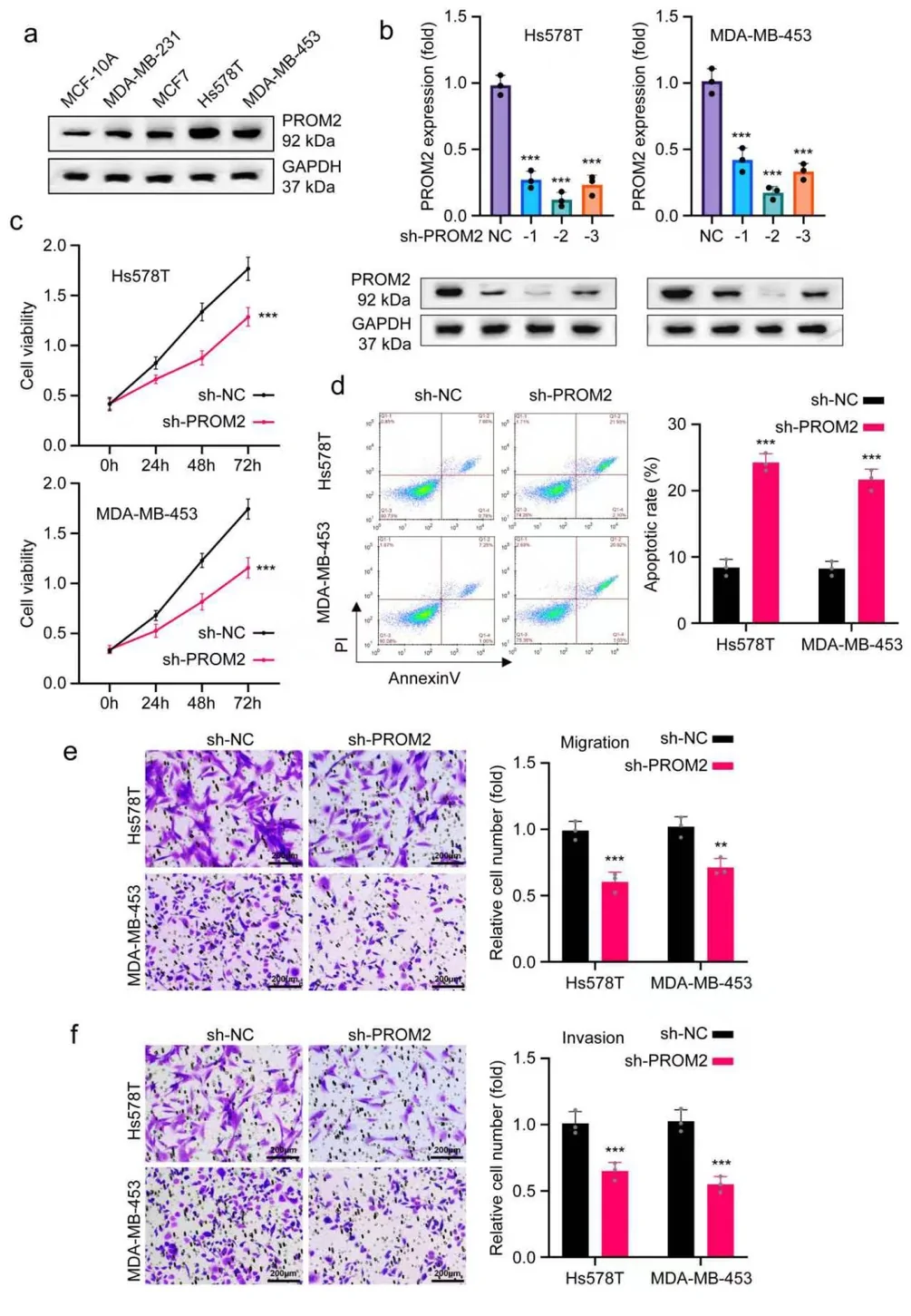
Knockdown of PROM2 inhibits proliferation, migration and invasion and promotes apoptosis in BC cells. (a) Western blot detection of PROM2 expression in normal breast epithelial cells MCF-10A and BC cells (MDA-MB-231, MCF-7, Hs578T, and MDA-MB-453 cells). (b) The knockdown efficiency was measured by the qRT-PCR and Western blot methods (compared with the sh-NC group). Lentivirus sh-PROM2#1, sh-PROM2#2, and sh-PROM2#3 were able to effectively knock down PROM2 expression, and sh-PROM2#2 with the highest knockdown efficiency was used for further experiment. (c) The CCK-8 method was used to detect the light absorption values at 450 nm for 0, 24, 48, and 72 h in different groups (sh-NC and sh-PROM2) of Hs578T and MDA-MB-453 cells. (d) AnnexinV-PI assay of apoptosis in Hs578T and MDA-MB-453 cells in different groups (sh-NC and sh-PROM2) at 24 h. (e) Transwell assay (without matrix gel EMC) detected the migration ability of cells in different groups (sh-NC and sh-PROM2) of Hs578T and MDA-MB-453 cells. (f) Transwell assay (with matrix gel EMC) detected the invasion ability of cells in different groups (sh-NC and sh-PROM2) cells. *N* = 3 independent experiments. ^**^P < 0.01; ^***^P < 0.001 compared with sh-NC group. PROM2: prominin 2.

To elucidate the functional role of PROM2 in BC, we next evaluated its expression levels across various BC cell lines in comparison to normal breast epithelial cells (MCF-10A). Western blot analysis revealed higher PROM2 expression in all BC cell lines examined (MDA-MB-231, Hs578T, MDA-MB-453, and MCF7) relative to the normal control. Notably, PROM2 expression was strongly elevated in highly invasive cell lines (MDA-MB-453, Hs578T) compared to the cell lines with low-invasive potential (MDA-MB-231, MCF7) (Figure 4A). Then lentiviral vector-mediated RNA interference was employed to knock down PROM2 expression in Hs578T and MDA-MB-453 cells. Both RT-qPCR and WB results demonstrated shRNA construct containing shPROM2 #2 showed the strongest knockdown effect, henceforth referred to as sh-PROM2 and utilized for subsequent experiments (Figure 4B). Hs578T and MDA-MB-453 cells were transduced with sh-PROM2 or a negative control sh-NC lentivirus, and cell viability was assessed using the CCK8 assay at 0, 24, 48, and 72 h. Remarkably, PROM2 knockdown significantly impaired cell proliferation compared to the control group (Figure 4C, *P* < 0.001). Furthermore, PROM2 silencing caused a significant increase of cell death in both Hs578T and MDA-MB-453 cells, as revealed by flow cytometry analysis (Figure 4D, *P* < 0.001). Meanwhile, Transwell assays (with or without Matrigel coating) showed that PROM2 knockdown profoundly inhibited the migratory (Figure 4E, *P* < 0.001) and invasive (Figure 4F, *P* < 0.001) capacities of Hs578T and MDA-MB-453 cells. Thus, PROM2 is required for the maintenance of the malignant properties of BC cells.

### Knockdown of PROM2 triggers ferroptosis in BC cells

**Figure 5:**
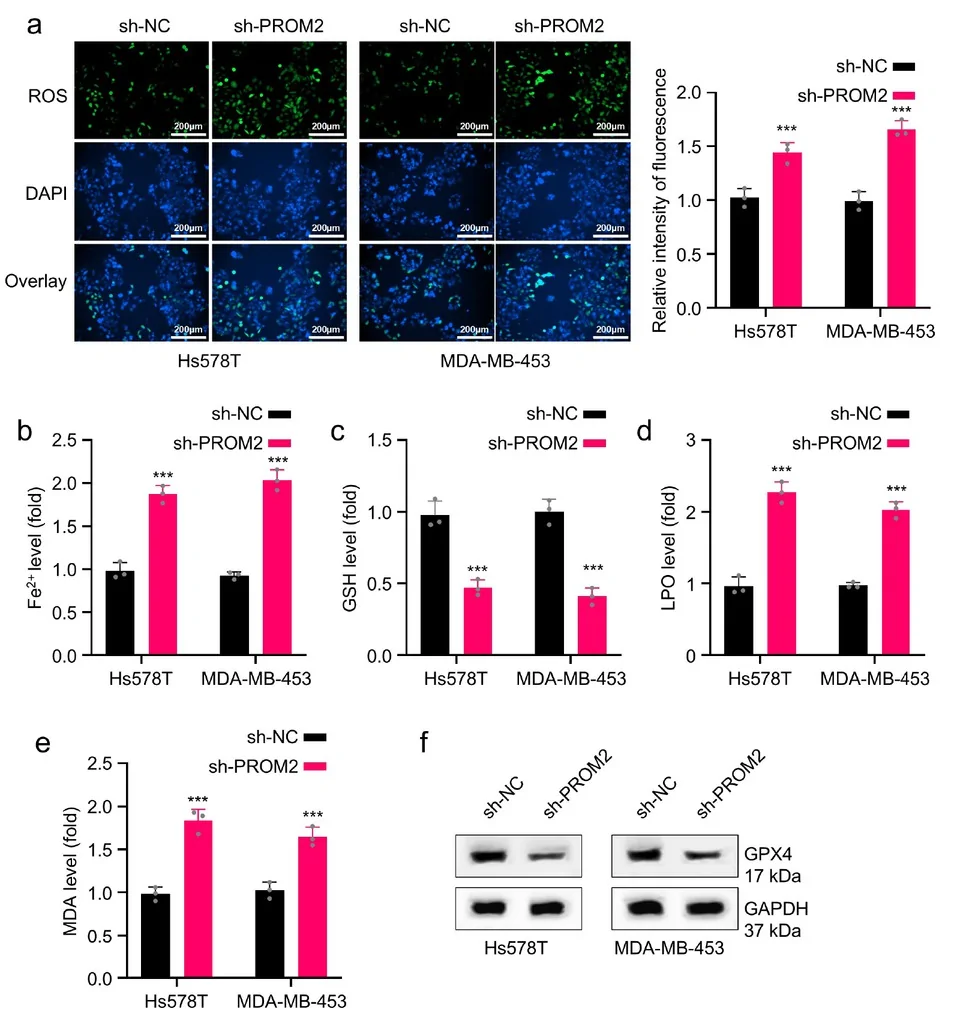
Knockdown of PROM2 increased intracellular ferroptosis levels. The levels of ROS, Fe^2+^, GSH, LPO, and MDA were detected in sh-NC, sh-PROM2 lentivirus-transduced Hs578T and MDA-MB-453 cells, respectively. Knockdown of PROM2 significantly increased the level of ROS fluorescence (a) and the level of Fe^2+^ (b), while significantly decreasing the GSH levels (c). Knockdown of PROM2 significantly increased the levels of LPO (d) and MDA (e). (f) Knockdown of PROM2 significantly decreased the expression levels of GPX4 as detected by WB. *N* = 3 independent experiments. ^***^P<0.001 compared with sh-NC group. GSH: glutathione; LPO: lipid peroxidation; PROM2: prominin 2.

To elucidate the potential mechanisms underlying the tumor-suppressive effects of PROM2 silencing, we investigated its impact on cellular redox homeostasis and ferroptosis induction. Employing the ROS detection reagent DCFH-DA, we observed a significant increase in ROS levels upon PROM2 knockdown in Hs578T and MDA-MB-453 cells compared to their respective sh-NC-transduced controls (Figure 5A, *P* < 0.001). Furthermore, assessment of intracellular ferrous ion levels revealed a marked elevation upon PROM2 silencing, indicative of increased cellular ferroptosis susceptibility (Figure 5B, *P* < 0.001). We also examined key indicators of ferroptosis, including GSH, LPO, and MDA levels, using biochemical assay kits. Notably, PROM2 knockdown significantly diminished GSH levels (Figure 5C, *P* < 0.001) while concomitantly upregulating LPO (Figure 5D, *P* < 0.001) and MDA (Figure 5E, P < 0.001) levels in Hs578T and MDA-MB-453 cells compared to their sh-NC controls. These findings suggest that knockdown of PROM2 sensitizes BC cells to oxidative stress and ferroptosis. Given the pivotal role of the enzyme GSH peroxidase 4 (GPX4) in regulating ferroptosis,^[9,10]^ we examined its protein expression upon PROM2 silencing. We observed a significant downregulation of GPX4 in sh-PROM2-transduced Hs578T and MDA-MB-453 cells compared to the control group (Figure 5F), further substantiating the heightened susceptibility to ferroptosis upon PROM2 depletion in BC cells.

### PROM2 activates the p38 MAPK signaling pathway to support the proliferation and attenuate oxidative stress in BC cells

**Figure 6:**
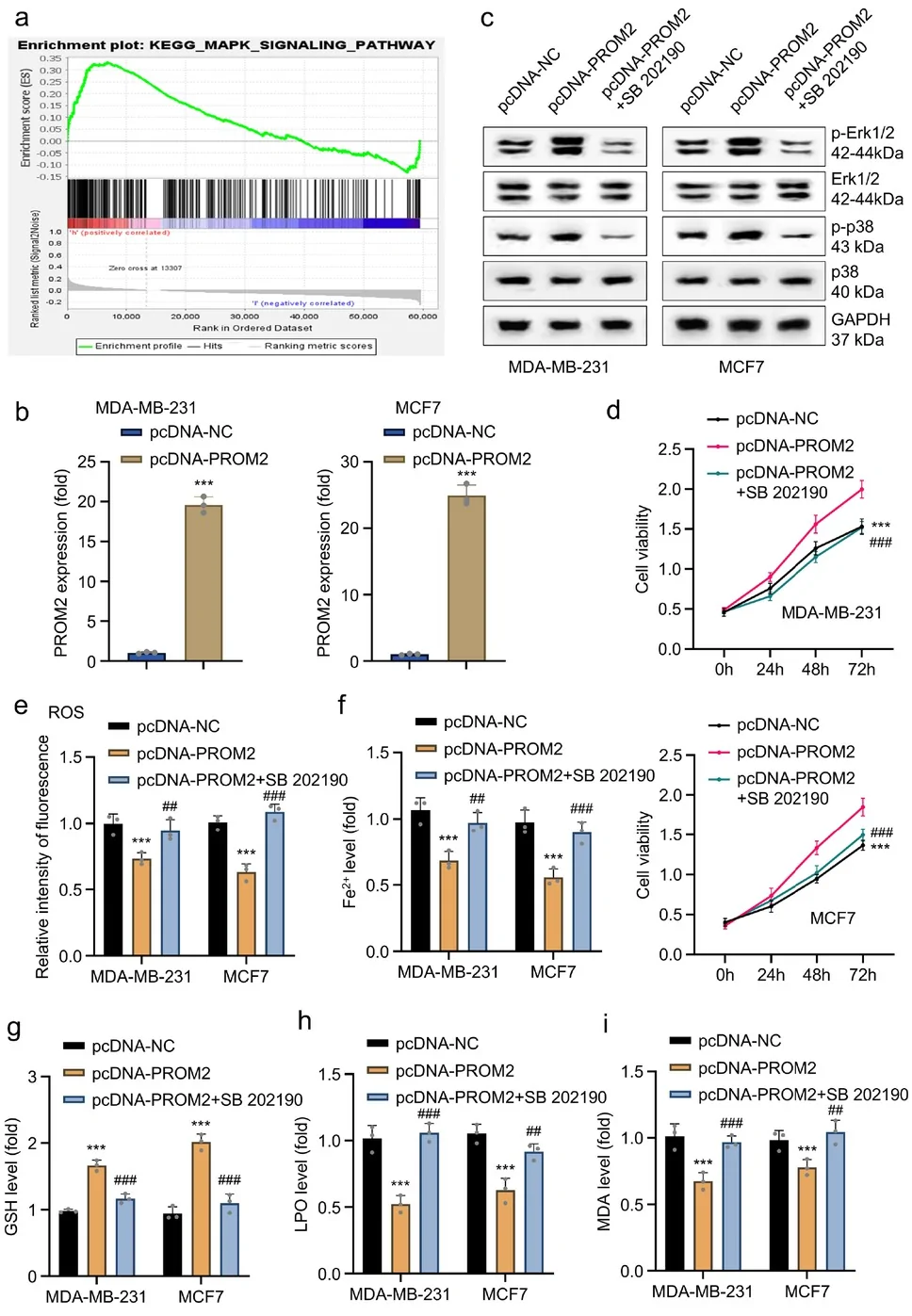
Overexpression of PROM2 activates the MAPK pathway in BC cells, and SB202190 (MAPK p38 inhibitor) partially reverses the effect of PROM2. (a) GSEA analysis showed that PROM2 was positively correlated with the MAPK pathway. (b) qRT-PCR method was used to detect mRNA levels of PROM2 in different groups (pcDNA-NC and pcDNA-PROM2) of MDA-MB-231 and MCF-7 cells. (c) WB was performed to assay protein levels of pERK1/2, p-P38 in MDA-MB-231 and MCF-7 cells of different groups (pcDNA-NC, pcDNA-PROM2, pcDNA-PROM2 + SB202190). (d) CCK-8 method for cell proliferation assessment in MDA-MB-231 and MCF-7 cells of different groups (pcDNA-NC and pcDNA-PROM2 and pcDNA-PROM2 + SB202190). (e) Cellular ROS measurement in pcDNA-NC and pcDNA-PROM2 and pcDNA-PROM2 + SB202190 groups. (f) Labile Fe^2+^ ion measurement in pcDNA-NC and pcDNA-PROM2 and pcDNA-PROM2 + SB202190 groups. (g) GSH level measurement in pcDNA-NC and pcDNA-PROM2 and pcDNA-PROM2 + SB202190 groups. (h) LPO detection in pcDNA-NC and pcDNA-PROM2 and pcDNA-PROM2 + SB202190 groups. (i) MDA level measurement in pcDNA-NC and pcDNA-PROM2 and pcDNA-PROM2 + SB202190 groups. *N* = 3 independent experiments. ^***^P < 0.001 compared with pcDNA-NC group and ^##^*P* < 0.01, ^###^*P* < 0.001 compared with pcDNA-PROM2 group. PROM2: prominin 2.

Gene set enrichment analysis (GSEA) unveiled a significant positive correlation between PROM2 expression and the MAPK signaling pathway in BC (Figure 6A). To further elucidate the mechanistic link between PROM2 and the MAPK pathway, we established PROM2-overexpressing MDA-MB-231 and MCF-7 cell models *via* transfection (Figure 6B, *P* < 0.001). Concurrently, we examined the expression levels of total and phosphorylated forms of ERK1/2 and p38 MAPK proteins in the control, PROM2-overexpressing, and PROM2-overexpressing + SB202190 (p38 inhibitor, 10 μmol/L) groups by Western blotting. Notably, PROM2 overexpression led to an increase in the phosphorylated levels of both ERK1/2 and p38 MAPK, which was significantly attenuated upon treatment with the p38 inhibitor SB 202190 (Figure 6C). To assess the functional consequences of PROM2 overexpression and MAPK pathway modulation, we conducted CCK8 cell growth assays. Overexpression of PROM2 significantly promoted cell proliferation, an effect that was abrogated by the addition of SB202190 (Figure 6D, *P* < 0.001). Furthermore, PROM2 overexpression reduced intracellular ROS and ferrous ion levels, which was also abolished upon SB202190 treatment (Figure 6E and 6F). In contrast, PROM2 overexpression led to a significant increase in GSH levels, and this was subsequently diminished upon treatment with SB 202190 (Figure 6G, *P* < 0.001). Conversely, the levels of LPO and MDA, key markers of oxidative stress and ferroptosis, were significantly reduced in the PROM2-overexpressing group (Figure 6H, *P* < 0.001 and Figure 6I, *P* < 0.001, respectively), an effect that was similarly reversed by SB 202190 treatment. We also examined the phosphorylation level of p38 in tumor and normal tissues, which revealed the increased phosphorylation of p38 in tumor tissues (Supplementary Figure S1A). Furthermore, there was a positive correlation between PROM2 expression and p-p38 level in the tumor tissues (Supplementary Figure S1B).

### Knockdown of PROM2 inhibits BC tumor growth in vivo

**Figure 7:**
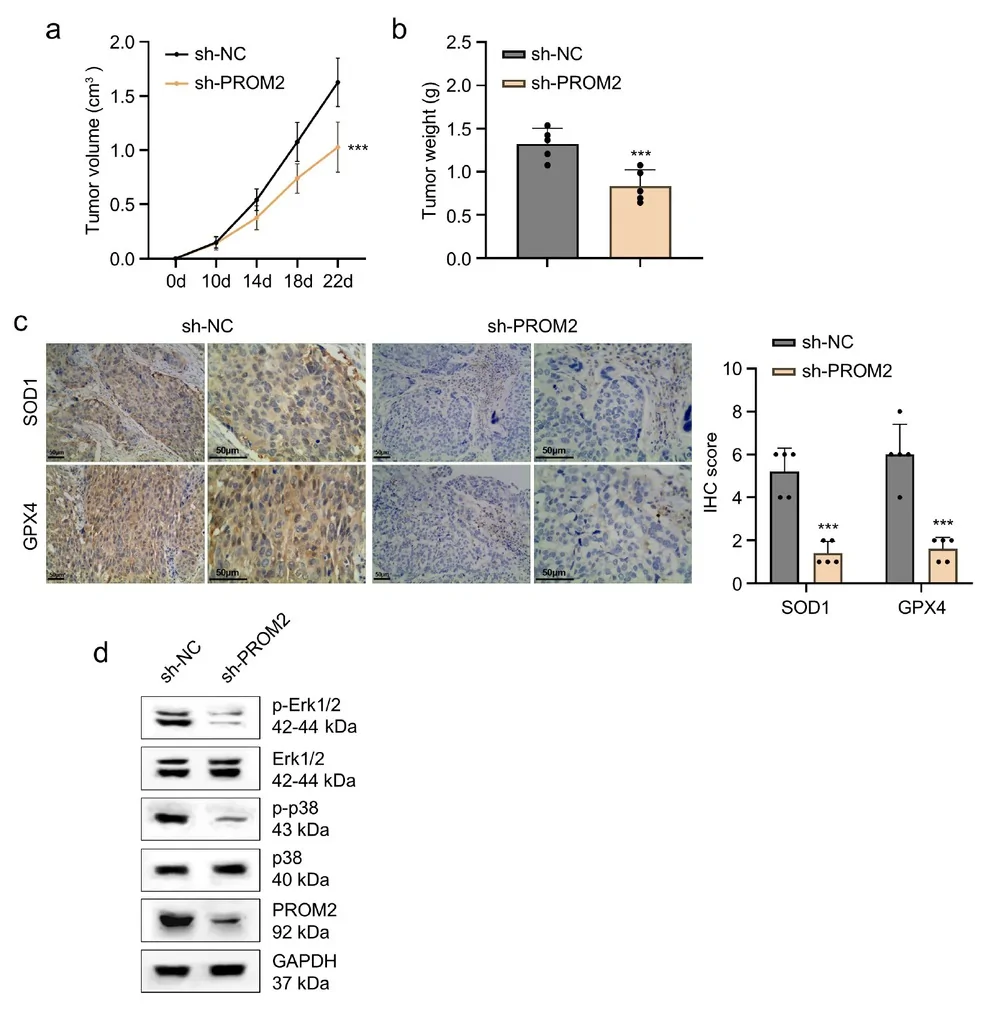
Knockdown of PROM2 inhibits BC cell proliferation *in vivo*. (a) 1 × 10^6^ Hs578T cells (sh-NC and sh-PROM2 groups, *n* = 5 mice in each group) were inoculated in nude mice as subcutaneous tumors, and the volume of subcutaneous tumors was monitored for 22 days. (b) Summary of subcutaneous tumor weights in sh-NC and sh-PROM2 groups. (c) IHC staining of SOD1 and GPX4 protein expression in subcutaneous tumor tissues of different groups (sh-NC and sh-PROM2). (d) WB method was used to detect PROM2, p-ERK1/2, p-P38 protein levels in tumor tissues. *N* = 5 animals in each group. ^*^P < 0.01, ^**^P < 0.01, and ^***^P < 0.001 compared with sh-NC group. PROM2: prominin 2.

To further elucidate the functional relevance of PROM2 *in vivo*, we established a xenograft BC model by subcutaneously injecting stable Hs578T cell lines transduced with either sh-NC or sh-PROM2 lentivirus into the right flanks of nude mice (*n* = 5 per group). Tumor growth was monitored by measuring the tumor volume every 4 days. PROM2 silencing significantly attenuated tumor growth, as evidenced by the reduced tumor volumes (Figure 7A, *P* < 0.001) and tumor weights (Figure 7B, *P* < 0.001) in the sh-PROM2 group compared to the sh-NC control group. Analysis of ferroptosis-related markers, SOD1 and GPX4, in the xenograft tumors by IHC showed a diminished expression levels of these proteins in the sh-PROM2 group (Figure 7C, *P* < 0.05), corroborating our previous *in vitro* findings and further implicating PROM2 in the regulation of ferroptosis. Additionally, we examined the phosphorylation levels of ERK1/2 and p38 MAPK in the xenograft tumors. Consistent with our *in vitro* observations, PROM2 silencing led to a significant reduction in the phosphorylation of both ERK1/2 and p38 MAPK (Figure 7D), further substantiating the involvement of the MAPK signaling pathway in mediating the pro-tumorigenic effects of PROM2. Furthermore, treatment with SB202190 (p38 inhibitor) also suppressed tumor growth of Hs578T cells in nude mice (Supplementary Figure S2).

## Discussion

BC is a prevalent malignancy that disproportionately affects women worldwide. Despite extensive research efforts, therapeutic options for advanced and recurrent BC remain limited, underscoring the urgent need for novel treatment strategies.^[1,5]^ The high mortality rate associated with BC, particularly in the aggressive triple-negative breast cancer (TNBC) subtype, is primarily attributed to the malignant biological behaviors such as invasion and metastasis.^[29]^ Non-apoptotic cell death pathways have been characterized and attracted intensive research in cancer biology. There is evidence showing the exquisite sensitivity of TNBC, a highly aggressive form of the disease with limited therapeutic options, to ferroptosis-inducing agents.^[15,16]^ Recent pan-cancer analyses have characterized cuproptosis and copper metabolism-related gene expression patterns across multiple cancer types.^[30, 31, 32]^ Additionally, emerging research on disulfidptosis has revealed another potential therapeutic avenue in cancer treatment,^[33]^ suggesting multiple cell death pathways could be exploited. These novel forms of non-apoptotic cell death have emerged as attractive therapeutic options, especially for the treatment of treatment-refractory tumors.

PROM2 (Prominin-2) is a pentameric transmembrane glycoprotein that exhibits widespread expression across various human tissues. Notably, accumulating evidence has implicated aberrant PROM2 expression in several malignancies, including lung, endometrial, and BC.^[34, 35, 36]^ Specifically, PROM2 is significantly upregulated and hypomethylated in patients with these cancers, correlating with reduced survival times.^[37]^ Consequently, PROM2 has emerged as a potential biomarker and therapeutic target in these malignancies. Our study unveils the critical role of PROM2 in promoting BC progression by modulating ferroptosis and the MAPK signaling pathway. PROM2 was found to be overexpressed in BC tissues, and its high expression correlated with poor prognosis and reduced patient survival. Knockdown of PROM2 in BC cells suppressed proliferation, migration, and invasion abilities, while inducing ferroptosis characterized by increased LPO, ROS, and diminished antioxidant defenses. Mechanistically, PROM2 activates the MAPK pathway, and inhibition of this pathway abrogated the pro-tumorigenic effects of PROM2 overexpression. Our findings are consistent with previous reports of the anti-ferroptosis role of PROM2,^[19,21,38]^ suggesting targeting PROM2 as a potential therapeutic strategy to induce ferroptosis and impede BC progression.

Immune scores can provide valuable predictions as markers for tumor patients and prognosis and are of increasing interest.^[39]^ Recent studies have demonstrated that immune cell infiltration patterns and immune scoring models can effectively predict BC prognosis and chemotherapy responses,^[40]^ while stromal-immune cell composition in inflammatory BC has been linked to improved outcomes and PD-L1 expression.^[41]^ Our analysis of immune cell infiltration analysis showed significant associations between PROM2 expression and stromal/ immune scores, which serve as surrogate measures of the non-tumor and immune components within the tumor microenvironment. Of note, there was a positive correlation between M2 macrophage and mast cells with PROM2 expression, while the infiltration of CD4+ T cells and CD8+ T cells tended to negatively correlate with PROM2 expression. These data suggest that PROM2 overexpression favor an immune-suppressive microenvironment by recruiting tumor-associated M2 macrophages,^[27]^ while dampening the infiltration of CD4+ and CD8+ T cells. Noteworthy, ferroptosis of cancer cells can release immunogenic substance to trigger immune cell activation.^[42,43]^ On the other hand, CD36, a fatty acid transporter, promotes ferroptosis in CD8+ T cells within the tumor microenvironment, thereby compromising their antitumor immune functions,^[44]^ which may contribute to CD8+ T cell exhaustation.^[45]^ Thus, PROM2 overexpression in BC may suppress immune stimulation in the tumor microenvironment by preventing immunogenic ferroptosis.

Ferroptosis, an iron-dependent form of cell death, is driven by excessive LPO.^[9,10]^ This process occurs when oxidizable lipids, such as polyunsaturated phospholipids, become over-accumulated. Without sufficient detoxification of LPO by the enzyme GPX4, this leads to the oxidative degradation of plasma membranes and the membranes of other internal organelles.^[46]^ If GPX4 activity is inhibited, physiological stresses, such as cell detachment from the extracellular matrix, can increase ROS levels and trigger ferroptosis.^[47]^ Previous studies have reported that the long non-coding RNA RP11-89 inhibits ferroptosis through upregulating PROM2 in bladder tumors.^[48]^ Furthermore, PROM2 overexpression enhances metastatic potential and confers ferroptosis resistance in diffident cancer cells.^[38]^ In agreement with these studies, we found that silencing of PROM2 increased the levels of ROS and ferrous ions in BC cells, and suppressed GPX4 expression. These data suggest that PROM2 knockdown promotes ferroptosis in BC cells. Consequently, targeting PROM2 may represent a potential pathway to induce ferroptosis in BC.

Numerous studies have demonstrated the implications of mitogen-activated protein kinase (MAPK)/extracellular signal-regulated kinase 1/2 (ERK1/2) and PI3K/AKT pathway in cancer progression.^[49, 50, 51]^ This pathway can be induced by prolactin (PRL) and epidermal growth factor (EGF) through their respective receptors, promoting cancer cell proliferation, survival, and metastasis.^[52,53]^ In this study, analysis of BC data from the TCGA database revealed that PROM2 expression increase was associated with MAPK signaling activation. By overexpressing PROM2 expression in BC cells, we showed that PROM2 increased the levels of activated phosphorylated ERK1/2 (P-ERK1/2) and phosphorylated p38 (P-p38). Collectively, we concluded that PROM2 expression is upregulated in BC, promoting proliferation, migration, and invasion by suppressing ferroptosis *via* the MAPK signaling pathway.

While this study provides insights into the role of PROM2 in facilitating BC progression by modulating ferroptosis *via* the MAPK pathway, certain limitations should be acknowledged. The primary reliance on *in vitro* experiments using BC cell lines may not fully recapitulate the complex tumor microenvironment, necessitating complementary *in vivo* studies with animal models or patient-derived xenografts. Furthermore, the mechanism by which PROM2 regulates GPX4 expression should be investigated in the future studies. Additionally, the intermediate molecular links underlying PROM2-mediated MAPK activation remain undefined in our current study, leading to an incomplete mechanistic framework that warrants further investigation to identify the specific upstream and downstream effectors involved in this signaling cascade. Furthermore, focusing solely on the MAPK pathway as the mediator of PROM2’s effects may overlook other potential signaling cascades involved in ferroptosis regulation and BC progression. Moreover, larger-scale clinical studies with diverse BC patient cohorts are warranted to validate the prognostic utility of PROM2 and establish its clinical relevance as a potential therapeutic target or biomarker. In particular, comprehensive subgroup analyses comparing PROM2’s prognostic and functional roles across different molecular subtypes (triple-negative, HER2-positive, and hormone receptor-positive) will be essential to determine whether PROM2-targeted strategies might be more effective in specific patient populations. Such stratified analyses could provide valuable guidance for patient selection in future clinical trials and help optimize personalized therapeutic approaches.

**Figure 8:**
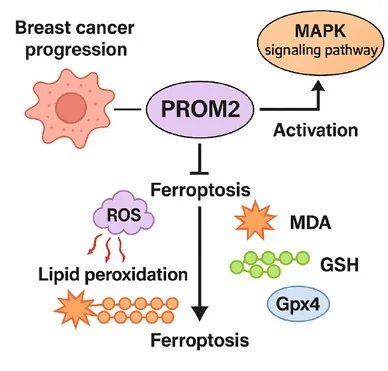
Mechanistic model of PROM2-mediated regulation of BC progression. PROM2 acts as a novel driver of BC progression through dual mechanisms: (1) inhibition of ferroptosis by suppressing ROS accumulation, lipid peroxidation, and MDA formation while maintaining GSH levels and Gpx4 expression, and (2) activation of the MAPK signaling pathway. BC: breast cancer; PROM2: prominin 2. GSH: glutathione; MDA: malondialdehyde; MAPK: mitogen-activated protein kinase.

In summary, our study reveals PROM2 as a novel driver of BC progression by inhibiting ferroptosis and activating the MAPK signaling pathway, where PROM2 knockdown suppressed BC cell proliferation, migration, and invasion while promoting ferroptosis (Figure 8). Targeting PROM2 emerges as a potential therapeutic approach for BC, warranting further investigation into its clinical utility and underlying molecular mechanisms.

## Supplementary Material

Supplementary Material Details
