# Supplementary material for: Prominin 2 promotes the progression of breast cancer by inhibiting ferroptosis via MAPK signaling
Source: J Transl Int Med. 2026 Jun 22;14(4):630–46. doi: 10.1515/jtim-2026-0061 (PMC13508830; doi:10.1515/jtim-2026-0061)
Supplement: Supplementary file 1 [file jtim-2026-0061_sm.pdf]

Supplementary materials

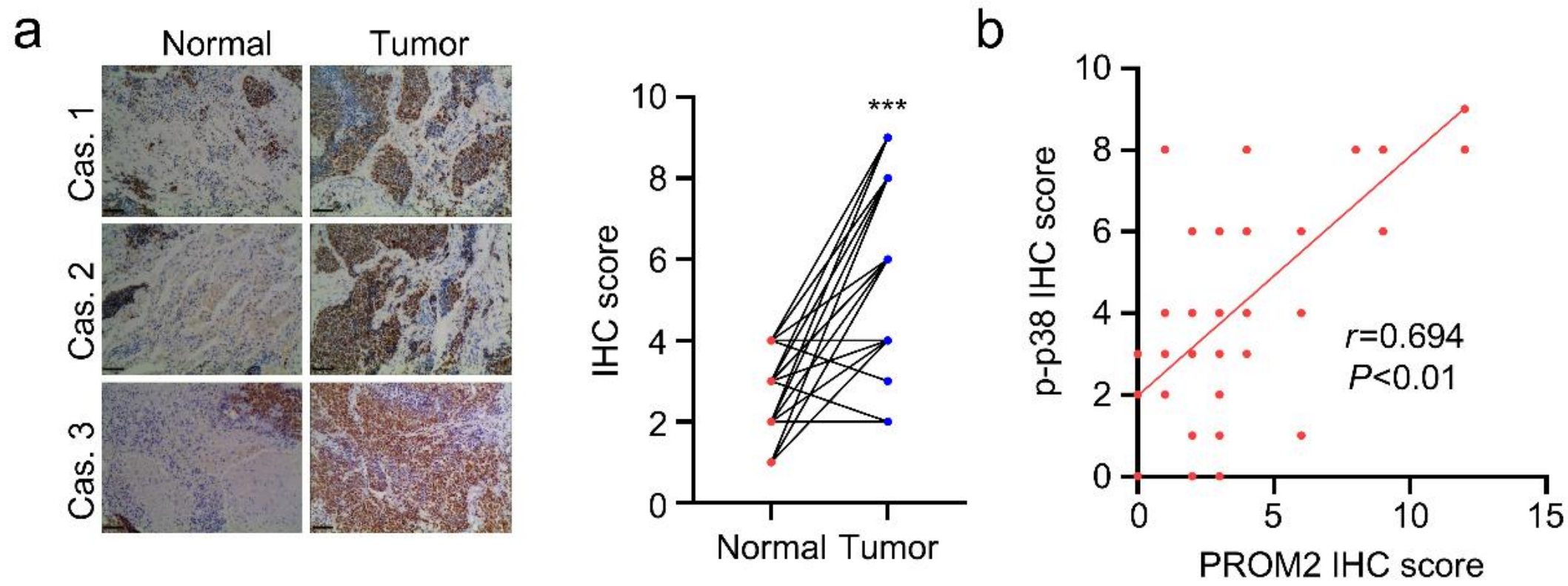

Supplementary Figure S1: Correlation between PROM2 expression of p38 phosphorylation in BC. a: IHC examination of phosphorylation level of p38 in tumor and normal tissues. 3 pairs of samples were shown. b. Correlation between PROM2 expression and p-p38 level in the tumor tissues. ***$P < 0.001$ compared with normal tissues.

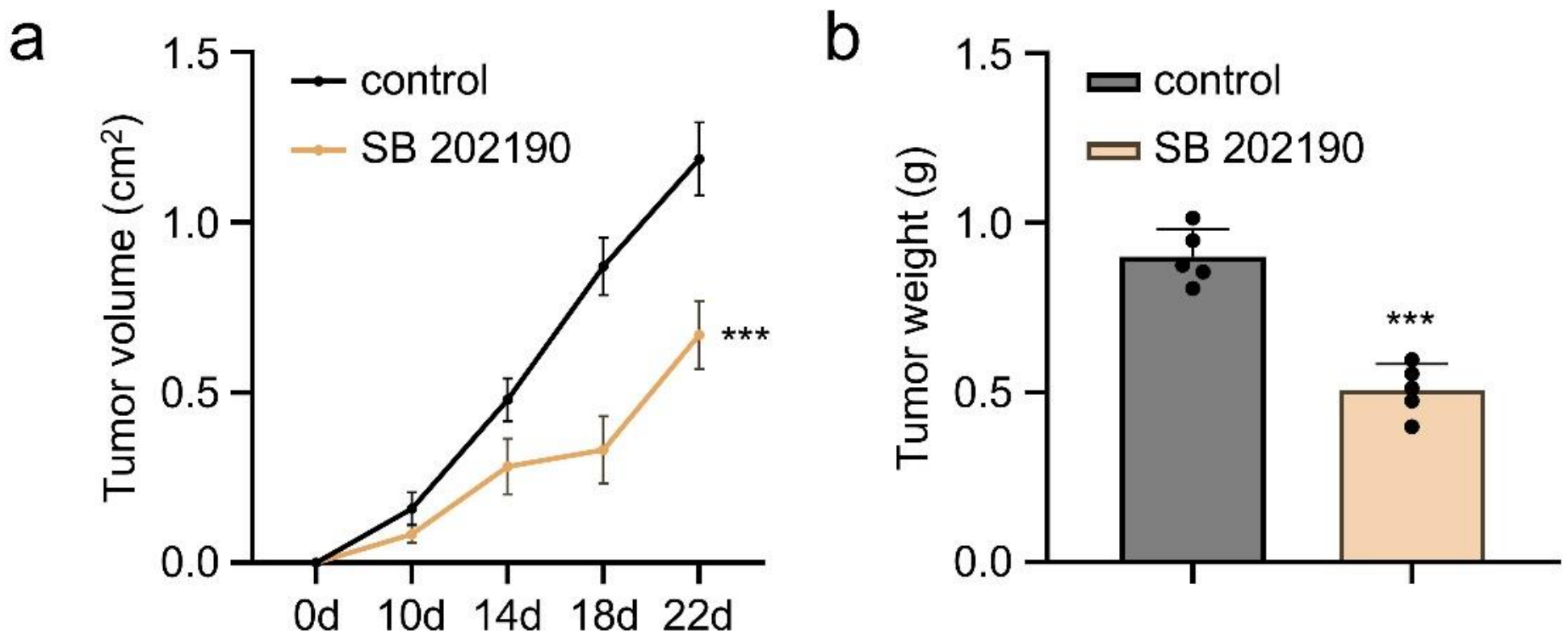

Supplementary Figure S2: SB202190 (p38 inhibitor) suppresses tumor growth of Hs578T cells in nude mice. a: Tumor growth volume in the control and SB202190 (p38 inhibitor) group; b: Tumor weight summary in the control and SB202190 (p38 inhibitor) group. ***$P < 0.001$ compared with control group.

Supplementary Table S1: geneExp

ID	PROM2	Type
TCGA-BH-A1FU-11A-23R-A14D-07	4.26204221188742	Normal
TCGA-BH-A1FC-11A-32R-A13Q-07	4.26269623455259	Normal
TCGA-AC-A2FM-11B-32R-A19W-07	4.790313305	Normal
TCGA-BH-A0DO-11A-22R-A12D-07	4.09145442818565	Normal
TCGA-E2-A1BC-11A-32R-A12P-07	0.164142648848306	Normal
TCGA-BH-A0BJ-11A-23R-A089-07	4.42215897991548	Normal
TCGA-E2-A1LH-11A-22R-A14D-07	4.71297538709068	Normal
TCGA-BH-A1FB-11A-33R-A13Q-07	4.52007017540776	Normal
TCGA-BH-A18M-11A-33R-A12D-07	3.96295614047043	Normal
TCGA-BH-A0BM-11A-12R-A089-07	4.28709546668488	Normal
TCGA-E2-A153-11A-31R-A12D-07	4.8147144732719	Normal
TCGA-BH-A0H5-11A-62R-A115-07	4.39770029964432	Normal
TCGA-BH-A1FG-11B-12R-A13Q-07	3.2066432239834	Normal
TCGA-A7-A0DB-11A-33R-A089-07	0.280243695866017	Normal
TCGA-BH-A1EU-11A-23R-A137-07	4.20385108421244	Normal
TCGA-BH-A0BT-11A-21R-A12P-07	4.91954031042293	Normal
TCGA-E2-A158-11A-22R-A12D-07	1.74045269846872	Normal
TCGA-E9-A1N9-11A-71R-A14D-07	3.79719978955893	Normal
TCGA-E9-A1NF-11A-73R-A14D-07	0.158207747625736	Normal
TCGA-GI-A2C9-11A-22R-A21T-07	3.80440468770764	Normal
TCGA-E9-A1RF-11A-32R-A157-07	0.104739216216237	Normal

TCGA-BH-A0DD-11A-23R-A12P-07	0.386038359888614	Normal
TCGA-BH-A208-11A-51R-A157-07	3.71786831273266	Normal
TCGA-BH-A0HA-11A-31R-A12P-07	3.84223467172341	Normal
TCGA-BH-A18J-11A-31R-A12D-07	4.08862501549948	Normal
TCGA-BH-A0B8-11A-41R-A089-07	0.762221231959501	Normal
TCGA-E9-A1RI-11A-41R-A169-07	0.808673354903202	Normal
TCGA-BH-A18Q-11A-34R-A12D-07	4.28002093054573	Normal
TCGA-BH-A1F8-11B-21R-A13Q-07	4.02462996002574	Normal
TCGA-BH-A1FJ-11B-42R-A13Q-07	3.55498170236685	Normal
TCGA-BH-A18R-11A-42R-A12D-07	3.27297869537357	Normal
TCGA-E9-A1N5-11A-41R-A14D-07	4.47389079230572	Normal
TCGA-BH-A1FR-11B-42R-A13Q-07	4.2191142973477	Normal
TCGA-A7-A0DC-11A-41R-A089-07	4.27693580936239	Normal
TCGA-BH-A0DK-11A-13R-A089-07	4.68941099302623	Normal
TCGA-E9-A1ND-11A-43R-A144-07	3.23404139971217	Normal
TCGA-E2-A1LB-11A-22R-A144-07	4.13688860790307	Normal
TCGA-BH-A0H9-11A-22R-A466-07	0.140516918655647	Normal
TCGA-BH-A0DG-11A-43R-A12P-07	4.236990014	Normal
TCGA-A7-A0D9-11A-53R-A089-07	0.486353976002037	Normal
TCGA-BH-A1EW-11B-33R-A137-07	3.98890277851356	Normal
TCGA-BH-A0DQ-11A-12R-A089-07	4.37169824670375	Normal
TCGA-BH-A0B5-11A-23R-A12P-07	0.119422582647884	Normal

TCGA-E9-A1NA-11A-33R-A144-07	4.3533232911629	Normal
TCGA-BH-A1F6-11B-94R-A13Q-07	3.57140984422145	Normal
TCGA-A7-A0CE-11A-21R-A089-07	4.79007866881152	Normal
TCGA-A7-A0CH-11A-32R-A089-07	3.62362108657226	Normal
TCGA-E2-A1IG-11A-22R-A144-07	3.76674355770148	Normal
TCGA-BH-A18L-11A-42R-A12D-07	4.00766196915681	Normal
TCGA-BH-A0B7-11A-34R-A115-07	4.40924835687669	Normal
TCGA-E9-A1R7-11A-42R-A14M-07	1.75710861055239	Normal
TCGA-BH-A1F0-11B-23R-A137-07	3.88314179329301	Normal
TCGA-BH-A18U-11A-23R-A12D-07	4.63074909579958	Normal
TCGA-BH-A1FH-11B-42R-A13Q-07	4.20221395883732	Normal
TCGA-E2-A15K-11A-13R-A12P-07	4.04181244695049	Normal
TCGA-BH-A0B3-11B-21R-A089-07	4.59663703410207	Normal
TCGA-BH-A0H7-11A-13R-A089-07	4.14235625177273	Normal
TCGA-BH-A0HK-11A-11R-A089-07	4.81193849484694	Normal
TCGA-BH-A204-11A-53R-A157-07	1.73296378383329	Normal
TCGA-BH-A0BQ-11A-33R-A115-07	4.72650973481387	Normal
TCGA-BH-A0DP-11A-12R-A089-07	4.22863401371108	Normal
TCGA-BH-A203-11A-42R-A169-07	3.64962695459563	Normal
TCGA-BH-A0AU-11A-11R-A12P-07	4.14936505952875	Normal
TCGA-BH-A18S-11A-43R-A12D-07	4.43968297159452	Normal
TCGA-BH-A0AZ-11A-22R-A12P-07	4.42143202879824	Normal

TCGA-BH-A18P-11A-43R-A12D-07	2.86134005349479	Normal
TCGA-BH-A1FN-11A-34R-A13Q-07	3.52844629655248	Normal
TCGA-BH-A0C3-11A-23R-A12P-07	3.93991296421364	Normal
TCGA-A7-A13E-11A-61R-A12P-07	3.03655608213631	Normal
TCGA-E2-A1LS-11A-32R-A157-07	0.542307583510237	Normal
TCGA-BH-A0AY-11A-23R-A089-07	3.94287102981318	Normal
TCGA-AC-A2FB-11A-13R-A17B-07	3.41298470751484	Normal
TCGA-BH-A0E0-11A-13R-A089-07	5.08971423383107	Normal
TCGA-BH-A0BC-11A-22R-A089-07	4.09935412300214	Normal
TCGA-BH-A1FE-11B-14R-A13Q-07	3.33902333337275	Normal
TCGA-A7-A13G-11A-51R-A13Q-07	0.180275189611879	Normal
TCGA-AC-A23H-11A-12R-A157-07	3.79227232748452	Normal
TCGA-BH-A1FM-11B-23R-A13Q-07	3.79708562971389	Normal
TCGA-BH-A0BV-11A-31R-A089-07	4.45782408570833	Normal
TCGA-BH-A0DH-11A-31R-A089-07	3.54485627488998	Normal
TCGA-BH-A0DZ-11A-22R-A089-07	5.1799210295107	Normal
TCGA-E2-A15M-11A-22R-A12D-07	3.67511586234266	Normal
TCGA-E9-A1RC-11A-33R-A157-07	0.147046143417315	Normal
TCGA-E2-A15I-11A-32R-A137-07	1.48424126925153	Normal
TCGA-BH-A1F2-11A-32R-A13Q-07	3.31621816492538	Normal
TCGA-BH-A18V-11A-52R-A12D-07	3.69175747504129	Normal
TCGA-BH-A1ET-11B-23R-A137-07	3.55846357253776	Normal

TCGA-GI-A2C8-11A-22R-A16F-07	0.0683268614345055	Normal
TCGA-E9-A1RH-11A-34R-A169-07	1.63226821549951	Normal
TCGA-BH-A0BZ-11A-61R-A12P-07	3.64334827172151	Normal
TCGA-BH-A209-11A-42R-A157-07	3.55385196818113	Normal
TCGA-E9-A1N4-11A-33R-A14M-07	4.12321921618019	Normal
TCGA-E2-A1L7-11A-33R-A144-07	4.78110262859458	Normal
TCGA-BH-A0BS-11A-11R-A12P-07	4.55595109471549	Normal
TCGA-BH-A1EO-11A-31R-A137-07	0.0764222754586024	Normal
TCGA-BH-A18K-11A-13R-A12D-07	4.42326216868335	Normal
TCGA-E9-A1NG-11A-52R-A14M-07	2.42502954752629	Normal
TCGA-E9-A1RD-11A-33R-A157-07	2.68927679299752	Normal
TCGA-BH-A18N-11A-43R-A12D-07	4.2131839485211	Normal
TCGA-A7-A13F-11A-42R-A12P-07	3.72026751909893	Normal
TCGA-BH-A1FD-11B-21R-A13Q-07	3.50964517572183	Normal
TCGA-BH-A1EV-11A-24R-A137-07	4.40932983246553	Normal
TCGA-AC-A2FF-11A-13R-A17B-07	4.75726865452394	Normal
TCGA-BH-A0BA-11A-22R-A19E-07	3.64287482344454	Normal
TCGA-BH-A0BW-11A-12R-A115-07	4.2606279081267	Normal
TCGA-BH-A0E1-11A-13R-A089-07	3.89641717678368	Normal
TCGA-BH-A0DT-11A-12R-A12D-07	4.82407026313141	Normal
TCGA-BH-A0C0-11A-21R-A089-07	4.12844188668221	Normal
TCGA-BH-A1EN-11A-23R-A13Q-07	3.34027740476625	Normal

TCGA-E9-A1RB-11A-33R-A157-07	2.71189131799661	Normal
TCGA-E9-A1N6-11A-32R-A144-07	3.88821720848535	Normal
TCGA-BH-A0DV-11A-22R-A12P-07	4.56092889762074	Normal
TCGA-B6-A0RH-01A-21R-A115-07	4.54595601721131	Tumor
TCGA-BH-A1FU-01A-11R-A14D-07	4.70445701932884	Tumor
TCGA-AR-A0TX-01A-11R-A084-07	6.04326137776808	Tumor
TCGA-A1-A0SE-01A-11R-A084-07	4.75524545985478	Tumor
TCGA-OL-A5D6-01A-21R-A27Q-07	5.65284210553073	Tumor
TCGA-E2-A1IK-01A-11R-A144-07	4.82355576535792	Tumor
TCGA-AN-A0FT-01A-11R-A034-07	4.46004135631002	Tumor
TCGA-A2-A0EO-01A-11R-A034-07	3.78572839295808	Tumor
TCGA-A2-A04P-01A-31R-A034-07	4.16467366374278	Tumor
TCGA-AC-A5EH-01A-11R-A28M-07	4.76549761770402	Tumor
TCGA-A7-A0DC-01B-04R-A22O-07	3.31407291464763	Tumor
TCGA-OK-A5Q2-01A-11R-A27Q-07	5.28251393953742	Tumor
TCGA-BH-A0DO-01B-11R-A12D-07	4.22968789281386	Tumor
TCGA-BH-A18J-01A-11R-A12D-07	5.36162450376311	Tumor
TCGA-A7-A0CJ-01A-21R-A00Z-07	3.67720377273297	Tumor
TCGA-A8-A09V-01A-11R-A034-07	4.44853098190579	Tumor
TCGA-C8-A275-01A-21R-A16F-07	4.22371600194861	Tumor
TCGA-E2-A1LA-01A-11R-A144-07	3.67140660940647	Tumor
TCGA-AC-A8OS-01A-12R-A41B-07	4.69737907016344	Tumor

TCGA-B6-A0WT-01A-11R-A109-07	4.76590598040793	Tumor
TCGA-EW-A1PG-01A-11R-A144-07	4.4897099045728	Tumor
TCGA-A8-A08H-01A-21R-A00Z-07	2.84617234705944	Tumor
TCGA-AC-A5XS-01A-11R-A29R-07	4.95541045548997	Tumor
TCGA-A8-A097-01A-11R-A034-07	4.87577514924867	Tumor
TCGA-E2-A15C-01A-31R-A12D-07	3.84466685269957	Tumor
TCGA-C8-A12M-01A-11R-A115-07	5.37394395786069	Tumor
TCGA-E2-A14Z-01A-11R-A115-07	5.09973282840123	Tumor
TCGA-A7-A4SF-01A-11R-A266-07	5.17044988664359	Tumor
TCGA-BH-A0W5-01A-11R-A109-07	3.89096219623443	Tumor
TCGA-BH-A0C7-01B-11R-A115-07	3.9762811412817	Tumor
TCGA-BH-A0HU-01A-11R-A034-07	4.96402890000206	Tumor
TCGA-E9-A226-01A-21R-A157-07	4.8308840334194	Tumor
TCGA-E2-A14T-01A-11R-A115-07	5.02736198140034	Tumor
TCGA-AN-A046-01A-21R-A034-07	5.17716835263478	Tumor
TCGA-BH-A1F5-01A-12R-A13Q-07	4.71293688082399	Tumor
TCGA-AO-A0J6-01A-11R-A034-07	5.56803210477128	Tumor
TCGA-AC-A23E-01A-11R-A157-07	3.75009193044865	Tumor
TCGA-E2-A1IH-01A-11R-A13Q-07	4.71587690133213	Tumor
TCGA-E2-A15T-01A-11R-A115-07	4.42736480955638	Tumor
TCGA-LL-A5YN-01A-11R-A28M-07	4.64704966492027	Tumor
TCGA-EW-A2FW-01A-11R-A17B-07	4.55993226353387	Tumor

TCGA-A7-A0DB-01C-02R-A277-07	3.56158275569523	Tumor
TCGA-A7-A0DB-01A-11R-A277-07	3.8305494319557	Tumor
TCGA-E9-A1R0-01A-22R-A16F-07	4.13626522570419	Tumor
TCGA-BH-A1F8-01A-11R-A13Q-07	5.56529861431701	Tumor
TCGA-BH-A0BP-01A-11R-A115-07	5.22485835170255	Tumor
TCGA-BH-A18R-01A-11R-A12D-07	5.46746860981744	Tumor
TCGA-D8-A147-01A-11R-A115-07	3.32702621697059	Tumor
TCGA-E9-A1N5-01A-11R-A14D-07	4.35252549459107	Tumor
TCGA-BH-A0HP-01A-12R-A084-07	5.43355039043305	Tumor
TCGA-A8-A06R-01A-11R-A00Z-07	3.86736276575328	Tumor
TCGA-A8-A086-01A-11R-A00Z-07	3.43593523416026	Tumor
TCGA-AR-A1AX-01A-11R-A12P-07	5.18779149622019	Tumor
TCGA-BH-A0B6-01A-11R-A19W-07	5.54476047112157	Tumor
TCGA-D8-A1JJ-01A-31R-A14M-07	5.52660408746781	Tumor
TCGA-OL-A66P-01A-11R-A31O-07	5.05417558323913	Tumor
TCGA-E9-A295-01A-11R-A16F-07	3.96459270368399	Tumor
TCGA-C8-A3M8-01A-11R-A213-07	4.6911935504198	Tumor
TCGA-AO-A1KO-01A-31R-A13Q-07	4.2553572118434	Tumor
TCGA-AN-A0FN-01A-11R-A034-07	3.19769243367353	Tumor
TCGA-BH-A0BM-01A-11R-A056-07	4.13450846421918	Tumor
TCGA-BH-A18F-01A-11R-A12D-07	6.27096243327069	Tumor
TCGA-E2-A14S-01A-11R-A12D-07	4.5942977281136	Tumor

TCGA-B6-A0RI-01A-11R-A056-07	4.8243452658934	Tumor
TCGA-A2-A3XW-01A-11R-A239-07	4.79274084821219	Tumor
TCGA-3C-AALK-01A-11R-A41B-07	4.23223095382554	Tumor
TCGA-A2-A0EU-01A-22R-A056-07	3.69543711040537	Tumor
TCGA-A2-A0YG-01A-21R-A109-07	5.45604715509123	Tumor
TCGA-AO-A0JJ-01A-11R-A056-07	3.92332035364441	Tumor
TCGA-C8-A131-01A-11R-A115-07	4.94773627960773	Tumor
TCGA-AR-A0TZ-01A-12R-A084-07	4.85647781953696	Tumor
TCGA-BH-A0EE-01A-11R-A034-07	4.07752501482842	Tumor
TCGA-E2-A15D-01A-11R-A115-07	4.90902900765537	Tumor
TCGA-AO-A1KQ-01A-11R-A13Q-07	3.63247762377457	Tumor
TCGA-A7-A426-01A-22R-A24H-07	4.46706423413086	Tumor
TCGA-BH-A1FG-01A-11R-A13Q-07	3.55224179672161	Tumor
TCGA-AQ-A0Y5-01A-11R-A14M-07	5.82502490892699	Tumor
TCGA-BH-A0DD-01A-31R-A12P-07	4.04942166633415	Tumor
TCGA-AR-A24L-01A-11R-A169-07	4.95018376641553	Tumor
TCGA-E2-A1B4-01A-11R-A12P-07	5.02851169077204	Tumor
TCGA-E9-A1RI-01A-11R-A169-07	3.42184266657591	Tumor
TCGA-AN-A0FW-01A-11R-A034-07	4.63950423497254	Tumor
TCGA-AN-A04A-01A-21R-A034-07	4.82259759743482	Tumor
TCGA-AO-A03T-01A-21R-A034-07	4.68882936938964	Tumor
TCGA-AO-A0JI-01A-21R-A056-07	5.2222945208513	Tumor

TCGA-BH-A0HA-01A-11R-A12P-07	3.13111201878936	Tumor
TCGA-BH-A0BF-01A-21R-A12P-07	5.0355447352694	Tumor
TCGA-A2-A1G4-01A-11R-A13Q-07	5.28040703524246	Tumor
TCGA-A2-A25B-01A-11R-A169-07	3.61705158593447	Tumor
TCGA-E2-A15O-01A-11R-A115-07	3.6315234064789	Tumor
TCGA-BH-A0EB-01A-11R-A034-07	4.19237567457562	Tumor
TCGA-E9-A1NC-01A-21R-A26B-07	3.11531629245978	Tumor
TCGA-AN-A0AR-01A-11R-A00Z-07	3.12603123015207	Tumor
TCGA-BH-A202-01A-11R-A14M-07	3.92753620894242	Tumor
TCGA-OL-A66N-01A-12R-A31O-07	4.20348308870996	Tumor
TCGA-EW-A1J1-01A-11R-A13Q-07	4.21553882390227	Tumor
TCGA-EW-A1P1-01A-31R-A14D-07	4.15826430885055	Tumor
TCGA-LL-A5YM-01A-11R-A28M-07	4.06458110919057	Tumor
TCGA-C8-A134-01A-11R-A115-07	3.99599999804396	Tumor
TCGA-AC-A3OD-01B-06R-A22O-07	3.38478202360611	Tumor
TCGA-A8-A08P-01A-11R-A00Z-07	4.41067351579374	Tumor
TCGA-A2-A04R-01A-41R-A109-07	3.15930625464117	Tumor
TCGA-BH-A1EU-01A-11R-A137-07	4.13660155745554	Tumor
TCGA-AR-A1AW-01A-21R-A12P-07	3.19459140277373	Tumor
TCGA-E2-A15P-01A-11R-A115-07	5.02409800916734	Tumor
TCGA-A2-A0YC-01A-11R-A109-07	3.71178121465911	Tumor
TCGA-AN-A0XU-01A-11R-A109-07	4.82168470747112	Tumor

TCGA-A8-A07S-01A-11R-A034-07	5.63988909988177	Tumor
TCGA-JL-A3YW-01A-12R-A239-07	4.55688918972687	Tumor
TCGA-C8-A1HF-01A-11R-A137-07	5.50455048037726	Tumor
TCGA-A8-A07Z-01A-11R-A00Z-07	5.04583609353236	Tumor
TCGA-A2-A0ES-01A-11R-A115-07	4.56833618213375	Tumor
TCGA-A1-A0SJ-01A-11R-A084-07	3.23786960086751	Tumor
TCGA-A7-A4SA-01A-11R-A266-07	3.94090905399914	Tumor
TCGA-BH-A0BT-01A-11R-A12P-07	4.39434950399155	Tumor
TCGA-EW-A1J2-01A-21R-A13Q-07	4.41009683533999	Tumor
TCGA-E9-A5UP-01A-11R-A28M-07	5.55517808760328	Tumor
TCGA-OL-A66O-01A-11R-A31O-07	3.55094990254589	Tumor
TCGA-AC-A3YI-01A-21R-A239-07	4.99662375281393	Tumor
TCGA-B6-A0WX-01A-11R-A109-07	4.02376100548023	Tumor
TCGA-AO-A0J9-01A-11R-A034-07	3.07936104275338	Tumor
TCGA-E9-A244-01A-11R-A169-07	1.84261685591857	Tumor
TCGA-EW-A424-01A-11R-A24H-07	5.40492014780752	Tumor
TCGA-BH-A0HN-01A-11R-A10U-07	4.96646738328846	Tumor
TCGA-AO-A03V-01A-11R-A115-07	5.44125106797668	Tumor
TCGA-AR-A1AH-01A-11R-A12D-07	4.98141886647078	Tumor
TCGA-BH-A18I-01A-11R-A12D-07	3.49151963494098	Tumor
TCGA-D8-A3Z6-01A-11R-A239-07	5.211580746	Tumor
TCGA-D8-A27I-01A-11R-A16F-07	4.44475319136307	Tumor

TCGA-A2-A3XS-01A-11R-A22U-07	2.78870658882774	Tumor
TCGA-A2-A04Y-01A-21R-A034-07	4.42044197859302	Tumor
TCGA-D8-A1JA-01A-11R-A13Q-07	5.34622649523793	Tumor
TCGA-E2-A152-01A-11R-A12D-07	5.0044654367703	Tumor
TCGA-AO-A0J4-01A-11R-A034-07	3.77255079692537	Tumor
TCGA-D8-A1XK-01A-21R-A14M-07	3.20839239979068	Tumor
TCGA-A2-A0CQ-01A-21R-A034-07	5.22610772551637	Tumor
TCGA-AR-A1AI-01A-11R-A12P-07	3.95579634889756	Tumor
TCGA-B6-A2IU-01A-32R-A18M-07	3.93516740742819	Tumor
TCGA-D8-A1JL-01A-11R-A13Q-07	4.15965340274601	Tumor
TCGA-AN-A0AK-01A-21R-A00Z-07	6.01941058871377	Tumor
TCGA-E9-A24A-01A-11R-A169-07	4.65089664696305	Tumor
TCGA-GI-A2C9-01A-11R-A21T-07	3.8008791974214	Tumor
TCGA-BH-A0W7-01A-11R-A115-07	4.48771143131893	Tumor
TCGA-BH-A0B5-01A-11R-A12P-07	5.26116219642928	Tumor
TCGA-A2-A0CZ-01A-11R-A034-07	4.32344212995092	Tumor
TCGA-A2-A0SV-01A-11R-A084-07	5.11534958875189	Tumor
TCGA-OL-A66L-01A-12R-A31O-07	3.8515191911699	Tumor
TCGA-AR-A2LO-01A-31R-A18M-07	5.11228727943647	Tumor
TCGA-D8-A1JU-01A-11R-A13Q-07	4.34666619426278	Tumor
TCGA-AC-A8OP-01A-11R-A36F-07	4.93906198448548	Tumor
TCGA-GM-A2DD-01A-11R-A180-07	4.8902083315976	Tumor

TCGA-A7-A5ZX-01A-12R-A29R-07	4.53453478458258	Tumor
TCGA-AO-A12H-01A-11R-A115-07	4.80317536305105	Tumor
TCGA-C8-A1HE-01A-11R-A13Q-07	4.99504576433318	Tumor
TCGA-BH-A0BD-01A-11R-A034-07	5.21251486713593	Tumor
TCGA-E9-A22B-01A-11R-A157-07	3.74904077084432	Tumor
TCGA-E9-A228-01A-31R-A157-07	4.79864676080604	Tumor
TCGA-E2-A153-01A-12R-A12D-07	4.57340482466276	Tumor
TCGA-A1-A0SD-01A-11R-A115-07	4.53384363737918	Tumor
TCGA-AC-A2FM-01A-11R-A19W-07	3.23024913459342	Tumor
TCGA-BH-A0B4-01A-11R-A00Z-07	4.37453874667881	Tumor
TCGA-A2-A0CX-01A-21R-A00Z-07	5.3516106627642	Tumor
TCGA-BH-A0AW-01A-11R-A056-07	4.92531587479002	Tumor
TCGA-A2-A0SU-01A-11R-A084-07	4.87358683690503	Tumor
TCGA-D8-A1JT-01A-31R-A13Q-07	4.50445514033579	Tumor
TCGA-E9-A1NI-01A-11R-A14D-07	4.33273657246386	Tumor
TCGA-OL-A5D7-01A-11R-A27Q-07	3.25177967695237	Tumor
TCGA-E2-A570-01A-11R-A29R-07	4.73715673859609	Tumor
TCGA-E2-A1LH-01A-11R-A14D-07	2.16883455319018	Tumor
TCGA-LL-A7T0-01A-31R-A352-07	5.45584337439172	Tumor
TCGA-A7-A4SE-01A-11R-A266-07	4.56972196302139	Tumor
TCGA-A2-A3XY-01A-11R-A239-07	4.88398242216022	Tumor
TCGA-C8-A26Z-01A-11R-A16F-07	3.44542213755914	Tumor

TCGA-AR-A1AV-01A-21R-A12P-07	5.0910142853671	Tumor
TCGA-E2-A10E-01A-21R-A10J-07	5.07715325503975	Tumor
TCGA-E2-A14P-01A-31R-A12D-07	4.86626509896028	Tumor
TCGA-A7-A4SC-01A-12R-A266-07	5.55872070141741	Tumor
TCGA-BH-A0RX-01A-21R-A084-07	3.23473122601661	Tumor
TCGA-E2-A15F-01A-11R-A115-07	5.10195256663982	Tumor
TCGA-E2-A107-01A-11R-A10J-07	5.34541056948753	Tumor
TCGA-E2-A109-01A-11R-A10J-07	3.72138359722945	Tumor
TCGA-E9-A3QA-01A-61R-A22K-07	3.66866377770022	Tumor
TCGA-E2-A576-01A-11R-A31O-07	3.92336789814371	Tumor
TCGA-A8-A099-01A-11R-A00Z-07	5.30315239985074	Tumor
TCGA-BH-A0HQ-01A-11R-A034-07	4.83156313715335	Tumor
TCGA-AN-A0XR-01A-11R-A109-07	4.33519738691076	Tumor
TCGA-S3-A6ZF-01A-32R-A32P-07	5.29621877395278	Tumor
TCGA-E9-A1RF-01A-11R-A157-07	4.42223972965542	Tumor
TCGA-D8-A27P-01A-11R-A16F-07	5.00499568902537	Tumor
TCGA-D8-A142-01A-11R-A115-07	3.90240164656464	Tumor
TCGA-AR-A0U3-01A-11R-A109-07	5.96698410787883	Tumor
TCGA-B6-A0RV-01A-11R-A084-07	5.35537576519781	Tumor
TCGA-E9-A22D-01A-11R-A157-07	3.98991102418616	Tumor
TCGA-A8-A07O-01A-11R-A00Z-07	2.94904928402214	Tumor
TCGA-AO-A0J2-01A-11R-A034-07	5.74215715760787	Tumor

TCGA-GM-A2DF-01A-11R-A180-07	3.68269688742323	Tumor
TCGA-E2-A1BC-01A-11R-A12P-07	4.78817409996082	Tumor
TCGA-BH-A0BJ-01A-11R-A056-07	4.26611937420224	Tumor
TCGA-AO-A0JL-01A-11R-A056-07	3.08340060855164	Tumor
TCGA-AC-A62V-01A-11R-A31O-07	4.27552113955926	Tumor
TCGA-D8-A1XG-01A-11R-A14D-07	5.17950706931944	Tumor
TCGA-B6-A400-01A-11R-A239-07	4.50949316641015	Tumor
TCGA-EW-A3U0-01A-11R-A22K-07	2.51462727287942	Tumor
TCGA-E2-A1B6-01A-31R-A12P-07	2.29431196073112	Tumor
TCGA-GM-A2DC-01A-11R-A18M-07	4.21989646206466	Tumor
TCGA-A8-A09E-01A-11R-A00Z-07	5.03086132151599	Tumor
TCGA-LL-A8F5-01A-11R-A36F-07	4.63743056467543	Tumor
TCGA-D8-A1JB-01A-11R-A13Q-07	4.85206377690963	Tumor
TCGA-E9-A3Q9-01A-11R-A21T-07	4.3717121843723	Tumor
TCGA-A7-A26H-01A-11R-A169-07	4.79630702627434	Tumor
TCGA-A7-A6VY-01A-12R-A33J-07	5.02564455560433	Tumor
TCGA-D8-A1X5-01A-11R-A14D-07	4.63198895717541	Tumor
TCGA-E9-A1ND-01A-11R-A144-07	3.80851891336443	Tumor
TCGA-AR-A1AM-01A-41R-A22K-07	5.38950830433665	Tumor
TCGA-B6-A0WW-01A-11R-A109-07	5.71643400539131	Tumor
TCGA-AR-A1AR-01A-31R-A137-07	3.78663817853233	Tumor
TCGA-EW-A2FV-01A-11R-A17B-07	4.11321707534053	Tumor

TCGA-A7-A56D-01A-11R-A27Q-07	4.73818415670556	Tumor
TCGA-BH-A1FJ-01A-11R-A13Q-07	5.21097324330959	Tumor
TCGA-XX-A899-01A-11R-A36F-07	4.83390207666916	Tumor
TCGA-A8-A090-01A-11R-A00Z-07	4.17840392242432	Tumor
TCGA-A8-A091-01A-11R-A00Z-07	5.13181992838915	Tumor
TCGA-AO-A12B-01A-11R-A10J-07	3.44720888815581	Tumor
TCGA-EW-A1PE-01A-11R-A144-07	3.77743023649873	Tumor
TCGA-GM-A5PX-01A-12R-A28M-07	3.38207234691462	Tumor
TCGA-BH-A18M-01A-11R-A12D-07	4.82957053065763	Tumor
TCGA-E2-A1LB-01A-11R-A144-07	4.83808447784863	Tumor
TCGA-E2-A10B-01A-11R-A10J-07	4.64566707774635	Tumor
TCGA-PL-A8LX-01A-11R-A41B-07	4.97654693984849	Tumor
TCGA-E9-A249-01A-11R-A169-07	4.99879123666293	Tumor
TCGA-BH-A1FR-01A-11R-A13Q-07	4.494729206	Tumor
TCGA-E9-A1QZ-01A-21R-A169-07	3.91486106825509	Tumor
TCGA-C8-A138-01A-11R-A115-07	3.98334935758198	Tumor
TCGA-AC-A2QI-01A-12R-A19W-07	5.42619765104661	Tumor
TCGA-E9-A229-01A-31R-A157-07	4.70607739446149	Tumor
TCGA-D8-A1Y0-01A-11R-A14M-07	4.95969134259704	Tumor
TCGA-AR-A0TR-01A-11R-A084-07	4.3841810805437	Tumor
TCGA-D8-A1Y2-01A-11R-A157-07	4.19467019163945	Tumor
TCGA-A2-A25F-01A-11R-A169-07	1.23223095382554	Tumor

TCGA-A7-A0DC-01A-11R-A00Z-07	2.9551453845591	Tumor
TCGA-D8-A1XB-01A-11R-A14D-07	4.12967043159117	Tumor
TCGA-D8-A1X7-01A-11R-A14M-07	5.14022240761707	Tumor
TCGA-S3-AA0Z-01A-11R-A41B-07	6.39133468151769	Tumor
TCGA-A2-A0EY-01A-11R-A034-07	4.49451161618977	Tumor
TCGA-AO-A126-01A-11R-A10J-07	4.68653414298671	Tumor
TCGA-E9-A243-01A-21R-A169-07	3.84207372176819	Tumor
TCGA-AN-A0FF-01A-11R-A034-07	4.38980081774267	Tumor
TCGA-D8-A1J9-01A-11R-A13Q-07	6.23597782499957	Tumor
TCGA-BH-A0E7-01A-11R-A034-07	4.93199973451535	Tumor
TCGA-BH-A0C1-01B-11R-A12D-07	6.01286585237344	Tumor
TCGA-D8-A1JF-01A-11R-A13Q-07	4.76230097308458	Tumor
TCGA-A8-A08Z-01A-21R-A00Z-07	3.98236389764603	Tumor
TCGA-A8-A096-01A-11R-A00Z-07	3.79273043829333	Tumor
TCGA-C8-A274-01A-11R-A16F-07	5.93315242046247	Tumor
TCGA-AC-A8OQ-01A-11R-A41B-07	2.7620936369911	Tumor
TCGA-AC-A23C-01A-12R-A169-07	4.88750577473093	Tumor
TCGA-BH-A18Q-01A-12R-A12D-07	4.71935870030414	Tumor
TCGA-AR-A0TU-01A-31R-A109-07	2.76542866194505	Tumor
TCGA-A2-A0SY-01A-31R-A084-07	4.06508110508675	Tumor
TCGA-C8-A8HR-01A-11R-A36F-07	3.65473339820762	Tumor
TCGA-BH-A5J0-01A-11R-A27Q-07	4.40088626559166	Tumor

TCGA-AR-A24R-01A-11R-A169-07	4.46440035038598	Tumor
TCGA-WT-AB41-01A-11R-A41B-07	4.58009723294623	Tumor
TCGA-AC-A3HN-01A-11R-A213-07	4.86712550314786	Tumor
TCGA-LL-A73Y-01A-11R-A33J-07	3.09570504617985	Tumor
TCGA-A8-A07L-01A-11R-A00Z-07	5.24083330086179	Tumor
TCGA-E2-A1LG-01A-21R-A14M-07	5.75644957582056	Tumor
TCGA-A2-A0CM-01A-31R-A034-07	2.42859801979762	Tumor
TCGA-S3-AA15-01A-11R-A41B-07	3.10326262660608	Tumor
TCGA-BH-A1FB-01A-11R-A13Q-07	4.93713720174522	Tumor
TCGA-BH-A0DK-01A-21R-A056-07	4.07781551901294	Tumor
TCGA-C8-A27B-01A-11R-A169-07	2.2498700468225	Tumor
TCGA-E2-A1IN-01A-11R-A13Q-07	3.55076525176158	Tumor
TCGA-E2-A156-01A-11R-A12D-07	4.49602126693142	Tumor
TCGA-A7-A0DB-01A-11R-A00Z-07	4.81035055450915	Tumor
TCGA-B6-A1KF-01A-11R-A13Q-07	4.48108577690908	Tumor
TCGA-B6-A0WY-01A-11R-A109-07	5.3248861917493	Tumor
TCGA-AO-A0JF-01A-11R-A056-07	4.1374789321476	Tumor
TCGA-AC-A2FE-01A-11R-A19W-07	5.05096307711636	Tumor
TCGA-AC-A2FO-01A-11R-A180-07	4.72698920746061	Tumor
TCGA-B6-A40C-01A-11R-A239-07	5.63898899190715	Tumor
TCGA-A2-A0CP-01A-11R-A034-07	4.11509152238593	Tumor
TCGA-A2-A3XZ-01A-42R-A239-07	5.94199598029129	Tumor

TCGA-E2-A2P6-01A-11R-A19W-07	4.77779316343224	Tumor
TCGA-E2-A14O-01A-31R-A115-07	2.62239158172908	Tumor
TCGA-C8-A26W-01A-11R-A16F-07	4.44667309528841	Tumor
TCGA-BH-A0DS-01A-11R-A056-07	4.39273643048532	Tumor
TCGA-D8-A1JS-01A-11R-A13Q-07	3.96989644729633	Tumor
TCGA-D8-A1JE-01A-11R-A13Q-07	4.80522537594566	Tumor
TCGA-LL-A7SZ-01A-32R-A352-07	2.25993529644865	Tumor
TCGA-E2-A1IU-01A-11R-A14D-07	4.96277572755066	Tumor
TCGA-A1-A0SI-01A-11R-A144-07	4.61610475617337	Tumor
TCGA-BH-A1FH-01A-12R-A13Q-07	3.78830985108215	Tumor
TCGA-D8-A27F-01A-11R-A16F-07	4.76768127918082	Tumor
TCGA-AN-A0XO-01A-11R-A109-07	5.63218677073866	Tumor
TCGA-E2-A1II-01A-11R-A144-07	2.80426011563474	Tumor
TCGA-BH-AB28-01A-31R-A41B-07	5.89263874015769	Tumor
TCGA-AR-A2LM-01A-11R-A180-07	4.87941170879425	Tumor
TCGA-BH-A0HB-01A-11R-A056-07	4.5312317243959	Tumor
TCGA-AO-A0JD-01A-11R-A056-07	5.99041261367165	Tumor
TCGA-A1-A0SG-01A-11R-A144-07	4.35921893745315	Tumor
TCGA-AC-A4ZE-01A-11R-A41B-07	3.4218292048953	Tumor
TCGA-A2-A4RW-01A-21R-A266-07	5.83743114560755	Tumor
TCGA-D8-A27H-01A-11R-A16F-07	3.97114805186031	Tumor
TCGA-AR-A0TT-01A-31R-A084-07	4.36027296384108	Tumor

TCGA-A2-A3XV-01A-21R-A239-07	0.961697405719678	Tumor
TCGA-B6-A0I5-01A-11R-A034-07	3.85642801871191	Tumor
TCGA-S3-A6ZG-01A-22R-A32P-07	3.72798578267084	Tumor
TCGA-LL-A5YL-01A-12R-A29R-07	4.85891098487911	Tumor
TCGA-AR-A2LK-01A-11R-A180-07	3.33292271095895	Tumor
TCGA-A2-A04N-01A-11R-A115-07	4.54891127958894	Tumor
TCGA-EW-A1P7-01A-21R-A144-07	3.50669079411531	Tumor
TCGA-AO-A128-01A-11R-A10J-07	4.86666075081999	Tumor
TCGA-B6-A0IQ-01A-11R-A034-07	4.27540939599834	Tumor
TCGA-E2-A155-01A-11R-A12D-07	4.98166088993899	Tumor
TCGA-E2-A574-01A-11R-A29R-07	3.84361207110538	Tumor
TCGA-AC-A3W7-01A-11R-A22K-07	5.71377553473714	Tumor
TCGA-A2-A0EQ-01A-11R-A034-07	4.53740168689247	Tumor
TCGA-A2-A0SX-01A-12R-A084-07	4.42377983839226	Tumor
TCGA-A8-A09I-01A-22R-A034-07	4.75317127765691	Tumor
TCGA-AQ-A7U7-01A-22R-A352-07	4.01117357274856	Tumor
TCGA-A7-A3IZ-01A-11R-A213-07	4.40703317443292	Tumor
TCGA-AN-A0FL-01A-11R-A034-07	4.71277734391238	Tumor
TCGA-EW-A423-01A-11R-A24H-07	4.60705802560723	Tumor
TCGA-BH-A1FC-01A-11R-A13Q-07	1.85973106652102	Tumor
TCGA-GM-A2DA-01A-11R-A18M-07	4.17974192669848	Tumor
TCGA-EW-A3E8-01B-11R-A24H-07	4.89219185224394	Tumor

TCGA-AO-A129-01A-21R-A10J-07	3.22484292057127	Tumor
TCGA-D8-A1XD-01A-11R-A14D-07	3.54046130236645	Tumor
TCGA-EW-A1IZ-01A-11R-A13Q-07	4.94748382153286	Tumor
TCGA-D8-A1JI-01A-11R-A13Q-07	3.82416702933987	Tumor
TCGA-AO-A0J5-01A-11R-A034-07	5.78284885523327	Tumor
TCGA-A2-A3XT-01A-11R-A22U-07	4.06639922891138	Tumor
TCGA-EW-A1J3-01A-11R-A13Q-07	4.27709208250431	Tumor
TCGA-D8-A1XJ-01A-11R-A14M-07	4.24006240820583	Tumor
TCGA-XX-A89A-01A-11R-A36F-07	4.73025953419096	Tumor
TCGA-B6-A0RQ-01A-11R-A115-07	4.88749115255003	Tumor
TCGA-A2-A1FV-01A-11R-A13Q-07	3.94527058551635	Tumor
TCGA-BH-A0H5-01A-21R-A115-07	3.66789212523023	Tumor
TCGA-AN-A0FD-01A-11R-A034-07	4.09098042266464	Tumor
TCGA-A8-A09R-01A-11R-A00Z-07	5.10188955300621	Tumor
TCGA-AR-A24P-01A-11R-A169-07	4.86516659637744	Tumor
TCGA-AN-A0XP-01A-11R-A109-07	4.20881375903889	Tumor
TCGA-AR-A256-01A-11R-A169-07	4.22970327221029	Tumor
TCGA-EW-A2FS-01A-11R-A17B-07	3.75845242843128	Tumor
TCGA-A2-A0CS-01A-11R-A115-07	4.18002848052637	Tumor
TCGA-D8-A27N-01A-11R-A16F-07	2.60772089829931	Tumor
TCGA-BH-A0B8-01A-21R-A056-07	3.7218319737045	Tumor
TCGA-A8-A08B-01A-11R-A00Z-07	4.27297869537357	Tumor

TCGA-E2-A159-01A-11R-A115-07	5.76744558169461	Tumor
TCGA-AN-A0FY-01A-11R-A034-07	4.25410268119416	Tumor
TCGA-AR-A0TY-01A-12R-A115-07	4.60378061980584	Tumor
TCGA-AR-A2LE-01A-11R-A180-07	4.65827433146381	Tumor
TCGA-OL-A6VR-01A-32R-A33J-07	5.53485222772357	Tumor
TCGA-BH-A0HI-01A-11R-A084-07	4.75478597560684	Tumor
TCGA-BH-A208-01A-11R-A157-07	4.57207716599095	Tumor
TCGA-EW-A1P6-01A-11R-A144-07	4.23981807903362	Tumor
TCGA-E2-A14W-01A-11R-A12D-07	4.71259578044703	Tumor
TCGA-S3-A6ZH-01A-22R-A32P-07	4.85548645969071	Tumor
TCGA-E2-A1L8-01A-11R-A13Q-07	4.86912618312587	Tumor
TCGA-EW-A1IX-01A-12R-A144-07	4.93515326042343	Tumor
TCGA-AR-A0U0-01A-11R-A109-07	5.04639065127169	Tumor
TCGA-AR-A255-01A-11R-A169-07	4.56741158814907	Tumor
TCGA-E2-A2P5-01A-11R-A19W-07	4.28722847619509	Tumor
TCGA-EW-A1PC-01B-11R-A21T-07	3.3143629998107	Tumor
TCGA-AC-A3OD-01A-11R-A21T-07	3.75034924058415	Tumor
TCGA-A8-A08X-01A-21R-A00Z-07	5.03950691856042	Tumor
TCGA-A1-A0SH-01A-11R-A084-07	5.17631080332886	Tumor
TCGA-PL-A8LY-01A-11R-A41B-07	4.85781184165497	Tumor
TCGA-E2-A158-01A-11R-A12D-07	4.13180346931973	Tumor
TCGA-AC-A23G-01A-11R-A213-07	3.85480356958655	Tumor

TCGA-AR-A5QM-01A-11R-A27Q-07	4.67138396273252	Tumor
TCGA-GM-A2DL-01A-11R-A18M-07	3.61136126391975	Tumor
TCGA-A2-A3XU-01A-12R-A22U-07	4.62284252184413	Tumor
TCGA-A7-A425-01A-11R-A24H-07	5.15375265243303	Tumor
TCGA-C8-A12Q-01A-11R-A115-07	5.5677796718778	Tumor
TCGA-E9-A1N9-01A-11R-A14D-07	3.67481088744553	Tumor
TCGA-A7-A4SB-01A-21R-A266-07	5.19364560061083	Tumor
TCGA-A8-A0A7-01A-11R-A00Z-07	3.27045418940571	Tumor
TCGA-BH-A0DQ-01A-11R-A084-07	4.1049236837009	Tumor
TCGA-B6-A408-01A-12R-A24H-07	3.9725731902332	Tumor
TCGA-AO-A12A-01A-21R-A115-07	4.79180365455353	Tumor
TCGA-BH-A5IZ-01A-11R-A27Q-07	4.94717520165699	Tumor
TCGA-C8-A12U-01A-11R-A115-07	4.6133784173658	Tumor
TCGA-A8-A081-01A-11R-A00Z-07	5.72335104309778	Tumor
TCGA-AR-A1AQ-01A-11R-A12P-07	5.06095526928323	Tumor
TCGA-AR-A24Z-01A-11R-A169-07	3.88128285167182	Tumor
TCGA-D8-A27M-01A-11R-A16F-07	4.90605358972963	Tumor
TCGA-A2-A0YF-01A-21R-A109-07	4.91558782342299	Tumor
TCGA-A7-A26I-01B-06R-A22O-07	4.18896263702316	Tumor
TCGA-AR-A250-01A-31R-A169-07	3.4725137657338	Tumor
TCGA-E2-A1IG-01A-11R-A144-07	3.95335837319653	Tumor
TCGA-OL-A5RW-01A-11R-A28M-07	5.35562242973081	Tumor

TCGA-AN-A03Y-01A-21R-A00Z-07	3.64186967436221	Tumor
TCGA-E2-A1B0-01A-11R-A12P-07	4.78513724777074	Tumor
TCGA-C8-A1HJ-01A-11R-A13Q-07	3.03506079690511	Tumor
TCGA-C8-A12P-01A-11R-A115-07	4.85694088489266	Tumor
TCGA-LL-A740-01A-21R-A32P-07	4.50176395383338	Tumor
TCGA-EW-A1OV-01A-11R-A144-07	5.41128047735045	Tumor
TCGA-E9-A1NH-01A-11R-A14D-07	4.51657622607813	Tumor
TCGA-A7-A26I-01A-11R-A169-07	3.81454016972927	Tumor
TCGA-E2-A1IJ-01A-11R-A144-07	4.10873352786627	Tumor
TCGA-BH-A1F6-01A-11R-A13Q-07	2.46042804557587	Tumor
TCGA-AR-A2LN-01A-21R-A18M-07	4.13129314500955	Tumor
TCGA-A8-A0AD-01A-11R-A056-07	4.87774915706665	Tumor
TCGA-BH-A203-01A-12R-A169-07	4.64220480187386	Tumor
TCGA-C8-A1HO-01A-11R-A13Q-07	4.9125588553674	Tumor
TCGA-A2-A0YL-01A-21R-A109-07	4.7149158715959	Tumor
TCGA-A2-A04T-01A-21R-A034-07	4.90605358972963	Tumor
TCGA-B6-A0IC-01A-11R-A034-07	5.31769117625725	Tumor
TCGA-A2-A04Q-01A-21R-A034-07	3.5862603423168	Tumor
TCGA-E2-A1IL-01A-11R-A14D-07	5.13295103943028	Tumor
TCGA-LL-A5YO-01A-21R-A28M-07	4.99502766849728	Tumor
TCGA-A2-A25A-01A-12R-A16F-07	4.45909057768484	Tumor
TCGA-A7-A0CE-01A-11R-A00Z-07	3.27738969881086	Tumor

TCGA-EW-A2FR-01A-11R-A21T-07	4.59987119678075	Tumor
TCGA-A2-A1FX-01A-11R-A13Q-07	4.26933991102072	Tumor
TCGA-E2-A14Y-01A-21R-A12D-07	5.79975046170089	Tumor
TCGA-BH-A0H6-01A-21R-A056-07	4.20504055507438	Tumor
TCGA-E2-A105-01A-11R-A10J-07	3.47019843955567	Tumor
TCGA-LD-A7W5-01A-22R-A352-07	4.85094940441723	Tumor
TCGA-A2-A0T4-01A-31R-A084-07	4.89277472205113	Tumor
TCGA-E9-A1R4-01A-21R-A14D-07	5.72565936875253	Tumor
TCGA-BH-A0B1-01A-12R-A056-07	4.00710577877344	Tumor
TCGA-C8-A12N-01A-11R-A115-07	4.4440043985831	Tumor
TCGA-A2-A25E-01A-11R-A169-07	4.6083006621683	Tumor
TCGA-BH-A0BQ-01A-21R-A115-07	5.22866864240899	Tumor
TCGA-C8-A133-01A-32R-A12D-07	5.6722018483706	Tumor
TCGA-A8-A07C-01A-11R-A034-07	1.8413693545154	Tumor
TCGA-GM-A3NY-01A-11R-A21T-07	3.50707152610811	Tumor
TCGA-B6-A0IA-01A-11R-A034-07	4.91053600456168	Tumor
TCGA-C8-A1HG-01A-11R-A137-07	3.47047178942371	Tumor
TCGA-A1-A0SN-01A-11R-A144-07	4.91542529155076	Tumor
TCGA-A8-A09T-01A-11R-A00Z-07	4.4750589349629	Tumor
TCGA-AO-A03L-01A-41R-A056-07	3.62733835047881	Tumor
TCGA-V7-A7HQ-01A-11R-A33J-07	3.96346487590011	Tumor
TCGA-BH-A0BO-01A-23R-A12D-07	4.05828185663859	Tumor

TCGA-B6-A0RN-01A-12R-A084-07	4.58758701926048	Tumor
TCGA-AR-A1AL-01A-21R-A12P-07	4.15193997415627	Tumor
TCGA-BH-A1F0-01A-11R-A137-07	3.39870607451041	Tumor
TCGA-E9-A1R7-01A-11R-A14M-07	4.73105830398657	Tumor
TCGA-E2-A15J-01A-11R-A12P-07	5.29074876734685	Tumor
TCGA-B6-A1KN-01A-11R-A13Q-07	4.9540985761221	Tumor
TCGA-BH-A0DI-01A-21R-A12P-07	5.13825746275896	Tumor
TCGA-AR-A24U-01A-11R-A169-07	3.91896815673509	Tumor
TCGA-A8-A0AB-01A-11R-A034-07	4.89541411563362	Tumor
TCGA-E2-A15G-01A-11R-A12D-07	5.10476437226216	Tumor
TCGA-BH-A0HK-01A-11R-A056-07	3.04722427001751	Tumor
TCGA-A8-A06Z-01A-11R-A00Z-07	5.29554671702105	Tumor
TCGA-E2-A1LL-01A-11R-A144-07	5.04199200074637	Tumor
TCGA-E2-A1L6-01A-11R-A13Q-07	4.83682295284468	Tumor
TCGA-A2-A1G0-01A-11R-A13Q-07	4.02885200584749	Tumor
TCGA-BH-A1FL-01A-11R-A13Q-07	4.38692817792175	Tumor
TCGA-B6-A0IN-01A-11R-A034-07	4.5371220882067	Tumor
TCGA-B6-A0IP-01A-11R-A034-07	4.46634651434283	Tumor
TCGA-B6-A0IB-01A-11R-A034-07	4.61739840529181	Tumor
TCGA-B6-A1KI-01A-11R-A14M-07	4.53705994780599	Tumor
TCGA-E2-A14U-01A-11R-A22K-07	4.98038638593563	Tumor
TCGA-BH-A0E0-01A-11R-A056-07	4.56733856864401	Tumor

TCGA-E2-A154-01A-11R-A115-07	4.54573366503401	Tumor
TCGA-BH-A0EA-01A-11R-A115-07	5.18586654531133	Tumor
TCGA-A2-A04U-01A-11R-A115-07	2.40034665051597	Tumor
TCGA-Z7-A8R5-01A-42R-A41B-07	4.88462722390928	Tumor
TCGA-BH-A18U-01A-21R-A12D-07	4.40544103595723	Tumor
TCGA-BH-A0HW-01A-11R-A034-07	4.05397148245987	Tumor
TCGA-D8-A27T-01A-11R-A16F-07	3.7578766597479	Tumor
TCGA-A2-A0ST-01A-12R-A084-07	2.75381844317354	Tumor
TCGA-B6-A1KC-01B-11R-A157-07	4.335447525	Tumor
TCGA-BH-A0HF-01A-11R-A056-07	4.44311594563843	Tumor
TCGA-LL-A6FR-01A-12R-A31O-07	3.35106289894039	Tumor
TCGA-AN-A0FX-01A-11R-A034-07	3.98611053214055	Tumor
TCGA-D8-A13Y-01A-11R-A115-07	2.87125225885525	Tumor
TCGA-A2-A3Y0-01A-11R-A239-07	5.03891460085965	Tumor
TCGA-B6-A0WS-01A-11R-A115-07	4.98377344561616	Tumor
TCGA-B6-A0RM-01A-11R-A084-07	4.97556599170945	Tumor
TCGA-E9-A54Y-01A-11R-A466-07	2.25701061820602	Tumor
TCGA-B6-A0X1-01A-11R-A109-07	5.60404759493687	Tumor
TCGA-A7-A0D9-01A-31R-A056-07	5.5191109690597	Tumor
TCGA-BH-A0B3-01A-11R-A056-07	4.70610503012691	Tumor
TCGA-AC-A3W6-01A-12R-A22K-07	4.62823117053257	Tumor
TCGA-E2-A15K-01A-11R-A12P-07	4.43082718555768	Tumor

TCGA-GM-A2D9-01A-11R-A18M-07	4.24101642272041	Tumor
TCGA-5L-AAT1-01A-12R-A41B-07	4.55006336316472	Tumor
TCGA-E9-A22H-01A-11R-A157-07	4.33806779754262	Tumor
TCGA-E2-A15E-06A-11R-A12D-07	4.00647756407121	Tumor
TCGA-AO-A03M-01B-11R-A10J-07	3.68713909354851	Tumor
TCGA-BH-A0H9-01A-11R-A056-07	4.92473650779137	Tumor
TCGA-A7-A0CD-01A-11R-A00Z-07	3.36857281085828	Tumor
TCGA-E2-A15E-01A-11R-A12D-07	4.86266463059071	Tumor
TCGA-E2-A15S-01A-11R-A115-07	4.64962695459563	Tumor
TCGA-S3-AA11-01A-31R-A41B-07	5.24818186093661	Tumor
TCGA-D8-A1XS-01A-11R-A14M-07	3.71621173843209	Tumor
TCGA-E2-A1BD-01A-11R-A12P-07	4.44079277540492	Tumor
TCGA-AN-A0FS-01A-11R-A034-07	3.83175566064403	Tumor
TCGA-BH-A0DG-01A-21R-A12P-07	4.30526285797413	Tumor
TCGA-A2-A0YT-01A-11R-A109-07	5.65887125771466	Tumor
TCGA-A2-A0T2-01A-11R-A084-07	3.94659051332947	Tumor
TCGA-S3-AA10-01A-21R-A41B-07	1.55586522548701	Tumor
TCGA-E2-A1LK-01A-21R-A14D-07	3.29113201200875	Tumor
TCGA-B6-A0IH-01A-11R-A115-07	4.50033679810706	Tumor
TCGA-A8-A0A4-01A-11R-A00Z-07	4.01092302718479	Tumor
TCGA-D8-A143-01A-11R-A115-07	3.87434451614564	Tumor
TCGA-D8-A140-01A-11R-A115-07	3.58018167197222	Tumor

TCGA-A8-A076-01A-21R-A00Z-07	4.6897520285906	Tumor
TCGA-BH-A8FZ-01A-11R-A352-07	4.90699638936579	Tumor
TCGA-E9-A1RE-01A-11R-A157-07	3.33726865489392	Tumor
TCGA-A8-A08S-01A-11R-A034-07	4.16954824854787	Tumor
TCGA-A2-A0T6-01A-11R-A084-07	5.00492829488917	Tumor
TCGA-AC-A62Y-01A-11R-A29R-07	5.35947900218544	Tumor
TCGA-AC-A3BB-01A-21R-A19W-07	4.41856777929971	Tumor
TCGA-AR-A24S-01A-11R-A169-07	4.54665373346671	Tumor
TCGA-BH-A0W4-01A-11R-A109-07	4.80976941467757	Tumor
TCGA-EW-A6SA-01A-21R-A32P-07	4.11529964402555	Tumor
TCGA-E9-A1N8-01A-11R-A144-07	3.41754200675105	Tumor
TCGA-PE-A5DD-01A-12R-A27Q-07	5.2538153459684	Tumor
TCGA-BH-A18L-01A-32R-A12D-07	3.83351757654728	Tumor
TCGA-E9-A5FK-01A-11R-A27Q-07	3.6058261676978	Tumor
TCGA-A7-A0CH-01A-21R-A00Z-07	4.71958322084123	Tumor
TCGA-AR-A24T-01A-11R-A169-07	5.05820824610355	Tumor
TCGA-A8-A07B-01A-11R-A00Z-07	4.37579013269661	Tumor
TCGA-AO-A1KR-01A-12R-A144-07	5.62941199519015	Tumor
TCGA-AO-A1KS-01A-11R-A13Q-07	4.82261799061361	Tumor
TCGA-BH-A2L8-01A-11R-A18M-07	4.2298878121807	Tumor
TCGA-LD-A7W6-01A-81R-A352-07	4.62376153471654	Tumor
TCGA-A8-A09Q-01A-11R-A00Z-07	3.57332604797551	Tumor

TCGA-B6-A0RO-01A-22R-A084-07	5.54834105839783	Tumor
TCGA-HN-A2NL-01A-11R-A18M-07	0.917469946679193	Tumor
TCGA-D8-A27R-01A-11R-A16F-07	5.36563655260175	Tumor
TCGA-BH-A42T-01A-11R-A24H-07	5.6655945841403	Tumor
TCGA-AR-A24O-01A-11R-A169-07	4.14552258131369	Tumor
TCGA-BH-A0DP-01A-21R-A056-07	3.97455684456928	Tumor
TCGA-AO-A03O-01A-11R-A00Z-07	4.47218230368947	Tumor
TCGA-A1-A0SP-01A-11R-A084-07	2.66805102859978	Tumor
TCGA-AR-A0TP-01A-11R-A084-07	4.09988428273055	Tumor
TCGA-E9-A1NF-01A-11R-A14D-07	3.99605424829619	Tumor
TCGA-AR-A24M-01A-11R-A169-07	4.15107135264932	Tumor
TCGA-A2-A0EX-01A-21R-A034-07	4.29698593163197	Tumor
TCGA-D8-A27V-01A-12R-A17B-07	4.98722535017016	Tumor
TCGA-AN-A0XV-01A-11R-A109-07	4.33778957488694	Tumor
TCGA-A8-A083-01A-21R-A00Z-07	4.93807873664229	Tumor
TCGA-A2-A0CV-01A-31R-A115-07	4.93064746195763	Tumor
TCGA-A2-A4S2-01A-12R-A266-07	5.11191602760414	Tumor
TCGA-AO-A124-01A-11R-A10J-07	5.20912969773405	Tumor
TCGA-E9-A1NA-01A-11R-A144-07	5.36066275851384	Tumor
TCGA-A8-A09A-01A-11R-A00Z-07	3.83265713932914	Tumor
TCGA-E9-A2JT-01A-22R-A18M-07	4.01871428207887	Tumor
TCGA-OL-A5RU-01A-11R-A28M-07	5.23571174452279	Tumor

TCGA-A2-A0D0-01A-11R-A00Z-07	4.26446887345803	Tumor
TCGA-E2-A15R-01A-11R-A115-07	5.11836803255651	Tumor
TCGA-OL-A6VO-01A-12R-A33J-07	2.07296327155522	Tumor
TCGA-AR-A1AU-01A-11R-A12P-07	5.48288699529614	Tumor
TCGA-AO-A0J3-01A-11R-A034-07	3.81614911394902	Tumor
TCGA-D8-A1XQ-01A-11R-A14M-07	1.76290154793598	Tumor
TCGA-AR-A24V-01A-21R-A169-07	3.16203275056206	Tumor
TCGA-E9-A1NE-01A-21R-A14M-07	4.18676200658217	Tumor
TCGA-AO-A1KP-01A-11R-A13Q-07	4.67427985137945	Tumor
TCGA-E9-A1RH-01A-21R-A169-07	5.42327897905929	Tumor
TCGA-BH-A18S-01A-11R-A12D-07	4.91284144514327	Tumor
TCGA-BH-A0BA-01A-11R-A056-07	4.30117058015138	Tumor
TCGA-E9-A1R6-01A-11R-A14D-07	4.60048980481865	Tumor
TCGA-OL-A5S0-01A-11R-A28M-07	2.923909795	Tumor
TCGA-BH-A1EO-01A-11R-A137-07	5.258647058	Tumor
TCGA-B6-A0RP-01A-21R-A084-07	3.73862734280201	Tumor
TCGA-UL-AAZ6-01A-11R-A41B-07	5.35466372529326	Tumor
TCGA-B6-A0RT-01A-21R-A084-07	3.32213005805599	Tumor
TCGA-BH-A0AZ-01A-21R-A12P-07	4.27202318906105	Tumor
TCGA-BH-A1ES-06A-12R-A24H-07	5.97727534350839	Tumor
TCGA-AR-A252-01A-11R-A169-07	4.30221667665993	Tumor
TCGA-AQ-A54N-01A-11R-A266-07	4.35160712947052	Tumor

TCGA-A7-A5ZW-01A-12R-A29R-07	4.03511359807046	Tumor
TCGA-BH-A209-01A-11R-A157-07	4.47322192940515	Tumor
TCGA-3C-AALI-01A-11R-A41B-07	4.82579071453284	Tumor
TCGA-E2-A1L9-01A-11R-A13Q-07	4.06897169899089	Tumor
TCGA-BH-A0B9-01A-11R-A056-07	3.77633564312982	Tumor
TCGA-GM-A2DH-01A-11R-A180-07	4.25066603325051	Tumor
TCGA-A2-A0CK-01A-11R-A22K-07	4.50399106216937	Tumor
TCGA-A8-A07F-01A-11R-A00Z-07	3.79467578798686	Tumor
TCGA-AR-A254-01A-21R-A169-07	3.99206140947953	Tumor
TCGA-B6-A0IM-01A-11R-A034-07	4.95857823423318	Tumor
TCGA-B6-A0X7-01A-11R-A10J-07	5.2241522084355	Tumor
TCGA-EW-A1OX-01A-11R-A144-07	5.18017571521789	Tumor
TCGA-GM-A4E0-01A-12R-A266-07	3.62096169693143	Tumor
TCGA-E2-A14X-01A-11R-A115-07	4.78918148659354	Tumor
TCGA-B6-A0I1-01A-11R-A21T-07	4.15030783282561	Tumor
TCGA-A2-A0YM-01A-11R-A109-07	3.5741499605097	Tumor
TCGA-A8-A08R-01A-11R-A034-07	1.80661277375351	Tumor
TCGA-E9-A1N4-01A-11R-A14M-07	3.97294992699427	Tumor
TCGA-D8-A1Y3-01A-11R-A157-07	3.66166980252421	Tumor
TCGA-LL-A441-01A-11R-A24H-07	2.55949181320504	Tumor
TCGA-D8-A1XT-01A-11R-A14M-07	3.80204842307698	Tumor
TCGA-A8-A06U-01A-11R-A00Z-07	4.04480501033689	Tumor

TCGA-A2-A0EP-01A-52R-A22U-07	3.99557496714191	Tumor
TCGA-D8-A27G-01A-11R-A16F-07	5.46335761587859	Tumor
TCGA-A7-A26J-01A-11R-A169-07	4.15882172229711	Tumor
TCGA-E2-A1B5-01A-21R-A12P-07	3.67774500748467	Tumor
TCGA-OL-A5D8-01A-11R-A27Q-07	5.1865758281045	Tumor
TCGA-A8-A06X-01A-21R-A00Z-07	5.51927455098288	Tumor
TCGA-E2-A1LE-01A-12R-A19W-07	4.75079942292261	Tumor
TCGA-A2-A04X-01A-21R-A034-07	3.99535788178005	Tumor
TCGA-AR-A2LL-01A-11R-A180-07	4.91893000508683	Tumor
TCGA-A8-A0A9-01A-11R-A00Z-07	4.18793398305576	Tumor
TCGA-BH-A1EX-01A-11R-A13Q-07	5.02082663573289	Tumor
TCGA-BH-A0AV-01A-31R-A115-07	3.4381065136182	Tumor
TCGA-BH-A1FM-01A-11R-A13Q-07	3.64468694288434	Tumor
TCGA-AQ-A1H3-01A-31R-A13Q-07	4.60966041811521	Tumor
TCGA-OL-A5RY-01A-21R-A28M-07	5.00404289292381	Tumor
TCGA-E9-A245-01A-22R-A16F-07	5.94382992484954	Tumor
TCGA-C8-A12O-01A-11R-A115-07	3.86289767001989	Tumor
TCGA-E2-A1AZ-01A-11R-A12P-07	3.55112222195786	Tumor
TCGA-BH-A0HY-01A-11R-A056-07	5.09252459990213	Tumor
TCGA-EW-A1PF-01A-11R-A144-07	6.0782639767829	Tumor
TCGA-BH-A0BV-01A-11R-A00Z-07	2.83155300362634	Tumor
TCGA-PE-A5DE-01A-11R-A27Q-07	4.74310021709134	Tumor

TCGA-AR-A251-01A-12R-A169-07	3.59769801902125	Tumor
TCGA-E2-A1B1-01A-21R-A12P-07	4.38054937740122	Tumor
TCGA-BH-A0E2-01A-11R-A056-07	4.2181146778013	Tumor
TCGA-AR-A0TW-01A-11R-A084-07	4.05262456204547	Tumor
TCGA-A7-A13E-01A-11R-A12P-07	3.09703764971599	Tumor
TCGA-C8-A1HM-01A-12R-A137-07	3.52219388361189	Tumor
TCGA-E2-A1LS-01A-12R-A157-07	3.85806058989965	Tumor
TCGA-A2-A0YK-01A-22R-A109-07	4.28618624001597	Tumor
TCGA-LL-A50Y-01A-11R-A266-07	4.34914585575654	Tumor
TCGA-B6-A0RS-01A-11R-A084-07	4.45225277370696	Tumor
TCGA-PL-A8LZ-01A-31R-A36F-07	2.85618895081904	Tumor
TCGA-A7-A2KD-01A-31R-A21T-07	3.59109293363433	Tumor
TCGA-LD-A9QF-01A-32R-A41B-07	4.86262000172458	Tumor
TCGA-AR-A1AK-01A-21R-A12P-07	3.73862734280201	Tumor
TCGA-LL-A6FQ-01A-11R-A31O-07	3.88962929067343	Tumor
TCGA-E9-A1RB-01A-11R-A157-07	4.90336611809544	Tumor
TCGA-AN-A0AJ-01A-11R-A00Z-07	4.27867618281327	Tumor
TCGA-A2-A0D2-01A-21R-A034-07	2.94086208408379	Tumor
TCGA-EW-A6S9-01A-22R-A33J-07	4.13857694846446	Tumor
TCGA-A8-A093-01A-11R-A00Z-07	3.72365684923197	Tumor
TCGA-AC-A2FB-01A-11R-A17B-07	4.59796612202755	Tumor
TCGA-C8-A137-01A-11R-A115-07	5.37504638407736	Tumor

TCGA-E9-A1N3-01A-12R-A157-07	5.62090013318728	Tumor
TCGA-AO-A03U-01B-21R-A10J-07	6.38042296791271	Tumor
TCGA-A2-A0D1-01A-11R-A034-07	4.47932758280766	Tumor
TCGA-E2-A572-01A-13R-A31O-07	4.55084527000313	Tumor
TCGA-B6-A0RE-01A-11R-A056-07	4.66505161428921	Tumor
TCGA-AR-A5QN-01A-12R-A28M-07	4.94254734680377	Tumor
TCGA-D8-A1JC-01A-11R-A13Q-07	5.60648664539861	Tumor
TCGA-A2-A0T3-01A-21R-A115-07	2.86896326854108	Tumor
TCGA-B6-A0I6-01A-11R-A034-07	4.23000313767638	Tumor
TCGA-B6-A0IK-01A-12R-A056-07	5.91928525326739	Tumor
TCGA-B6-A0IO-01A-11R-A034-07	4.41056498178899	Tumor
TCGA-B6-A0WZ-01A-11R-A109-07	5.64392543747476	Tumor
TCGA-BH-A204-01A-11R-A157-07	4.44020807501375	Tumor
TCGA-A1-A0SQ-01A-21R-A144-07	4.46188866443282	Tumor
TCGA-AO-A0JC-01A-11R-A056-07	2.09589067235141	Tumor
TCGA-BH-A0DX-01A-11R-A115-07	5.02162185785152	Tumor
TCGA-A2-A0T1-01A-21R-A084-07	4.75141016006437	Tumor
TCGA-AO-A0J8-01A-21R-A034-07	5.00480697750986	Tumor
TCGA-D8-A146-01A-31R-A115-07	4.77894445758767	Tumor
TCGA-AN-A0AT-01A-11R-A034-07	2.62119620141674	Tumor
TCGA-BH-A1EY-01A-11R-A13Q-07	4.97935773936525	Tumor
TCGA-A8-A08F-01A-11R-A00Z-07	4.39673491753557	Tumor

TCGA-GM-A3XN-01A-12R-A22U-07	4.1917047655854	Tumor
TCGA-E2-A15K-06A-11R-A12P-07	4.46608543677757	Tumor
TCGA-E9-A1R5-01A-11R-A14M-07	5.78547470102129	Tumor
TCGA-BH-A0B7-01A-12R-A115-07	5.26741594303495	Tumor
TCGA-AO-A1KT-01A-11R-A13Q-07	4.49859554109593	Tumor
TCGA-AR-A1AJ-01A-21R-A12P-07	4.72161873885594	Tumor
TCGA-OL-A5RV-01A-12R-A28M-07	5.46983063718132	Tumor
TCGA-EW-A1P3-01A-11R-A144-07	4.12627082490916	Tumor
TCGA-BH-A0EI-01A-11R-A115-07	4.88310757127804	Tumor
TCGA-BH-A0H7-01A-13R-A056-07	4.15587063929305	Tumor
TCGA-BH-A0GZ-01A-11R-A056-07	4.13299626543126	Tumor
TCGA-D8-A1XZ-01A-11R-A14M-07	4.69347580619021	Tumor
TCGA-A8-A06Q-01A-11R-A034-07	5.67453127047726	Tumor
TCGA-OL-A66K-01A-11R-A29R-07	4.93290684329073	Tumor
TCGA-D8-A1JN-01A-11R-A13Q-07	5.44617681329785	Tumor
TCGA-D8-A1XL-01A-11R-A14M-07	4.93577559743654	Tumor
TCGA-E2-A3DX-01A-21R-A213-07	4.25958886587835	Tumor
TCGA-S3-AA12-01A-11R-A41B-07	5.60643926693185	Tumor
TCGA-A8-A095-01A-11R-A00Z-07	4.94622554771203	Tumor
TCGA-A2-A0EM-01A-11R-A034-07	4.43156279045411	Tumor
TCGA-AO-A03P-01A-11R-A00Z-07	3.66116811849074	Tumor
TCGA-A2-A0CW-01A-21R-A115-07	5.31656936312463	Tumor

TCGA-BH-A0BC-01A-22R-A084-07	4.67066474594765	Tumor
TCGA-A2-A0CU-01A-12R-A034-07	5.07556685840169	Tumor
TCGA-OL-A66I-01A-21R-A29R-07	2.71979675673579	Tumor
TCGA-A7-A13H-01A-11R-A22K-07	5.18387949012707	Tumor
TCGA-E2-A15L-01A-11R-A12D-07	4.84037762638834	Tumor
TCGA-AQ-A54O-01A-11R-A266-07	3.64726845310438	Tumor
TCGA-A7-A26E-01B-06R-A277-07	4.00513945266128	Tumor
TCGA-A2-A0CT-01A-31R-A056-07	3.94170730868097	Tumor
TCGA-AO-A0JE-01A-11R-A056-07	3.87094661252511	Tumor
TCGA-B6-A0IE-01A-11R-A034-07	2.793583802	Tumor
TCGA-A8-A092-01A-11R-A00Z-07	4.19519796607709	Tumor
TCGA-LD-A66U-01A-11R-A31O-07	4.11169907219742	Tumor
TCGA-UU-A93S-01A-21R-A41B-07	5.53176508840379	Tumor
TCGA-BH-A0BR-01A-21R-A115-07	6.14594640716614	Tumor
TCGA-C8-A12Z-01A-11R-A115-07	5.23015304703992	Tumor
TCGA-BH-A1FE-01A-11R-A13Q-07	5.24795788565106	Tumor
TCGA-D8-A145-01A-11R-A115-07	4.37316792899759	Tumor
TCGA-EW-A1PD-01A-11R-A144-07	4.73346823309504	Tumor
TCGA-E2-A150-01A-11R-A12D-07	5.68279238159431	Tumor
TCGA-D8-A1JD-01A-11R-A13Q-07	4.89785997361841	Tumor
TCGA-A8-A06Y-01A-21R-A00Z-07	5.10625199143465	Tumor
TCGA-B6-A409-01A-11R-A24H-07	2.34471489449013	Tumor

TCGA-E9-A1RA-01A-11R-A14D-07	4.59746562277446	Tumor
TCGA-E9-A248-01A-11R-A169-07	3.9566700392189	Tumor
TCGA-BH-A18P-01A-11R-A12D-07	4.45973324152274	Tumor
TCGA-LQ-A4E4-01A-11R-A266-07	5.81330149552516	Tumor
TCGA-E2-A15M-01A-11R-A12D-07	3.457948816	Tumor
TCGA-A8-A07U-01A-11R-A034-07	2.70112760867665	Tumor
TCGA-C8-A1HK-01A-21R-A13Q-07	3.77146305836565	Tumor
TCGA-C8-A273-01A-11R-A16F-07	5.29098462226363	Tumor
TCGA-BH-A8FY-01A-11R-A36F-07	4.51535922744661	Tumor
TCGA-BH-A0B2-01A-11R-A10J-07	3.78547731662967	Tumor
TCGA-AC-A62X-01A-11R-A29R-07	5.05855028676734	Tumor
TCGA-BH-A0E1-01A-11R-A056-07	5.05280712169826	Tumor
TCGA-A1-A0SO-01A-22R-A084-07	2.28711763578818	Tumor
TCGA-A7-A0CG-01A-12R-A056-07	5.20988221122127	Tumor
TCGA-C8-A278-01A-11R-A169-07	4.47995483169067	Tumor
TCGA-D8-A27E-01A-11R-A16F-07	5.32649420470058	Tumor
TCGA-AR-A24H-01A-11R-A169-07	4.30319624698775	Tumor
TCGA-C8-A12K-01A-21R-A115-07	3.63434931387689	Tumor
TCGA-A8-A09K-01A-11R-A00Z-07	4.9687082592831	Tumor
TCGA-BH-A18V-06A-11R-A213-07	5.41473089400469	Tumor
TCGA-E2-A9RU-01A-11R-A41B-07	4.8743789466582	Tumor
TCGA-E2-A15A-01A-11R-A12D-07	4.96991025768207	Tumor

TCGA-AR-A0U4-01A-11R-A109-07	2.60492529819924	Tumor
TCGA-A2-A0EV-01A-11R-A034-07	4.70124961952071	Tumor
TCGA-D8-A1XF-01A-11R-A14D-07	4.52389436487732	Tumor
TCGA-LL-A5YP-01A-21R-A28M-07	3.20083388455689	Tumor
TCGA-E2-A1IO-01A-11R-A144-07	4.33826751405771	Tumor
TCGA-D8-A3Z5-01A-41R-A24H-07	4.54468938679497	Tumor
TCGA-D8-A1XR-01A-11R-A14M-07	3.8694766339654	Tumor
TCGA-A1-A0SM-01A-11R-A084-07	5.41208032709115	Tumor
TCGA-D8-A1JP-01A-11R-A13Q-07	4.43082718555768	Tumor
TCGA-AC-A6IX-01A-12R-A32P-07	5.87017234741952	Tumor
TCGA-D8-A4Z1-01A-21R-A266-07	5.15797339892551	Tumor
TCGA-B6-A402-01A-11R-A239-07	2.79460301464876	Tumor
TCGA-A2-A1FZ-01A-51R-A14D-07	5.84620244092796	Tumor
TCGA-E9-A3HO-01A-11R-A213-07	3.54119266272144	Tumor
TCGA-AC-A2BM-01A-11R-A21T-07	4.57677002314756	Tumor
TCGA-BH-A0E9-01B-11R-A115-07	5.21763399394859	Tumor
TCGA-BH-A0C3-01A-21R-A12P-07	3.91745085116102	Tumor
TCGA-B6-A40B-01A-11R-A239-07	5.36546158673608	Tumor
TCGA-BH-A0HO-01A-11R-A034-07	5.34791718483461	Tumor
TCGA-AR-A1AS-01A-11R-A12P-07	5.47054337249803	Tumor
TCGA-E9-A1RG-01A-11R-A14D-07	3.41107366438429	Tumor
TCGA-D8-A27K-01A-11R-A16F-07	4.78726005800936	Tumor

TCGA-E9-A1R2-01A-11R-A14D-07	4.86901263853985	Tumor
TCGA-A7-A13D-01B-04R-A277-07	4.26233543112676	Tumor
TCGA-A7-A13D-01A-13R-A277-07	4.24981696544229	Tumor
TCGA-C8-A26V-01A-11R-A16F-07	5.31823729204689	Tumor
TCGA-BH-A1ET-01A-11R-A137-07	4.80288078941674	Tumor
TCGA-AO-A0JA-01A-11R-A056-07	5.1935391590556	Tumor
TCGA-5L-AAT0-01A-12R-A41B-07	4.66627940122911	Tumor
TCGA-AC-A6NO-01A-12R-A33J-07	4.94511604692257	Tumor
TCGA-C8-A3M7-01A-12R-A21T-07	4.51465251887501	Tumor
TCGA-A2-A0ET-01A-31R-A034-07	1.63384965910469	Tumor
TCGA-A7-A6VW-01A-21R-A33J-07	5.02578184778694	Tumor
TCGA-AR-A0TS-01A-11R-A115-07	4.06066134981892	Tumor
TCGA-A2-A0SW-01A-11R-A084-07	3.88806130781687	Tumor
TCGA-OL-A66J-01A-11R-A29R-07	4.10274210418416	Tumor
TCGA-A2-A1G6-01A-11R-A13Q-07	4.37896239502459	Tumor
TCGA-E2-A14V-01A-11R-A12D-07	4.41490674911606	Tumor
TCGA-AO-A03R-01A-21R-A034-07	3.86934830973407	Tumor
TCGA-A8-A06T-01A-11R-A00Z-07	4.27287421768837	Tumor
TCGA-AN-A0FK-01A-11R-A034-07	4.15016158079386	Tumor
TCGA-A8-A0A2-01A-11R-A034-07	4.20784601013283	Tumor
TCGA-AN-A049-01A-21R-A00Z-07	4.76267835461692	Tumor
TCGA-EW-A1P8-01A-11R-A144-07	6.94684663598193	Tumor

TCGA-AN-A0AM-01A-11R-A034-07	4.79954843875837	Tumor
TCGA-A8-A09G-01A-21R-A00Z-07	5.25336154222225	Tumor
TCGA-A7-A26E-01A-11R-A169-07	4.85766754801889	Tumor
TCGA-A2-A0T5-01A-21R-A084-07	4.78563947508898	Tumor
TCGA-A2-A25D-01A-12R-A16F-07	4.20896978679481	Tumor
TCGA-D8-A1Y1-01A-21R-A14M-07	2.74977022822989	Tumor
TCGA-3C-AALJ-01A-31R-A41B-07	5.59848431327342	Tumor
TCGA-D8-A141-01A-11R-A115-07	4.45093408778932	Tumor
TCGA-A2-A4S3-01A-21R-A266-07	4.6073895000244	Tumor
TCGA-B6-A0I9-01A-11R-A034-07	6.24402125844716	Tumor
TCGA-AR-A2LQ-01A-22R-A18M-07	4.06102441810283	Tumor
TCGA-BH-A0BZ-01A-31R-A12P-07	5.47392325348787	Tumor
TCGA-B6-A401-01A-11R-A239-07	4.92808128126671	Tumor
TCGA-E9-A2JS-01A-11R-A180-07	5.47117445135592	Tumor
TCGA-AR-A24X-01A-11R-A169-07	4.42588222205097	Tumor
TCGA-BH-A1FE-06A-11R-A213-07	0.131062616782468	Tumor
TCGA-BH-A18K-01A-11R-A12D-07	5.06372726739535	Tumor
TCGA-AN-A04D-01A-21R-A034-07	4.00821794519928	Tumor
TCGA-BH-A1ES-01A-11R-A137-07	4.65893122229766	Tumor
TCGA-EW-A1P5-01A-11R-A144-07	5.55533149497076	Tumor
TCGA-AO-A0JG-01A-31R-A084-07	6.04799635603865	Tumor
TCGA-EW-A1P4-01A-21R-A144-07	4.53007074222508	Tumor

TCGA-C8-A26Y-01A-11R-A16F-07	8.05090051658692	Tumor
TCGA-A8-A07I-01A-11R-A00Z-07	4.67560707522375	Tumor
TCGA-E9-A1RC-01A-11R-A157-07	4.68669100638056	Tumor
TCGA-D8-A13Z-01A-11R-A115-07	3.74920171227094	Tumor
TCGA-BH-A42V-01A-11R-A24H-07	4.69530346978517	Tumor
TCGA-BH-A0HL-01A-11R-A10U-07	5.04079163129955	Tumor
TCGA-A2-A4RY-01A-31R-A266-07	4.93185322075111	Tumor
TCGA-EW-A1IY-01A-11R-A13Q-07	4.36764364215783	Tumor
TCGA-A8-A082-01A-11R-A00Z-07	5.31321319484006	Tumor
TCGA-B6-A0RU-01A-11R-A084-07	3.66842551730718	Tumor
TCGA-AR-A0TV-01A-21R-A084-07	4.52655714139591	Tumor
TCGA-A2-A0EN-01A-13R-A084-07	3.93157905825607	Tumor
TCGA-GM-A5PV-01A-11R-A28M-07	4.40317226676856	Tumor
TCGA-BH-A1F2-01A-31R-A13Q-07	3.46091289919721	Tumor
TCGA-E9-A1NG-01A-21R-A14M-07	3.7210882487981	Tumor
TCGA-AC-A2B8-01A-11R-A17B-07	4.99671864838065	Tumor
TCGA-GM-A2DB-01A-31R-A18M-07	4.42650630202183	Tumor
TCGA-AO-A12C-01A-11R-A10J-07	3.94814292471677	Tumor
TCGA-AC-A2BK-01A-11R-A21T-07	0.366587995394262	Tumor
TCGA-AC-A3QP-01A-11R-A22U-07	3.97486883815522	Tumor
TCGA-E2-A15A-06A-11R-A12D-07	4.83911306555634	Tumor
TCGA-A2-A0YI-01A-31R-A10J-07	3.90318291811272	Tumor

TCGA-A8-A07R-01A-21R-A034-07	3.56918725794523	Tumor
TCGA-BH-A0DT-01A-21R-A12D-07	4.84353670004563	Tumor
TCGA-A8-A08A-01A-11R-A32Y-07	5.64937978046493	Tumor
TCGA-AC-A23H-01A-11R-A157-07	5.85171906302582	Tumor
TCGA-B6-A0WV-01A-11R-A109-07	4.97987901195622	Tumor
TCGA-C8-A12V-01A-11R-A115-07	3.70388696678746	Tumor
TCGA-A2-A1G1-01A-21R-A13Q-07	4.77824012186314	Tumor
TCGA-E9-A6HE-01A-11R-A31O-07	4.4451373765995	Tumor
TCGA-JL-A3YX-01A-11R-A22U-07	5.28623060583327	Tumor
TCGA-PE-A5DC-01A-12R-A27Q-07	5.11515396203033	Tumor
TCGA-AC-A6IW-01A-12R-A33J-07	2.5566501253892	Tumor
TCGA-AC-A3TN-01A-11R-A22K-07	4.9436494480704	Tumor
TCGA-EW-A1P0-01A-11R-A144-07	4.97304639253857	Tumor
TCGA-BH-A0GY-01A-11R-A056-07	4.17649831113612	Tumor
TCGA-D8-A1X8-01A-11R-A14M-07	4.35450152845451	Tumor
TCGA-A7-A13D-01A-13R-A12P-07	5.01058293145219	Tumor
TCGA-BH-A18H-01A-11R-A12D-07	4.50961984194878	Tumor
TCGA-C8-A1HN-01A-11R-A137-07	4.55131913322916	Tumor
TCGA-E2-A106-01A-11R-A10J-07	5.63869944876237	Tumor
TCGA-A2-A0YE-01A-11R-A109-07	5.7533076879237	Tumor
TCGA-A7-A4SD-01A-11R-A266-07	4.2622226615488	Tumor
TCGA-E9-A227-01A-11R-A157-07	4.88083732836147	Tumor

TCGA-EW-A1J6-01A-11R-A13Q-07	3.17681741732755	Tumor
TCGA-AQ-A04J-01A-02R-A034-07	4.1092100966874	Tumor
TCGA-B6-A0RG-01A-11R-A056-07	4.34877771836267	Tumor
TCGA-A8-A09X-01A-11R-A00Z-07	4.95516863834393	Tumor
TCGA-A2-A0YH-01A-11R-A109-07	3.50481104434853	Tumor
TCGA-AN-A041-01A-11R-A034-07	4.18010011009102	Tumor
TCGA-E2-A1IE-01A-11R-A13Q-07	5.29307246714942	Tumor
TCGA-A8-A079-01A-21R-A00Z-07	3.89081631768988	Tumor
TCGA-AO-A12D-01A-11R-A115-07	5.87976702071562	Tumor
TCGA-BH-A28Q-01A-11R-A16F-07	4.75197245247521	Tumor
TCGA-B6-A0I2-01A-11R-A034-07	5.27921497535319	Tumor
TCGA-E2-A14Q-01A-11R-A12D-07	5.17477791101187	Tumor
TCGA-GI-A2C8-01A-11R-A16F-07	5.30565320988202	Tumor
TCGA-C8-A27A-01A-11R-A169-07	3.86971351025538	Tumor
TCGA-AC-A2FG-01A-11R-A17B-07	4.71808758396052	Tumor
TCGA-A8-A07W-01A-11R-A00Z-07	3.54500460365815	Tumor
TCGA-A8-A09B-01A-11R-A00Z-07	4.92820922492337	Tumor
TCGA-B6-A0IG-01A-11R-A034-07	4.89915145643346	Tumor
TCGA-PL-A8LV-01A-21R-A41B-07	4.89543835238485	Tumor
TCGA-A8-A06N-01A-11R-A00Z-07	4.30220936393274	Tumor
TCGA-AR-A5QP-01A-11R-A28M-07	4.72731058286584	Tumor
TCGA-BH-A0W3-01A-11R-A109-07	5.3002006174332	Tumor

TCGA-AN-A0XT-01A-11R-A109-07	4.7135473591639	Tumor
TCGA-BH-A1FD-01A-11R-A13Q-07	4.96731619026928	Tumor
TCGA-AR-A1AN-01A-11R-A12P-07	5.48814220459144	Tumor
TCGA-BH-A0HX-01A-21R-A056-07	3.67540948095381	Tumor
TCGA-AN-A0AL-01A-11R-A00Z-07	4.43771379411032	Tumor
TCGA-EW-A6SD-01A-12R-A33J-07	4.32524248339404	Tumor
TCGA-A2-A0D3-01A-11R-A115-07	4.71769287175219	Tumor
TCGA-C8-A12L-01A-11R-A115-07	6.3838926080387	Tumor
TCGA-AO-A12F-01A-11R-A115-07	3.23687524536694	Tumor
TCGA-BH-A0DZ-01A-11R-A00Z-07	2.81526800370087	Tumor
TCGA-AC-A3W5-01A-11R-A22K-07	5.27506293594629	Tumor
TCGA-E2-A15H-01A-11R-A12D-07	5.80371267579766	Tumor
TCGA-E2-A108-01A-13R-A10J-07	5.01863862469685	Tumor
TCGA-AQ-A1H2-01A-11R-A13Q-07	4.73961591378109	Tumor
TCGA-D8-A1JK-01A-11R-A13Q-07	4.77248217746889	Tumor
TCGA-GM-A2DN-01A-11R-A180-07	4.79269920808604	Tumor
TCGA-B6-A0RL-01A-11R-A084-07	4.87145927281086	Tumor
TCGA-BH-A0BW-01A-11R-A115-07	3.70303423484954	Tumor
TCGA-A8-A08O-01A-21R-A056-07	3.93121973196757	Tumor
TCGA-EW-A1J5-01A-11R-A13Q-07	4.71485544262265	Tumor
TCGA-A8-A06P-01A-11R-A00Z-07	4.37929518225635	Tumor
TCGA-GM-A2DO-01A-11R-A18M-07	3.20216693257981	Tumor

TCGA-HN-A2OB-01A-21R-A27Q-07	4.53343252796627	Tumor
TCGA-AN-A04C-01A-21R-A034-07	4.5519527589686	Tumor
TCGA-AR-A24W-01A-11R-A169-07	3.93555403851745	Tumor
TCGA-E2-A1IF-01A-11R-A144-07	5.15943947251191	Tumor
TCGA-GM-A3XG-01A-31R-A24H-07	4.82719406008167	Tumor
TCGA-A2-A0ER-01A-21R-A034-07	4.39534390566866	Tumor
TCGA-A2-A0D4-01A-11R-A00Z-07	4.05627137307508	Tumor
TCGA-AC-A6IX-06A-11R-A32P-07	6.2861418728343	Tumor
TCGA-GM-A2DM-01A-11R-A180-07	5.24333014794916	Tumor
TCGA-AQ-A04L-01B-21R-A10J-07	4.3848510814844	Tumor
TCGA-AC-A2FK-01A-12R-A180-07	5.35652767946236	Tumor
TCGA-A2-A0CY-01A-12R-A034-07	4.20249608419658	Tumor
TCGA-A8-A08I-01A-11R-A00Z-07	2.6336172025615	Tumor
TCGA-OL-A5RZ-01A-11R-A28M-07	5.72216269874248	Tumor
TCGA-A8-A08C-01A-11R-A00Z-07	4.00113567523125	Tumor
TCGA-A8-A09C-01A-11R-A00Z-07	4.44015490868031	Tumor
TCGA-AN-A0AS-01A-11R-A00Z-07	4.04375569486029	Tumor
TCGA-BH-A0DE-01A-11R-A115-07	4.36838423103146	Tumor
TCGA-D8-A1JG-01B-11R-A13Q-07	5.14686698430455	Tumor
TCGA-A8-A09Z-01A-11R-A00Z-07	2.48720333932614	Tumor
TCGA-BH-A28O-01A-11R-A22K-07	4.37797051114853	Tumor
TCGA-C8-A132-01A-31R-A115-07	4.35043359736946	Tumor

TCGA-EW-A1PB-01A-11R-A144-07	5.38566916576436	Tumor
TCGA-A8-A07P-01A-11R-A00Z-07	3.5528074051893	Tumor
TCGA-MS-A51U-01A-31R-A266-07	4.43592190333681	Tumor
TCGA-A2-A0CO-01A-13R-A22K-07	4.34323724728342	Tumor
TCGA-BH-A6R8-01A-21R-A33J-07	4.41950518329194	Tumor
TCGA-EW-A1OZ-01A-11R-A144-07	3.68094316504402	Tumor
TCGA-A2-A0EW-01A-21R-A115-07	4.57488260499984	Tumor
TCGA-B6-A3ZX-01A-11R-A239-07	3.95770105653688	Tumor
TCGA-S3-AA14-01A-11R-A41B-07	4.74933581641296	Tumor
TCGA-AO-A03N-01B-11R-A10J-07	3.66083736769628	Tumor
TCGA-AR-A1AP-01A-11R-A12P-07	5.29440386887753	Tumor
TCGA-A2-A0T0-01A-22R-A084-07	4.17699289565178	Tumor
TCGA-AN-A0XS-01A-22R-A109-07	3.40333590102322	Tumor
TCGA-D8-A73U-01A-11R-A33J-07	4.33081651322948	Tumor
TCGA-BH-A0DV-01A-21R-A12P-07	3.87693925674394	Tumor
TCGA-AR-A24Q-01A-12R-A169-07	2.82283210158867	Tumor
TCGA-BH-A0BS-01A-11R-A12P-07	5.51393914939894	Tumor
TCGA-AN-A0FV-01A-11R-A00Z-07	4.48245464977752	Tumor
TCGA-A2-A3KC-01A-11R-A213-07	4.16818470319679	Tumor
TCGA-B6-A0IJ-01A-11R-A034-07	4.044490296	Tumor
TCGA-AR-A24N-01A-11R-A169-07	3.99260530276066	Tumor
TCGA-D8-A1XU-01A-11R-A14M-07	6.30351592541613	Tumor

TCGA-A8-A08T-01A-21R-A00Z-07	4.3231178275988	Tumor
TCGA-BH-A18T-01A-11R-A12D-07	5.53018938003123	Tumor
TCGA-LL-A440-01A-11R-A24H-07	3.91583636616852	Tumor
TCGA-BH-A0AU-01A-11R-A12P-07	4.4007223532411	Tumor
TCGA-AR-A2LH-01A-31R-A18M-07	5.30426643461787	Tumor
TCGA-A7-A26E-01A-11R-A277-07	3.05048428400379	Tumor
TCGA-OL-A6VQ-01A-12R-A41B-07	4.40730515909546	Tumor
TCGA-D8-A1X9-01A-12R-A157-07	4.45169247946224	Tumor
TCGA-A8-A09D-01A-11R-A00Z-07	3.63658982144961	Tumor
TCGA-D8-A1X6-01A-11R-A14M-07	4.73245373108317	Tumor
TCGA-A8-A084-01A-21R-A00Z-07	5.49010477341522	Tumor
TCGA-A7-A0DA-01A-31R-A115-07	3.60980223187776	Tumor
TCGA-BH-A1EV-01A-11R-A137-07	5.26393956324211	Tumor
TCGA-D8-A1JM-01A-11R-A13Q-07	5.98503585142558	Tumor
TCGA-A8-A09W-01A-11R-A00Z-07	3.87414775459479	Tumor
TCGA-E2-A56Z-01A-12R-A29R-07	3.74389191093657	Tumor
TCGA-A1-A0SF-01A-11R-A144-07	4.91775634912606	Tumor
TCGA-E9-A22A-01A-11R-A157-07	4.77538772809043	Tumor
TCGA-A8-A0A6-01A-12R-A056-07	4.47644649720948	Tumor
TCGA-AN-A0XN-01A-21R-A109-07	3.08093048767298	Tumor
TCGA-A2-A0CR-01A-11R-A22K-07	4.26192189957288	Tumor
TCGA-B6-A0X5-01A-21R-A109-07	4.77265108102966	Tumor

TCGA-E9-A22G-01A-11R-A157-07	3.27570735959614	Tumor
TCGA-AR-A24K-01A-11R-A169-07	2.9175081369573	Tumor
TCGA-BH-A0BG-01A-11R-A115-07	3.25466962231078	Tumor
TCGA-E2-A14R-01A-11R-A115-07	3.56579212147183	Tumor
TCGA-E9-A247-01A-11R-A169-07	3.292693418	Tumor
TCGA-AR-A0TQ-01A-11R-A084-07	4.09105661262748	Tumor
TCGA-D8-A27L-01A-11R-A16F-07	4.40237428388649	Tumor
TCGA-A2-A0CL-01A-11R-A115-07	3.41601549528914	Tumor
TCGA-AR-A1AY-01A-21R-A12P-07	3.70211449145074	Tumor
TCGA-GM-A3XL-01A-11R-A22U-07	5.06263982828646	Tumor
TCGA-LL-A6FP-01A-11R-A31O-07	4.42682159461384	Tumor
TCGA-E9-A1RD-01A-11R-A157-07	4.67852267688349	Tumor
TCGA-AR-A1AO-01A-11R-A12P-07	3.09125976619268	Tumor
TCGA-WT-AB44-01A-11R-A41B-07	4.20422681254806	Tumor
TCGA-E2-A573-01A-11R-A29R-07	4.2625910095407	Tumor
TCGA-A8-A09M-01A-11R-A00Z-07	4.96998391084002	Tumor
TCGA-4H-AAAK-01A-12R-A41B-07	4.41814271206825	Tumor
TCGA-AC-A3TM-01A-11R-A22K-07	4.60576691725154	Tumor
TCGA-BH-A18N-01A-11R-A12D-07	4.91498062551314	Tumor
TCGA-BH-A18V-01A-11R-A12D-07	4.30401448236274	Tumor
TCGA-BH-A1FN-01A-11R-A13Q-07	4.45872321263061	Tumor
TCGA-GM-A3NW-01A-21R-A22K-07	5.12087029502508	Tumor

TCGA-A2-A04V-01A-21R-A034-07	3.39689928352096	Tumor
TCGA-C8-A12W-01A-11R-A115-07	2.74904077084432	Tumor
TCGA-BH-A0E6-01A-11R-A034-07	4.02635744696384	Tumor
TCGA-A8-A08J-01A-11R-A00Z-07	4.76451601379795	Tumor
TCGA-LL-A9Q3-01A-11R-A41B-07	5.29433769560231	Tumor
TCGA-A8-A06O-01A-11R-A00Z-07	4.56789220766005	Tumor
TCGA-A8-A08G-01A-11R-A00Z-07	5.21476594703247	Tumor
TCGA-A7-A13G-01B-04R-A22O-07	4.49442201098242	Tumor
TCGA-D8-A1XA-01A-11R-A14D-07	1.85335696860212	Tumor
TCGA-A7-A13G-01A-11R-A13Q-07	4.20040234046558	Tumor
TCGA-BH-A1EN-01A-11R-A13Q-07	7.13891990725321	Tumor
TCGA-C8-A12Y-01A-11R-A12D-07	4.36989915747906	Tumor
TCGA-AO-A12E-01A-11R-A10J-07	4.01728947712788	Tumor
TCGA-A8-A07J-01A-11R-A00Z-07	5.24942258546409	Tumor
TCGA-A7-A13F-01A-11R-A12P-07	5.14783580703476	Tumor
TCGA-A2-A0YD-01A-11R-A109-07	4.4778068275915	Tumor
TCGA-5T-A9QA-01A-11R-A41B-07	4.72684756167224	Tumor
TCGA-BH-A42U-01A-12R-A24H-07	3.96800778317157	Tumor
TCGA-E2-A1LI-01A-12R-A157-07	2.33973600766177	Tumor
TCGA-A8-A09N-01A-11R-A00Z-07	5.91476063250101	Tumor
TCGA-C8-A135-01A-11R-A115-07	3.35366204474506	Tumor
TCGA-D8-A1XY-01A-11R-A14M-07	3.7711883538963	Tumor

TCGA-AN-A0XW-01A-11R-A109-07	4.92341068685346	Tumor
TCGA-BH-A201-01A-11R-A14M-07	5.10149460488992	Tumor
TCGA-A8-A085-01A-11R-A00Z-07	5.44019478361407	Tumor
TCGA-LD-A74U-01A-13R-A33J-07	4.78511631783786	Tumor
TCGA-A8-A0A1-01A-11R-A00Z-07	3.63082479862984	Tumor
TCGA-GM-A2DK-01A-21R-A180-07	3.65031651895902	Tumor
TCGA-LL-A442-01A-11R-A24H-07	4.65766858446201	Tumor
TCGA-A2-A259-01A-11R-A16F-07	4.66618848987278	Tumor
TCGA-AC-A2FF-01A-11R-A17B-07	4.48278376038052	Tumor
TCGA-C8-A1HI-01A-11R-A137-07	5.08498692023195	Tumor
TCGA-LL-A73Z-01A-11R-A32P-07	4.34135247286269	Tumor
TCGA-AO-A0JM-01A-21R-A056-07	3.95679082709533	Tumor
TCGA-OL-A5RX-01A-11R-A28M-07	4.96375153865527	Tumor
TCGA-BH-A0DH-01A-11R-A084-07	4.45241752472873	Tumor
TCGA-AO-A0JB-01A-11R-A32Y-07	3.01539051939286	Tumor
TCGA-A2-A0T7-01A-21R-A084-07	5.44611062946511	Tumor
TCGA-E9-A1R3-01A-31R-A14M-07	5.42718040766199	Tumor
TCGA-AN-A0XL-01A-11R-A10J-07	3.7586975934794	Tumor
TCGA-E2-A10A-01A-21R-A115-07	5.61453023212383	Tumor
TCGA-EW-A1PH-01A-11R-A14M-07	3.03599333310973	Tumor
TCGA-S3-AA17-01A-11R-A41B-07	5.59005403376222	Tumor
TCGA-B6-A0X4-01A-11R-A109-07	5.52437089296449	Tumor

TCGA-A2-A3XX-01A-21R-A239-07	3.82796125514531	Tumor
TCGA-OL-A5DA-01A-11R-A27Q-07	5.0133729260785	Tumor
TCGA-EW-A1PA-01A-11R-A144-07	4.05470090136319	Tumor
TCGA-EW-A1OY-01A-11R-A144-07	4.09635463331544	Tumor
TCGA-D8-A1JH-01A-11R-A13Q-07	5.14664707679927	Tumor
TCGA-AR-A1AT-01A-11R-A12P-07	5.08839180743585	Tumor
TCGA-AC-A7VB-01A-11R-A352-07	4.82675693213201	Tumor
TCGA-AN-A0FJ-01A-11R-A00Z-07	2.6399267127488	Tumor
TCGA-A2-A1FW-01A-11R-A13Q-07	4.19040152151154	Tumor
TCGA-BH-A8G0-01A-11R-A352-07	4.55846357253775	Tumor
TCGA-A7-A6VV-01A-22R-A33J-07	4.31333293336707	Tumor
TCGA-EW-A6SB-01A-12R-A32P-07	2.93073733756289	Tumor
TCGA-E2-A15I-01A-21R-A137-07	5.29604619904214	Tumor
TCGA-EW-A1IW-01A-11R-A13Q-07	4.30579546142049	Tumor
TCGA-D8-A1J8-01A-11R-A13Q-07	5.08890057643629	Tumor
TCGA-D8-A1XO-01A-11R-A14M-07	4.80158808890509	Tumor
TCGA-E9-A22E-01A-11R-A157-07	3.22724818378073	Tumor
TCGA-BH-A0B0-01A-21R-A115-07	5.40018267684189	Tumor
TCGA-GM-A2DI-01A-31R-A18M-07	3.90523535610952	Tumor
TCGA-A7-A26J-01A-11R-A277-07	2.73333264578844	Tumor
TCGA-B6-A0I8-01A-11R-A034-07	5.34328700041261	Tumor
TCGA-3C-AAAU-01A-11R-A41B-07	5.16276995504434	Tumor

TCGA-A7-A26J-01B-02R-A277-07	3.37189336181144	Tumor
TCGA-AR-A2LJ-01A-12R-A19W-07	4.10344728297666	Tumor
TCGA-BH-A0H3-01A-11R-A12P-07	4.30064358697458	Tumor
TCGA-C8-A26X-01A-31R-A16F-07	3.72663506691069	Tumor
TCGA-BH-A18G-01A-11R-A12D-07	4.5087645661338	Tumor
TCGA-E2-A14N-01A-31R-A137-07	4.4278810095045	Tumor
TCGA-A8-A075-01A-11R-A084-07	5.52246069680715	Tumor
TCGA-E2-A1L7-01A-11R-A144-07	4.57568142943099	Tumor
TCGA-C8-A12T-01A-11R-A115-07	3.99067355280783	Tumor
TCGA-A2-A0YJ-01A-11R-A109-07	5.02110656836861	Tumor
TCGA-A2-A25C-01A-11R-A169-07	4.80069803788265	Tumor
TCGA-BH-A0C0-01A-21R-A056-07	4.40067454196401	Tumor
TCGA-A7-A6VX-01A-12R-A33J-07	5.1905437508154	Tumor
TCGA-AN-A0FZ-01A-11R-A034-07	3.89055369913251	Tumor
TCGA-A8-A07E-01A-11R-A034-07	4.72537034822428	Tumor
TCGA-AQ-A04H-01B-11R-A10J-07	3.80399158619985	Tumor
TCGA-D8-A1XW-01A-11R-A14M-07	3.80020620443402	Tumor
TCGA-AN-A03X-01A-21R-A00Z-07	3.60783923637989	Tumor
TCGA-A7-A13E-01A-11R-A277-07	2.14844607552888	Tumor
TCGA-A7-A13E-01B-06R-A277-07	1.65978758718141	Tumor
TCGA-A8-A08L-01A-11R-A00Z-07	4.52746449482964	Tumor
TCGA-AC-A6IV-01A-12R-A33J-07	2.83321395141742	Tumor

TCGA-E2-A10F-01A-11R-A10J-07	5.06987828289486	Tumor
TCGA-W8-A86G-01A-21R-A36F-07	5.39262997284592	Tumor
TCGA-C8-A130-01A-31R-A115-07	5.0628081760539	Tumor
TCGA-A8-A07G-01A-11R-A034-07	4.81670209431686	Tumor
TCGA-A7-A3J1-01A-11R-A213-07	4.84031719451948	Tumor
TCGA-D8-A1XM-01A-21R-A14M-07	5.40516529506997	Tumor
TCGA-BH-A0BL-01A-11R-A115-07	3.73400503372005	Tumor
TCGA-BH-A0AY-01A-21R-A00Z-07	4.94558897648869	Tumor
TCGA-AC-A3QQ-01B-06R-A22O-07	3.38740390191284	Tumor
TCGA-AO-A0J7-01A-11R-A034-07	4.77823486433353	Tumor
TCGA-E9-A1N6-01A-11R-A144-07	5.32271414950421	Tumor
TCGA-AN-A0G0-01A-11R-A034-07	5.179136793	Tumor
TCGA-E9-A3X8-01A-31R-A22U-07	3.0247274630775	Tumor
TCGA-BH-A0H0-01A-11R-A056-07	4.56070272819351	Tumor
TCGA-E2-A10C-01A-21R-A10J-07	5.08628274337583	Tumor
TCGA-A8-A094-01A-11R-A00Z-07	4.93931121837025	Tumor
TCGA-AC-A5XU-01A-11R-A28M-07	3.94398694471869	Tumor
TCGA-AR-A0U2-01A-11R-A109-07	4.90103098819959	Tumor
TCGA-BH-A1EW-01A-11R-A137-07	4.64409277236219	Tumor
TCGA-EW-A6SC-01A-12R-A32P-07	4.30810599559071	Tumor

Supplementary Table S2: TMEscores

ID	StromalScore	ImmuneScore	ESTIMATEScore	TumorPurity
TCGA-B6-A0RH-01A-21R-A115-07	779.433468966664	360.184814037775	1139.61828300444	0.716328631874749
TCGA-BH-A1FU-01A-11R-A14D-07	1324.84474443257	1059.00777871596	2383.85252314853	0.577682958796007
TCGA-AR-A0TX-01A-11R-A084-07	1073.44958771658	1804.93915050753	2878.38873822411	0.516959386341834
TCGA-A1-A0SE-01A-11R-A084-07	769.608410087251	23.9287633691888	793.53717345644	0.750836114569339
TCGA-OL-A5D6-01A-21R-A27Q-07	1129.72365099156	675.249145041066	1804.97279603263	0.644875256175583
TCGA-E2-A1IK-01A-11R-A144-07	19.945513096327	1205.89888905264	1225.84440214896	0.707439907757039
TCGA-AN-A0FT-01A-11R-A034-07	896.69836374703	693.410506595094	1590.10887034212	0.668655824985279
TCGA-A2-A0EO-01A-11R-A034-07	1381.89190291646	1012.98051352703	2394.87241644348	0.576361825536581
TCGA-A2-A04P-01A-31R-A034-07	519.985517215395	1312.03061909583	1832.01613631122	0.641836228946199
TCGA-AC-A5EH-01A-11R-A28M-07	1137.51175436331	927.888805491999	2065.40055985531	0.6151942073591
TCGA-A7-A0DC-01B-04R-A22O-07	200.509106276712	-421.1268748	-220.6177685	0.840493423024229
TCGA-OK-A5Q2-01A-11R-A27Q-07	849.88448301323	1439.06934777235	2288.95383078558	0.588997084714565
TCGA-BH-A0DO-01B-11R-A12D-07	328.516351845674	-40.29477775	288.221574093619	0.797718042645511
TCGA-BH-A18J-01A-11R-A12D-07	787.439521610442	242.841582120487	1030.28110373093	0.727434594778467
TCGA-A7-A0CJ-01A-21R-A00Z-07	-261.6778548	119.154451172402	-142.5234036	0.834227245111842
TCGA-A8-A09V-01A-11R-A034-07	713.689680697955	-303.3403885	410.349292234022	0.786779952764507
TCGA-C8-A275-01A-21R-A16F-07	610.661765214358	1834.12478460898	2444.78654982334	0.570358974551522
TCGA-E2-A1LA-01A-11R-A144-07	788.581432840138	1800.45302102013	2589.03445386026	0.552840260231012
TCGA-AC-A8OS-01A-12R-A41B-07	1246.50096180645	1127.41891000561	2373.91987181206	0.578872451609204
TCGA-B6-A0WT-01A-11R-A109-07	780.662595343817	5.44580886581635	786.108404209634	0.751555902484817
TCGA-EW-A1PG-01A-	963.6087357	214.4377002	1178.046435	0.712381309

11R-A144-07	08405	06881	91529	630496
TCGA-A8-A08H-01A-21R-A00Z-07	2026.164971 26639	2543.704853 60798	4569.869824 87436	0.290744868 731838
TCGA-AC-A5XS-01A-11R-A29R-07	950.7272829 67953	1653.929431 56698	2604.656714 53494	0.550927941 781466
TCGA-A8-A097-01A-11R-A034-07	1051.220050 9483	734.8718256 07793	1786.091876 55609	0.646991000 474892
TCGA-E2-A15C-01A-31R-A12D-07	780.0783680 45816	320.9492314 84491	1101.027599 53031	0.720269711 613284
TCGA-C8-A12M-01A-11R-A115-07	- 179.0356874	51.37434826 13767	- 127.6613391	0.833022293 038106
TCGA-E2-A14Z-01A-11R-A115-07	890.9023278 33248	1849.682755 62832	2740.585083 46157	0.534167796 671724
TCGA-A7-A4SF-01A-11R-A266-07	8.938911637 45457	402.4867102 21011	411.4256218 58466	0.786682424 541524
TCGA-BH-A0W5-01A-11R-A109-07	1099.194563 52035	2087.418709 1261	3186.613272 64645	0.477714450 243352
TCGA-BH-A0C7-01B-11R-A115-07	327.6450667 5823	425.8554107 75244	753.5004775 33473	0.754704766 055273
TCGA-BH-A0HU-01A-11R-A034-07	- 153.3716106	61.84608953 42219	- 91.52552102	0.830076029 613556
TCGA-E9-A226-01A-21R-A157-07	901.0994102 33528	- 17.02687926	884.0725309 72343	0.741992481 395594
TCGA-E2-A14T-01A-11R-A115-07	84.40173721 24606	373.6536349 4153	458.0553721 5399	0.782438396 053818
TCGA-AN-A046-01A-21R-A034-07	1024.913379 66664	1548.350847 75141	2573.264227 41805	0.554767742 509468
TCGA-BH-A1F5-01A-12R-A13Q-07	227.7343926 68423	- 211.1487347	16.58565793 40382	0.821122268 086069
TCGA-AO-A0J6-01A-11R-A034-07	- 536.8312634	1356.689285 91868	819.8580225 17172	0.748278656 437599
TCGA-AC-A23E-01A-11R-A157-07	1710.838963 35372	796.7376747 91306	2507.576638 14502	0.562764170 842975
TCGA-E2-A1IH-01A-11R-A13Q-07	1742.167409 90541	1994.938513 2898	3737.105923 1952	0.405243646 695483
TCGA-E2-A15T-01A-11R-A115-07	- 481.3094036	- 487.1488287	- 968.4582323	0.894793088 195029
TCGA-LL-A5YN-01A-11R-A28M-07	435.8094044 02306	2391.231880 05362	2827.041284 45593	0.523396568 280461
TCGA-EW-A2FW-01A-11R-A17B-07	263.2943780 44173	- 91.07777355	172.2166044 98319	0.807870412 646716
TCGA-A7-A0DB-01C-02R-A277-07	1195.775329 45151	142.6474604 68899	1338.422789 92041	0.695664272 473577

TCGA-A7-A0DB-01A-11R-A277-07	1304.08676403557	766.910693492025	2070.99745752759	0.614546302230484
TCGA-E9-A1R0-01A-22R-A16F-07	871.733574446053	660.662395494785	1532.39596994084	0.674930989101544
TCGA-BH-A1F8-01A-11R-A13Q-07	439.478738582937	157.199954770508	596.678693353446	0.769605837251633
TCGA-BH-A0BP-01A-11R-A115-07	1291.68801548508	441.498217883315	1733.18623336839	0.652892937985375
TCGA-BH-A18R-01A-11R-A12D-07	-61.93084194	429.237963775237	367.307121832939	0.790663961618801
TCGA-D8-A147-01A-11R-A115-07	89.0550838757225	955.485867059776	1044.5409509355	0.725996710102897
TCGA-E9-A1N5-01A-11R-A14D-07	1260.18597801301	1124.91938785345	2385.10536586645	0.577532836319988
TCGA-BH-A0HP-01A-12R-A084-07	916.393899895505	743.951443078365	1660.34534297387	0.660954271029471
TCGA-A8-A06R-01A-11R-A00Z-07	1190.20697228198	1096.87717558128	2287.08414786327	0.589218853417456
TCGA-A8-A086-01A-11R-A00Z-07	1027.40132786434	174.885199686378	1202.28652755072	0.709879711947383
TCGA-AR-A1AX-01A-11R-A12P-07	1060.60405567646	2242.93288529902	3303.53694097548	0.462566842649349
TCGA-BH-A0B6-01A-11R-A19W-07	1178.05201265257	2602.58608874571	3780.63810139829	0.399393602049369
TCGA-D8-A1JJ-01A-31R-A14M-07	1020.83238491382	565.959478774967	1586.79186368879	0.669017789527966
TCGA-OL-A66P-01A-11R-A31O-07	1191.45907524588	2660.98786292491	3852.44693817078	0.389708091451718
TCGA-E9-A295-01A-11R-A16F-07	677.089272089161	323.692521754354	1000.78179384351	0.730399019618397
TCGA-C8-A3M8-01A-11R-A213-07	323.085591209375	-145.9732248	177.112366373434	0.807446665668349
TCGA-AO-A1KO-01A-31R-A13Q-07	436.153528468539	97.8147151810809	533.96824364962	0.775450824385103
TCGA-AN-A0FN-01A-11R-A034-07	2176.71446469848	2164.61850850769	4341.33297320617	0.322672634324183
TCGA-BH-A0BM-01A-11R-A056-07	852.826796132009	297.898593991063	1150.72539012307	0.715190051264563
TCGA-BH-A18F-01A-11R-A12D-07	1111.39334115058	895.324015202499	2006.71735635308	0.621962377536008
TCGA-E2-A14S-01A-11R-A12D-07	216.036785682592	919.275154943788	1135.31194062638	0.716769559490877
TCGA-B6-A0RI-01A-11R-A056-07	908.058920071044	401.147174651301	1309.20609472235	0.698738706080032

TCGA-A2-A3XW-01A-11R-A239-07	672.776766195428	1361.67128587736	2034.44805207279	0.618769805459139
TCGA-3C-AALK-01A-11R-A41B-07	1030.00494164677	642.396424529995	1672.40136617676	0.659625219269045
TCGA-A2-A0EU-01A-22R-A056-07	335.780353494282	15.3291770635239	351.109530557806	0.792117423189152
TCGA-A2-A0YG-01A-21R-A109-07	-21.04769557	125.873527025929	104.825831453935	0.813660849667586
TCGA-AO-A0JJ-01A-11R-A056-07	1471.43981465905	1700.13345524686	3171.57326990592	0.479652780624582
TCGA-C8-A131-01A-11R-A115-07	711.459344484719	832.000421302763	1543.45976578748	0.673731753831393
TCGA-AR-A0TZ-01A-12R-A084-07	234.834499183274	-79.09968708	155.734812101502	0.809293907158891
TCGA-BH-A0EE-01A-11R-A034-07	-939.29613	800.994642505456	-138.3014875	0.833885353848208
TCGA-E2-A15D-01A-11R-A115-07	675.77681785932	430.71851359362	1106.49533145294	0.719712721042873
TCGA-AO-A1KQ-01A-11R-A13Q-07	444.745493172968	1155.14621725603	1599.89171	0.667587360935799
TCGA-A7-A426-01A-22R-A24H-07	792.19487830307	648.428770075658	1440.62364837873	0.684809525092715
TCGA-BH-A1FG-01A-11R-A13Q-07	76.1087032314289	172.379750309929	248.488453541358	0.801221547347173
TCGA-AQ-A0Y5-01A-11R-A14M-07	1184.50886022532	387.047810925829	1571.55667115115	0.670678273898681
TCGA-BH-A0DD-01A-31R-A12P-07	93.7638666067253	135.578397535915	229.342264142641	0.802900056811165
TCGA-AR-A24L-01A-11R-A169-07	1110.68060574467	158.145044083801	1268.82564982847	0.702966718431443
TCGA-E2-A1B4-01A-11R-A12P-07	95.789480713793	-225.4704963	-129.6810156	0.833186272594535
TCGA-E9-A1RI-01A-11R-A169-07	1393.41462109728	504.444843192479	1897.85946428976	0.6343948130489
TCGA-AN-A0FW-01A-11R-A034-07	529.827656799162	299.615354241872	829.443011041034	0.747344557197962
TCGA-AN-A04A-01A-21R-A034-07	1095.88602929479	309.530123245354	1405.41615254014	0.688566436889144
TCGA-AO-A03T-01A-21R-A034-07	-84.66099936	750.778887232334	666.117887869102	0.763057628585226
TCGA-AO-A0JI-01A-21R-A056-07	-352.8533448	-76.09498021	-428.948325	0.856667055048855
TCGA-BH-A0HA-01A-11R-A12P-07	1467.18493373568	1216.60686514908	2683.79179888476	0.541196731408893

TCGA-BH-A0BF-01A-21R-A12P-07	1867.73698993971	2370.82383373368	4238.56082367339	0.336914208104575
TCGA-A2-A1G4-01A-11R-A13Q-07	-193.4391979	245.275688235673	51.8364903294839	0.818157980265455
TCGA-A2-A25B-01A-11R-A169-07	786.749095301346	696.986979928653	1483.736075	0.680184195020955
TCGA-E2-A15O-01A-11R-A115-07	-347.2635141	23.3238694705421	-323.9396446	0.848613958154347
TCGA-BH-A0EB-01A-11R-A034-07	1409.25251252964	1542.06555166928	2951.31806419892	0.507766200114925
TCGA-E9-A1NC-01A-21R-A26B-07	929.726225486249	1765.26613445224	2694.99235993849	0.539813472990033
TCGA-AN-A0AR-01A-11R-A00Z-07	-617.0637539	-314.1214234	-931.1851772	0.89233689737891
TCGA-BH-A202-01A-11R-A14M-07	666.438980513729	1106.89183035267	1773.33081086639	0.648418157150874
TCGA-OL-A66N-01A-12R-A31O-07	811.681324245021	1133.23622607888	1944.9175503239	0.629040096826189
TCGA-EW-A1J1-01A-11R-A13Q-07	219.26177720155	983.450711994401	1202.71248919595	0.709835671903185
TCGA-EW-A1P1-01A-31R-A14D-07	1751.23534576608	1549.96926798548	3301.20461375156	0.462870345315801
TCGA-LL-A5YM-01A-11R-A28M-07	271.324100935556	605.672461461059	876.996562396616	0.742688413984687
TCGA-C8-A134-01A-11R-A115-07	99.081657248862	1206.72445751097	1305.80611475983	0.699095647683493
TCGA-AC-A3OD-01B-06R-A22O-07	1215.49517352583	2165.10811733389	3380.60329085972	0.452508020770859
TCGA-A8-A08P-01A-11R-A00Z-07	1170.68627601218	1833.17147687925	3003.85775289143	0.501107129940587
TCGA-A2-A04R-01A-41R-A109-07	172.832884231404	-513.9838699	-341.1509856	0.849947759270075
TCGA-BH-A1EU-01A-11R-A137-07	1608.84637109863	1871.44478070378	3480.29115180241	0.439410892391671
TCGA-AR-A1AW-01A-21R-A12P-07	1296.90224712724	3071.09182898925	4367.99407611649	0.318965964976173
TCGA-E2-A15P-01A-11R-A115-07	485.93785519746	-18.34144641	467.596408785621	0.781565490021363
TCGA-A2-A0YC-01A-11R-A109-07	817.207457460933	654.777324245699	1471.98478170663	0.681447644570595
TCGA-AN-A0XU-01A-11R-A109-07	-452.6804945	748.826976174013	296.146481673832	0.797016006883938
TCGA-A8-A07S-01A-11R-A034-07	356.791633382033	275.770174220486	632.561807602519	0.766231952383123

TCGA-JL-A3YW-01A-12R-A239-07	1655.99379099801	1750.74115430861	3406.73494530663	0.449084064705006
TCGA-C8-A1HF-01A-11R-A137-07	1074.8922324174	1588.33088158093	2663.22311399833	0.543733131342496
TCGA-A8-A07Z-01A-11R-A00Z-07	-129.2239192	681.220793341246	551.996874174389	0.773777156797249
TCGA-A2-A0ES-01A-11R-A115-07	1166.21576496629	770.061836359311	1936.2776013256	0.63002548935914
TCGA-A1-A0SJ-01A-11R-A084-07	696.996145455782	252.176422562897	949.172568018679	0.735552327295844
TCGA-A7-A4SA-01A-11R-A266-07	928.612763612713	1670.38929876279	2599.00206237551	0.551620461919883
TCGA-BH-A0BT-01A-11R-A12P-07	541.517062561095	587.263722764178	1128.78078532527	0.717437739696771
TCGA-EW-A1J2-01A-21R-A13Q-07	1356.34430355531	1075.35170799139	2431.69601154669	0.57193626432006
TCGA-E9-A5UP-01A-11R-A28M-07	-1080.731164	262.610237049493	-818.1209268	0.884723178315276
TCGA-OL-A66O-01A-11R-A31O-07	-64.50358099	-238.594909	-303.0984899	0.846991613184724
TCGA-AC-A3YI-01A-21R-A239-07	1283.3942251185	866.36000562636	2149.75423074486	0.605385520054217
TCGA-B6-A0WX-01A-11R-A109-07	1118.42424469243	950.869314963918	2069.29355965635	0.614743591898373
TCGA-AO-A0J9-01A-11R-A034-07	1504.81969341908	1808.03322361755	3312.85291703664	0.46135402654397
TCGA-E9-A244-01A-11R-A169-07	159.756055477568	69.0343395729055	228.790395050473	0.802948344140965
TCGA-EW-A424-01A-11R-A24H-07	560.232881318639	-240.5481816	319.684699683643	0.794924495185928
TCGA-BH-A0HN-01A-11R-A10U-07	-564.8085894	80.0513360059688	-484.7572534	0.860864320183175
TCGA-AO-A03V-01A-11R-A115-07	139.833274727377	45.4273575931468	185.260632320524	0.806740477508817
TCGA-AR-A1AH-01A-11R-A12D-07	-290.2813502	-311.0792383	-601.3605884	0.869447020657412
TCGA-BH-A18I-01A-11R-A12D-07	1511.16853059961	1291.80695082969	2802.9754814293	0.526403370194948
TCGA-D8-A3Z6-01A-11R-A239-07	655.536858291555	119.895393165997	775.432251457552	0.752588770449722
TCGA-D8-A27I-01A-11R-A16F-07	1468.70574773082	1359.78375514797	2828.48950287879	0.523215417911005
TCGA-A2-A3XS-01A-11R-A22U-07	289.777178983825	911.570749736167	1201.34792871999	0.70997674359798

TCGA-A2-A04Y-01A-21R-A034-07	546.312760287925	1356.45550706571	1902.76826735364	0.63383765249634
TCGA-D8-A1JA-01A-11R-A13Q-07	849.794358189975	346.37150786734	1196.16586605732	0.710512218736169
TCGA-E2-A152-01A-11R-A12D-07	1130.70596409459	549.941237801738	1680.64720189633	0.658715011329615
TCGA-AO-A0J4-01A-11R-A034-07	380.207703165754	1452.50184851031	1832.70955167606	0.641758172380422
TCGA-D8-A1XK-01A-21R-A14M-07	-284.7702024	982.675000296897	697.904797896633	0.760033587882749
TCGA-A2-A0CQ-01A-21R-A034-07	73.9242685158676	789.414899928143	863.339168444011	0.744029373732148
TCGA-AR-A1AI-01A-11R-A12P-07	-114.1728283	1815.35195212399	1701.17912380048	0.656444426393511
TCGA-B6-A2IU-01A-32R-A18M-07	-91.05268922	-261.6807953	-352.7334846	0.850842297246203
TCGA-D8-A1JL-01A-11R-A13Q-07	1147.24467648127	1766.24413857135	2913.48881505262	0.512542137915444
TCGA-AN-A0AK-01A-21R-A00Z-07	-261.0052261	298.932840406736	37.9276143	0.819330216425567
TCGA-E9-A24A-01A-11R-A169-07	1269.86096408666	1203.83009535631	2473.69105944296	0.566868795895339
TCGA-GI-A2C9-01A-11R-A21T-07	470.231400675231	605.65917368742	1075.89057436265	0.722824411682855
TCGA-BH-A0W7-01A-11R-A115-07	1313.37559365632	1775.76217144409	3089.13776510041	0.490235157310706
TCGA-BH-A0B5-01A-11R-A12P-07	781.11308704305	821.355852766306	1602.46893980936	0.667305651422055
TCGA-A2-A0CZ-01A-11R-A034-07	1570.9224993913	1213.50719925573	2784.42969864703	0.528716016317602
TCGA-A2-A0SV-01A-11R-A084-07	972.129446736657	1244.17853345195	2216.3079801886	0.597581038143131
TCGA-OL-A66L-01A-12R-A31O-07	994.490360855745	1428.88832126562	2423.37868212137	0.572937329448783
TCGA-AR-A2LO-01A-31R-A18M-07	971.902683668548	1476.10025827484	2448.00294194338	0.569971106297241
TCGA-D8-A1JU-01A-11R-A13Q-07	1875.29656615308	1120.10552304771	2995.40208920079	0.502180854093803
TCGA-AC-A8OP-01A-11R-A36F-07	619.221044285592	457.358476093357	1076.57952037895	0.722754524441772
TCGA-GM-A2DD-01A-11R-A180-07	1369.84534315544	2306.14892843671	3675.99427159214	0.413428124413516
TCGA-A7-A5ZX-01A-12R-A29R-07	873.780631489626	59.7554838699666	933.536115359592	0.737105346562003

TCGA-AO-A12H-01A-11R-A115-07	62.4189748156135	215.633482593163	278.052457408777	0.798617302429549
TCGA-C8-A1HE-01A-11R-A13Q-07	289.905387975021	307.112427637073	597.017815612093	0.769574051311003
TCGA-BH-A0BD-01A-11R-A034-07	900.935640515873	1153.22811310479	2054.16375362066	0.616493742492658
TCGA-E9-A22B-01A-11R-A157-07	1084.97049156989	571.302939298157	1656.27343086805	0.661402689766654
TCGA-E9-A228-01A-31R-A157-07	163.726779	306.198133108808	469.924912139108	0.781352223298194
TCGA-E2-A153-01A-12R-A12D-07	1620.57004926196	775.682634572663	2396.25268383463	0.576196244160017
TCGA-A1-A0SD-01A-11R-A115-07	1033.22688961477	513.997156594112	1547.22404620888	0.673323327819708
TCGA-AC-A2FM-01A-11R-A19W-07	58.1405998319873	-412.0526459	-353.9120461	0.850933181929995
TCGA-BH-A0B4-01A-11R-A00Z-07	1261.4071226572	861.674201073738	2123.08132373094	0.60849716488711
TCGA-A2-A0CX-01A-21R-A00Z-07	1150.66049461702	1031.92277267915	2182.58326729617	0.601542969866394
TCGA-BH-A0AW-01A-11R-A056-07	1266.37345113313	2247.38155834458	3513.75500947771	0.434993131855752
TCGA-A2-A0SU-01A-11R-A084-07	595.838039971044	581.45421094275	1177.29225091379	0.712458997643891
TCGA-D8-A1JT-01A-31R-A13Q-07	-202.5594312	233.569608698446	31.0101775152579	0.819911944699615
TCGA-E9-A1NI-01A-11R-A14D-07	853.909766186731	264.370154815487	1118.27992100222	0.718510664637666
TCGA-OL-A5D7-01A-11R-A27Q-07	-264.4579381	1805.48497927644	1541.02704117859	0.673995596059551
TCGA-E2-A570-01A-11R-A29R-07	940.397031746747	140.890030164351	1081.2870619111	0.722276789848056
TCGA-E2-A1LH-01A-11R-A14D-07	738.470807147905	2234.17955171247	2972.65035886037	0.505066087340376
TCGA-LL-A7T0-01A-31R-A352-07	153.446152934265	741.709569287416	895.155722221681	0.740900823624649
TCGA-A7-A4SE-01A-11R-A266-07	827.139027891444	423.439107158046	1250.57813504949	0.704869221276469
TCGA-A2-A3XY-01A-11R-A239-07	1226.2796481662	1612.10225780373	2838.38190596993	0.521977394942308
TCGA-C8-A26Z-01A-11R-A16F-07	274.93348361985	-338.934753	-64.00126933	0.82781621958987
TCGA-AR-A1AV-01A-21R-A12P-07	425.245941730531	105.857094713441	531.103036443972	0.775716312839021

TCGA-E2-A10E-01A-21R-A10J-07	245.868002298042	357.899368333109	603.767370631152	0.768941018713306
TCGA-E2-A14P-01A-31R-A12D-07	-227.4102467	723.464832177051	496.054585476947	0.778952762909701
TCGA-A7-A4SC-01A-12R-A266-07	898.565707225316	705.242877577059	1603.80858480238	0.667159180964221
TCGA-BH-A0RX-01A-21R-A084-07	1363.52287462783	2232.01492918131	3595.53780380914	0.424152551189171
TCGA-E2-A15F-01A-11R-A115-07	42.5007433065078	209.16785371389	251.668597020398	0.800942137077625
TCGA-E2-A107-01A-11R-A10J-07	-319.7419865	35.814206123277	-283.9277804	0.845492301501088
TCGA-E2-A109-01A-11R-A10J-07	409.644911030894	259.109040933136	668.75395196403	0.762807478014454
TCGA-E9-A3QA-01A-61R-A22K-07	480.843537858367	2253.63890265716	2734.48244051553	0.53492486991338
TCGA-E2-A576-01A-11R-A31O-07	81.6220885609437	301.604376177279	383.226464738223	0.789231112953713
TCGA-A8-A099-01A-11R-A00Z-07	427.272606067204	334.414529140392	761.687135207597	0.753915824334436
TCGA-BH-A0HQ-01A-11R-A034-07	745.478884693632	106.526022088989	852.004906782621	0.745139964964455
TCGA-AN-A0XR-01A-11R-A109-07	-39.95893658	-242.4709658	-282.4299024	0.845374872612274
TCGA-S3-A6ZF-01A-32R-A32P-07	449.05613413764	865.772322621902	1314.82845675954	0.698148069334735
TCGA-E9-A1RF-01A-11R-A157-07	1164.09509051197	1382.17880238773	2546.2738928997	0.558059674993825
TCGA-D8-A27P-01A-11R-A16F-07	326.883211886454	-155.4402796	171.442932243475	0.80793733877892
TCGA-D8-A142-01A-11R-A115-07	1107.11958109104	866.007737152584	1973.12731824362	0.625815715658951
TCGA-AR-A0U3-01A-11R-A109-07	-325.7519826	173.363718089806	-152.3882645	0.835024853531731
TCGA-B6-A0RV-01A-11R-A084-07	297.866058364002	883.134376094976	1181.00043445898	0.712076936465999
TCGA-E9-A22D-01A-11R-A157-07	763.378404690182	2571.84300848254	3335.22141317272	0.458438427695027
TCGA-A8-A07O-01A-11R-A00Z-07	-94.79585555	803.273212209063	708.477356661271	0.759024099527307
TCGA-AO-A0J2-01A-11R-A034-07	-42.36497237	1131.00172401645	1088.6367516438	0.721530233124732
TCGA-GM-A2DF-01A-11R-A180-07	724.11470512307	1733.20350321041	2457.31820833348	0.568847051592397

TCGA-E2-A1BC-01A-11R-A12P-07	1018.57044989911	-111.4939976	907.076452309926	0.739724482261718
TCGA-BH-A0BJ-01A-11R-A056-07	1052.03897568842	765.613033730673	1817.6520094191	0.643451677034751
TCGA-AO-A0JL-01A-11R-A056-07	131.59538593765	43.7095096732547	175.304895610905	0.807603157768423
TCGA-AC-A62V-01A-11R-A31O-07	365.171901454148	328.343619532645	693.515520986792	0.760452146939194
TCGA-D8-A1XG-01A-11R-A14D-07	115.509900406408	-23.0798023	92.4300981016137	0.814717279012446
TCGA-B6-A400-01A-11R-A239-07	570.866002405744	1037.88578375447	1608.75178616022	0.666618491485639
TCGA-EW-A3U0-01A-11R-A22K-07	1138.85710341255	2696.44471299502	3835.30181640756	0.392024585197796
TCGA-E2-A1B6-01A-31R-A12P-07	231.169311976051	2815.9532188544	3047.12253083045	0.495601200933455
TCGA-GM-A2DC-01A-11R-A18M-07	-111.646726	66.8242995463264	-44.82242644	0.826233598014519
TCGA-A8-A09E-01A-11R-A00Z-07	-57.64196515	-182.4608461	-240.1028113	0.842039689811827
TCGA-LL-A8F5-01A-11R-A36F-07	161.45285071097	1145.45661883714	1306.90946954812	0.698979832779528
TCGA-D8-A1JB-01A-11R-A13Q-07	882.104997946798	1911.50741149871	2793.61240944551	0.527571427385372
TCGA-E9-A3Q9-01A-11R-A21T-07	579.584401266011	1123.6791222874	1703.26352355341	0.656213582979808
TCGA-A7-A26H-01A-11R-A169-07	1139.06640012694	900.672396180643	2039.73879630759	0.618159527808225
TCGA-A7-A6VY-01A-12R-A33J-07	703.310106847048	1501.42530795819	2204.73541480524	0.598942222136641
TCGA-D8-A1X5-01A-11R-A14D-07	904.186031653336	837.979721472538	1742.16575312587	0.651893984081749
TCGA-E9-A1ND-01A-11R-A144-07	810.140628739744	2257.76174949406	3067.90237823381	0.492949612473181
TCGA-AR-A1AM-01A-41R-A22K-07	832.027684335696	754.214705870051	1586.24239020575	0.669077734880473
TCGA-B6-A0WW-01A-11R-A109-07	515.519671309403	5.13309358662083	520.652764896024	0.776683465751447
TCGA-AR-A1AR-01A-31R-A137-07	1207.03808543212	1888.55606037407	3095.59414580619	0.489408912240377
TCGA-EW-A2FV-01A-11R-A17B-07	800.401691456363	1146.39909185972	1946.80078331609	0.628825178362401
TCGA-A7-A56D-01A-11R-A27Q-07	921.566706097451	1111.49120349387	2033.05790959132	0.618930093949439

TCGA-BH-A1FJ-01A-11R-A13Q-07	-173.4384713	-483.9020876	-657.3405589	0.873477138613641
TCGA-XX-A899-01A-11R-A36F-07	1352.35488579391	1925.94040590283	3278.29529169674	0.465848615979359
TCGA-A8-A090-01A-11R-A00Z-07	835.959520096525	876.307476021205	1712.26699611773	0.655215759576774
TCGA-A8-A091-01A-11R-A00Z-07	329.243776563792	185.608274033903	514.852050597694	0.777219522274492
TCGA-AO-A12B-01A-11R-A10J-07	-320.0812981	15.3189756409448	-304.7623225	0.847121422814849
TCGA-EW-A1PE-01A-11R-A144-07	1111.44454530724	1493.31653395984	2604.76107926707	0.550915156758207
TCGA-GM-A5PX-01A-12R-A28M-07	1078.71711009237	614.165734884649	1692.88284497702	0.657362614640666
TCGA-BH-A18M-01A-11R-A12D-07	1170.18063803592	598.698186838289	1768.87882487421	0.648915517918818
TCGA-E2-A1LB-01A-11R-A144-07	551.137670385206	119.991396989737	671.129067374944	0.762581992481348
TCGA-E2-A10B-01A-11R-A10J-07	733.320411851216	523.311875628898	1256.63228748011	0.70423856962935
TCGA-PL-A8LX-01A-11R-A41B-07	-190.4856197	468.618733158497	278.13311346515	0.798610176974704
TCGA-E9-A249-01A-11R-A169-07	-44.70992613	190.560513992661	145.850587866382	0.810145313210594
TCGA-BH-A1FR-01A-11R-A13Q-07	178.903701927755	725.578250926137	904.481952853891	0.739980701235552
TCGA-E9-A1QZ-01A-21R-A169-07	973.289176545829	1849.33771898588	2822.62689553171	0.523948596056738
TCGA-C8-A138-01A-11R-A115-07	1229.81984890955	1752.71661146049	2982.53646037004	0.503813083491825
TCGA-AC-A2QI-01A-12R-A19W-07	1142.91025383735	1086.14613445688	2229.05638829423	0.596079553645126
TCGA-E9-A229-01A-31R-A157-07	1584.04894128756	1005.28374816419	2589.33268945175	0.552803780447457
TCGA-D8-A1Y0-01A-11R-A14M-07	1133.60378248754	1340.77841639727	2474.38219888481	0.566785216615496
TCGA-AR-A0TR-01A-11R-A084-07	-1051.208661	-37.5583046	-1088.766966	0.902537973642182
TCGA-D8-A1Y2-01A-11R-A157-07	1816.19529890568	2232.57480982593	4048.77010873161	0.363010383696297
TCGA-A2-A25F-01A-11R-A169-07	2129.60271581624	3286.35705089465	5415.95976671089	0.169979687247777
TCGA-A7-A0DC-01A-11R-A00Z-07	-774.5753318	-953.0949605	-1727.670292	0.93889681462918

TCGA-D8-A1XB-01A-11R-A14D-07	1022.26430744747	32.1192737693034	1054.38358121677	0.725002377442594
TCGA-D8-A1X7-01A-11R-A14M-07	565.928423670359	172.759338253238	738.687761923597	0.756129484945919
TCGA-S3-AA0Z-01A-11R-A41B-07	-37.53053426	1169.69024799492	1132.15971373975	0.717092135350756
TCGA-A2-A0EY-01A-11R-A034-07	501.073245644081	924.803747757743	1425.87699340182	0.686385338961385
TCGA-AO-A126-01A-11R-A10J-07	1190.6560817322	664.380304476845	1855.03638620904	0.639241330265426
TCGA-E9-A243-01A-21R-A169-07	1445.86686536281	2766.76184512047	4212.62871048328	0.340495744322871
TCGA-AN-A0FF-01A-11R-A034-07	764.885161651556	1015.88298787645	1780.76814952801	0.647586665310769
TCGA-D8-A1J9-01A-11R-A13Q-07	952.739162251909	585.451224154644	1538.19038640655	0.674303138173975
TCGA-BH-A0E7-01A-11R-A034-07	180.2193681 2112	-406.5768775	-226.3575094	0.84094962431186
TCGA-BH-A0C1-01B-11R-A12D-07	871.554690567333	888.261798542246	1759.81648910958	0.649927074890875
TCGA-D8-A1JF-01A-11R-A13Q-07	933.262460021923	589.599267148682	1522.8617271706	0.675963004033612
TCGA-A8-A08Z-01A-21R-A00Z-07	1644.87805880367	1241.51405029464	2886.39210909831	0.515953387652737
TCGA-A8-A096-01A-11R-A00Z-07	1525.46077674001	1803.50338635441	3328.96416309441	0.459254521841232
TCGA-C8-A274-01A-11R-A16F-07	-256.7066871	47.3928197709683	-209.3138673	0.839593231567636
TCGA-AC-A8OQ-01A-11R-A41B-07	1024.60543157294	1547.3052105472	2571.91064212014	0.554933043198807
TCGA-AC-A23C-01A-12R-A169-07	879.006651284423	941.727968198223	1820.73461948265	0.643105235010656
TCGA-BH-A18Q-01A-12R-A12D-07	146.542716650185	857.280709406578	1003.82342605676	0.730093994258023
TCGA-AR-A0TU-01A-31R-A109-07	-549.8747114	1239.68726400012	689.81255263276	0.760805014496464
TCGA-A2-A0SY-01A-31R-A084-07	1325.36527442755	1285.10365705197	2610.46893147951	0.55021572925032
TCGA-C8-A8HR-01A-11R-A36F-07	1529.94014198898	1588.75756662951	3118.69770861849	0.486448674825063
TCGA-BH-A5J0-01A-11R-A27Q-07	1279.33158728321	1892.05620085765	3171.38778814087	0.479676670630161
TCGA-AR-A24R-01A-11R-A169-07	506.049037077013	732.482621372108	1238.53165844912	0.706122427698692

TCGA-WT-AB41-01A-11R-A41B-07	376.566961754324	2262.38020386871	2638.94716562303	0.546720308607226
TCGA-AC-A3HN-01A-11R-A213-07	1535.48067862969	1345.01167198743	2880.49235061712	0.516695037907246
TCGA-LL-A73Y-01A-11R-A33J-07	850.28891485639	1160.76086809868	2011.04978295507	0.621464273003444
TCGA-A8-A07L-01A-11R-A00Z-07	372.555639678136	-269.9632966	102.592343100276	0.813851398311389
TCGA-E2-A1LG-01A-21R-A14M-07	-897.70982	292.133808709323	-605.5760113	0.869752545335588
TCGA-A2-A0CM-01A-31R-A034-07	815.990412431981	1753.40308223844	2569.39349467043	0.555240380576201
TCGA-S3-AA15-01A-11R-A41B-07	1486.07460558063	2484.8206535947	3970.89525917533	0.373637786070764
TCGA-BH-A1FB-01A-11R-A13Q-07	1462.61288470333	1781.88823856031	3244.50112326364	0.470232317667666
TCGA-BH-A0DK-01A-21R-A056-07	1503.14501128946	1959.68872162375	3462.83373291321	0.441711347339318
TCGA-C8-A27B-01A-11R-A169-07	3.78793582366302	1611.432847	1615.22078323566	0.665910379666419
TCGA-E2-A1IN-01A-11R-A13Q-07	1209.75921643482	182.28504870362	1392.04426513844	0.689988507150933
TCGA-E2-A156-01A-11R-A12D-07	-571.6919914	-295.935942	-867.6279334	0.888087083747161
TCGA-A7-A0DB-01A-11R-A00Z-07	1003.92792861796	803.89782901257	1807.82575763054	0.644555129899373
TCGA-B6-A1KF-01A-11R-A13Q-07	-60.64464372	-765.3753324	-826.0199761	0.885263040725598
TCGA-B6-A0WY-01A-11R-A109-07	814.606665202244	584.72886723634	1399.33553243858	0.68921342643081
TCGA-AO-A0JF-01A-11R-A056-07	1715.10587479998	1241.29773547459	2956.40361027458	0.507122953456251
TCGA-AC-A2FE-01A-11R-A19W-07	1260.55457392213	2058.79503120813	3319.34960513026	0.460507733472498
TCGA-AC-A2FO-01A-11R-A180-07	1268.66033968634	1666.19338546943	2934.85372515577	0.509846753704711
TCGA-B6-A40C-01A-11R-A239-07	927.626725378187	704.659287354773	1632.28601273296	0.66403949944433
TCGA-A2-A0CP-01A-11R-A034-07	1152.54732587036	481.416949749165	1633.96427561952	0.663855284374777
TCGA-A2-A3XZ-01A-42R-A239-07	257.92664946559	1899.17635334455	2157.10300281014	0.60452658419323
TCGA-E2-A2P6-01A-11R-A19W-07	219.551214949391	484.542590760579	704.09380570997	0.75944287143629

TCGA-E2-A14O-01A-31R-A115-07	7.39909477050306	231.623007155362	239.022101925865	0.802052235909317
TCGA-C8-A26W-01A-11R-A16F-07	797.72891987693	1128.61989030706	1926.34881018399	0.631156623805195
TCGA-BH-A0DS-01A-11R-A056-07	952.252363346947	1132.039949	2084.29231187094	0.613005608197938
TCGA-D8-A1JS-01A-11R-A13Q-07	-856.2090232	-235.7559573	-1091.964981	0.902740016189995
TCGA-D8-A1JE-01A-11R-A13Q-07	1539.24629833062	828.698325386138	2367.94462371675	0.579587429715088
TCGA-LL-A7SZ-01A-32R-A352-07	670.896200539784	2062.76762683483	2733.66382737461	0.535026391652005
TCGA-E2-A1IU-01A-11R-A14D-07	6.97368094621255	397.584973552877	404.55865449909	0.787304316072635
TCGA-A1-A0SI-01A-11R-A144-07	1440.29458973552	1164.87000492525	2605.16459466077	0.550865723577765
TCGA-BH-A1FH-01A-12R-A13Q-07	1736.55749303415	502.947003604398	2239.50449663855	0.594847437676833
TCGA-D8-A27F-01A-11R-A16F-07	597.248046373909	250.842211953835	848.090258327743	0.745523063986924
TCGA-AN-A0XO-01A-11R-A109-07	404.379145616399	-634.4041781	-230.0250325	0.841240810764513
TCGA-E2-A1II-01A-11R-A144-07	225.481654395072	2481.6992644268	2707.18091882188	0.53830654000406
TCGA-BH-AB28-01A-31R-A41B-07	655.335517713494	1013.31879588423	1668.65431359773	0.660038514475465
TCGA-AR-A2LM-01A-11R-A180-07	1263.81008651929	610.764480700932	1874.57456722022	0.637033211224084
TCGA-BH-A0HB-01A-11R-A056-07	631.98122270829	1293.10298849523	1925.08421120352	0.631300596650346
TCGA-AO-A0JD-01A-11R-A056-07	-541.4862715	556.005612996098	14.519341450711	0.821295345938118
TCGA-A1-A0SG-01A-11R-A144-07	787.910141204288	633.807210377793	1421.71735158208	0.686829253200482
TCGA-AC-A4ZE-01A-11R-A41B-07	65.4940919570682	17.886425927293	83.3805178843612	0.815486828435505
TCGA-A2-A4RW-01A-21R-A266-07	826.296078468352	624.206675	1450.50275351805	0.683752057769399
TCGA-D8-A27H-01A-11R-A16F-07	772.095096084208	369.065201429219	1141.16029751343	0.71617067496652
TCGA-AR-A0TT-01A-31R-A084-07	1402.78037229064	2552.53902008445	3955.31939237508	0.375757574123763
TCGA-A2-A3XV-01A-21R-A239-07	341.330762616503	531.012044325038	872.342806941541	0.743145681398655

TCGA-B6-A0I5-01A-11R-A034-07	454.159176317419	745.877538177558	1200.03671449498	0.710112273387033
TCGA-S3-A6ZG-01A-22R-A32P-07	277.402137846581	613.819981582464	891.222119429045	0.741288495154762
TCGA-LL-A5YL-01A-12R-A29R-07	601.143259881385	1214.6397542577	1815.78301413908	0.643661661673333
TCGA-AR-A2LK-01A-11R-A180-07	671.556497577109	476.501730047466	1148.05822762458	0.715463633505461
TCGA-A2-A04N-01A-11R-A115-07	995.139018789248	271.053743013337	1266.19276180259	0.703241537457329
TCGA-EW-A1P7-01A-21R-A144-07	1298.89336361882	2645.72616432503	3944.61952794385	0.377212628154264
TCGA-AO-A128-01A-11R-A10J-07	745.727647390584	3040.82384880405	3786.55149619463	0.398597670751244
TCGA-B6-A0IQ-01A-11R-A034-07	-496.502545	465.311451124712	-31.19109384	0.825104770369237
TCGA-E2-A155-01A-11R-A12D-07	-349.7286579	-513.5574902	-863.2861481	0.887793941472978
TCGA-E2-A574-01A-11R-A29R-07	-1037.885142	-682.184911	-1720.070053	0.938512233052383
TCGA-AC-A3W7-01A-11R-A22K-07	693.654020784937	260.137493589434	953.79151437437	0.735092829773864
TCGA-A2-A0EQ-01A-11R-A034-07	521.46247212924	2317.82436713179	2839.28683926103	0.521864088586862
TCGA-A2-A0SX-01A-12R-A084-07	989.295883826629	1991.7537177636	2981.04960159022	0.504001601740286
TCGA-A8-A09I-01A-22R-A034-07	691.73980961955	1145.81521539072	1837.55502501027	0.641212540330802
TCGA-AQ-A7U7-01A-22R-A352-07	1722.2272065638	2606.31664824759	4328.54385481139	0.324448943633253
TCGA-A7-A3IZ-01A-11R-A213-07	-227.1124226	-103.3794693	-330.4918919	0.84912236667656
TCGA-AN-A0FL-01A-11R-A034-07	-421.1513176	-184.9894703	-606.1407879	0.869793453818643
TCGA-EW-A423-01A-11R-A24H-07	0.68467975080236	1027.95976582418	1028.64444557498	0.727599422621847
TCGA-BH-A1FC-01A-11R-A13Q-07	91.1475931651483	1597.51547488893	1688.66306805408	0.657829263272762
TCGA-GM-A2DA-01A-11R-A18M-07	1141.31817005538	1607.47155897193	2748.78972902731	0.533149280669377
TCGA-EW-A3E8-01B-11R-A24H-07	1033.39679002167	1731.85897642801	2765.25576644967	0.531102872427104
TCGA-AO-A129-01A-21R-A10J-07	436.898090594561	1621.97450945081	2058.87260004537	0.615949368993488

TCGA-D8-A1XD-01A-11R-A14D-07	601.998858607663	-676.4272551	-74.42839648	0.828673904769901
TCGA-EW-A1IZ-01A-11R-A13Q-07	401.041758623944	2447.30059836462	2848.34235698856	0.520729743853319
TCGA-D8-A1JI-01A-11R-A13Q-07	-215.1037143	61.3918942821178	-153.71182	0.835131734431679
TCGA-AO-A0J5-01A-11R-A034-07	1404.46650620246	376.517602519262	1780.98410872172	0.647562509613136
TCGA-A2-A3XT-01A-11R-A22U-07	448.595874884316	1738.95176082194	2187.54763570625	0.600960685827106
TCGA-EW-A1J3-01A-11R-A13Q-07	310.892798527625	222.046089283561	532.938887811186	0.775546219695692
TCGA-D8-A1XJ-01A-11R-A14M-07	510.848530101154	92.9683769273048	603.816907028459	0.768936369963672
TCGA-XX-A89A-01A-11R-A36F-07	1836.61854801478	1750.4795983011	3587.09814631588	0.42527411093032
TCGA-B6-A0RQ-01A-11R-A115-07	52.8166475911433	-284.5106174	-231.6939698	0.841373236896955
TCGA-A2-A1FV-01A-11R-A13Q-07	253.384964472984	-554.6897462	-301.3047817	0.846851614258558
TCGA-BH-A0H5-01A-21R-A115-07	952.97545445439	3059.94745774611	4012.9229122005	0.36790834455942
TCGA-AN-A0FD-01A-11R-A034-07	1080.17230058309	628.431993838386	1708.60429442148	0.655621822189634
TCGA-A8-A09R-01A-11R-A00Z-07	908.324240921229	1088.94945323474	1997.27369415596	0.623047255241746
TCGA-AR-A24P-01A-11R-A169-07	1041.46836405236	413.643751384566	1455.11211543692	0.683258175900381
TCGA-AN-A0XP-01A-11R-A109-07	617.627638835609	117.136080346527	734.763719182136	0.756506309172232
TCGA-AR-A256-01A-11R-A169-07	68.412997225871	441.197897590758	509.61089481663	0.77770338421695
TCGA-EW-A2FS-01A-11R-A17B-07	285.920755748851	851.843519798715	1137.76427554757	0.716518499321694
TCGA-A2-A0CS-01A-11R-A115-07	764.179060728238	553.750232491701	1317.92929321994	0.697822118790932
TCGA-D8-A27N-01A-11R-A16F-07	1314.78817318296	1207.20764049274	2521.9958136757	0.561013319696679
TCGA-BH-A0B8-01A-21R-A056-07	390.312330013579	116.383297138508	506.695627152086	0.77797232165294
TCGA-A8-A08B-01A-11R-A00Z-07	-368.7351043	746.215202667788	377.480098383638	0.789748822297686
TCGA-E2-A159-01A-11R-A115-07	682.220940136371	1839.06251185165	2521.28345198802	0.561099877430462

TCGA-AN-A0FY-01A-11R-A034-07	601.420535482953	34.4049429885791	635.825478471532	0.765924031920409
TCGA-AR-A0TY-01A-12R-A115-07	-202.7120468	-137.4620702	-340.174117	0.849872201502889
TCGA-AR-A2LE-01A-11R-A180-07	313.212634054653	269.247988802266	582.460622856919	0.770936780417629
TCGA-OL-A6VR-01A-32R-A33J-07	-693.7877108	-362.0457889	-1055.8335	0.900445757564705
TCGA-BH-A0HI-01A-11R-A084-07	865.096831570818	533.765026213328	1398.86185778415	0.689263803269178
TCGA-BH-A208-01A-11R-A157-07	2133.04218938119	802.022893058587	2935.06508243978	0.50982006390435
TCGA-EW-A1P6-01A-11R-A144-07	731.722846127459	30.1092072881361	761.832053415595	0.753901848871283
TCGA-E2-A14W-01A-11R-A12D-07	-320.8730504	-613.8479811	-934.7210315	0.892571048306674
TCGA-S3-A6ZH-01A-22R-A32P-07	-310.2173724	240.191144235777	-70.02622814	0.828312040308827
TCGA-E2-A1L8-01A-11R-A13Q-07	863.212084393563	506.257391103469	1369.46947549703	0.692383250370348
TCGA-EW-A1IX-01A-12R-A144-07	1283.07844161338	607.352224716709	1890.43066633009	0.635237372367546
TCGA-AR-A0U0-01A-11R-A109-07	296.947770227657	1832.23054510503	2129.17831533269	0.607786714304295
TCGA-AR-A255-01A-11R-A169-07	1284.46043764715	1008.54796046786	2293.00839811501	0.588516007916112
TCGA-E2-A2P5-01A-11R-A19W-07	518.596020985011	341.490609329194	860.086630314205	0.744348285552456
TCGA-EW-A1PC-01B-11R-A21T-07	-214.0320361	-279.9857044	-494.0177405	0.861555199790944
TCGA-AC-A3OD-01A-11R-A21T-07	948.398755716334	1611.69444505503	2560.09320077137	0.556375265322803
TCGA-A8-A08X-01A-21R-A00Z-07	1226.64848058232	1409.78074516039	2636.42922574271	0.547029746646391
TCGA-A1-A0SH-01A-11R-A084-07	1597.62781482971	466.96686345552	2064.59467828523	0.615287463210034
TCGA-PL-A8LY-01A-11R-A41B-07	-430.4549872	334.757510202648	-95.69747697	0.830417376011824
TCGA-E2-A158-01A-11R-A12D-07	-1010.212344	-341.2574313	-1351.469775	0.918467976709429
TCGA-AC-A23G-01A-11R-A213-07	1821.41861780459	1149.63119459988	2971.04981240447	0.505268846999526
TCGA-AR-A5QM-01A-11R-A27Q-07	1082.29588330304	1154.66927843996	2236.96516174301	0.59514702309711

TCGA-GM-A2DL-01A-11R-A18M-07	1297.10283191807	867.833554074651	2164.93638599272	0.603610231704591
TCGA-A2-A3XU-01A-12R-A22U-07	-997.3644613	-611.4799626	-1608.844424	0.932750697676912
TCGA-A7-A425-01A-11R-A24H-07	894.756589917519	1022.34262758222	1917.09921749974	0.632209174611056
TCGA-C8-A12Q-01A-11R-A115-07	879.061225935881	832.624604376753	1711.68583031263	0.655280202722494
TCGA-E9-A1N9-01A-11R-A14D-07	513.493442730735	304.175108042995	817.66855077373	0.748491822319592
TCGA-A7-A4SB-01A-21R-A266-07	213.519338910439	-368.2054519	-154.686113	0.835210391240601
TCGA-A8-A0A7-01A-11R-A00Z-07	1639.54663195831	2746.84616088921	4386.39279284752	0.316405155502565
TCGA-BH-A0DQ-01A-11R-A084-07	1478.72145358074	619.732156310795	2098.45360989153	0.611361937391367
TCGA-B6-A408-01A-12R-A24H-07	838.873198273896	1399.25244637598	2238.12564464988	0.595010122007941
TCGA-AO-A12A-01A-21R-A115-07	889.588593318449	700.626171800794	1590.21476511924	0.668644266727742
TCGA-BH-A5IZ-01A-11R-A27Q-07	-231.1350699	337.738193056105	106.603123173923	0.813509158663302
TCGA-C8-A12U-01A-11R-A115-07	-626.301657	959.835083618998	333.533426633093	0.793689517083675
TCGA-A8-A081-01A-11R-A00Z-07	-194.0000488	1221.94330745266	1027.94325866444	0.727670026306604
TCGA-AR-A1AQ-01A-11R-A12P-07	646.090394980493	2000.59509659027	2646.68549157076	0.545768852605226
TCGA-AR-A24Z-01A-11R-A169-07	39.315116430945	169.653739871243	208.968856302188	0.804679189321831
TCGA-D8-A27M-01A-11R-A16F-07	1270.52833774347	2044.49954232692	3315.0278800704	0.461070750997062
TCGA-A2-A0YF-01A-21R-A109-07	317.966372590175	676.00670978504	993.973082375216	0.731081292825486
TCGA-A7-A26I-01B-06R-A22O-07	1545.99165089513	763.760623550169	2309.7522744453	0.586527131027686
TCGA-AR-A250-01A-31R-A169-07	1398.66555855747	1005.49609289444	2404.16165145191	0.575247002762634
TCGA-E2-A1IG-01A-11R-A144-07	1036.90219696164	298.925140534707	1335.82733749635	0.695937907117511
TCGA-OL-A5RW-01A-11R-A28M-07	-80.72025025	619.343221315356	538.622971070172	0.775019227610769
TCGA-AN-A03Y-01A-21R-A00Z-07	209.399524455566	585.416135341877	794.815659797443	0.750712149466427

TCGA-E2-A1B0-01A-11R-A12P-07	791.338619085939	872.51787617046	1663.8564952564	0.660567416289883
TCGA-C8-A1HJ-01A-11R-A13Q-07	-114.3463676	185.797286233955	71.450918607225	0.816499087110662
TCGA-C8-A12P-01A-11R-A115-07	962.8497392	700.02272180971	1662.87246098121	0.660675853761685
TCGA-LL-A740-01A-21R-A32P-07	1163.44580102067	746.141138513718	1909.58693953438	0.633063171369728
TCGA-EW-A1OV-01A-11R-A144-07	1073.48477300848	2653.03221376549	3726.51698677396	0.406664141415961
TCGA-E9-A1NH-01A-11R-A14D-07	1329.98620264671	726.29683938309	2056.2830420298	0.616248775301406
TCGA-A7-A26I-01A-11R-A169-07	1010.95007121593	262.133050823008	1273.08312203894	0.70252210428486
TCGA-E2-A1IJ-01A-11R-A144-07	1645.77047460502	1550.1685585054	3195.93903311042	0.476511391779992
TCGA-BH-A1F6-01A-11R-A13Q-07	575.86016497396	1324.1470794147	1900.00724438866	0.634151075530258
TCGA-AR-A2LN-01A-21R-A18M-07	1519.96518955306	956.141346892176	2476.10653644524	0.566576667574273
TCGA-A8-A0AD-01A-11R-A056-07	1099.97466761642	871.940154619141	1971.91482223556	0.625954525248968
TCGA-BH-A203-01A-12R-A169-07	495.318570423817	1103.64511882297	1598.96368924678	0.667688776862131
TCGA-C8-A1HO-01A-11R-A13Q-07	19.6896515235332	-149.7341908	-130.0445393	0.833215779672931
TCGA-A2-A0YL-01A-21R-A109-07	1034.03916494412	773.072527411103	1807.11169235522	0.64463526463437
TCGA-A2-A04T-01A-21R-A034-07	-118.5268917	1195.48135992341	1076.95446819968	0.722716486325464
TCGA-B6-A0IC-01A-11R-A034-07	-1384.079794	-425.3988078	-1809.478601	0.942962333880438
TCGA-A2-A04Q-01A-21R-A034-07	1144.8136385115	2751.55755987941	3896.37119839091	0.383762215542729
TCGA-E2-A1IL-01A-11R-A14D-07	752.543057751324	-120.4436129	632.099444896338	0.766275561109434
TCGA-LL-A5YO-01A-21R-A28M-07	889.142648255033	2816.52153580547	3705.66418406051	0.409458647298693
TCGA-A2-A25A-01A-12R-A16F-07	1483.32878966791	1159.107923	2642.43671278191	0.546291343038998
TCGA-A7-A0CE-01A-11R-A00Z-07	-214.4322016	38.5750951819577	-175.8571064	0.836915350545628
TCGA-EW-A2FR-01A-11R-A21T-07	1713.52940806138	841.782052548133	2555.31146060951	0.556958362102995

TCGA-A2-A1FX-01A-11R-A13Q-07	847.393823259439	676.052774098894	1523.44659735833	0.675899734033789
TCGA-E2-A14Y-01A-21R-A12D-07	-430.3732294	13.6602692290274	-416.7129602	0.855739166926136
TCGA-BH-A0H6-01A-21R-A056-07	769.22464209082	-96.50816875	672.716473343777	0.762431237775698
TCGA-E2-A105-01A-11R-A10J-07	-339.7451094	-296.0166561	-635.7617655	0.871930605852639
TCGA-LD-A7W5-01A-22R-A352-07	806.447303378113	1956.13576159501	2762.58306497312	0.531435248497131
TCGA-A2-A0T4-01A-31R-A084-07	1340.87879336982	1274.36071770483	2615.23951107465	0.549630856707358
TCGA-E9-A1R4-01A-21R-A14D-07	745.457768123766	1734.87201768837	2480.32978581213	0.566065735750086
TCGA-BH-A0B1-01A-12R-A056-07	775.427487119295	251.090857690795	1026.51834481009	0.727813479519212
TCGA-C8-A12N-01A-11R-A115-07	1195.68223151352	810.89832894773	2006.58056046125	0.62197810103612
TCGA-A2-A25E-01A-11R-A169-07	297.718888835797	48.6501687981376	346.369057633935	0.792541954288369
TCGA-BH-A0BQ-01A-21R-A115-07	1255.94103401118	1123.36747675672	2379.3085107679	0.578227283272516
TCGA-C8-A133-01A-32R-A12D-07	-1010.508925	-103.7676784	-1114.276603	0.90414407071017
TCGA-A8-A07C-01A-11R-A034-07	212.547641703045	883.910193595677	1096.45783529872	0.720734871493132
TCGA-GM-A3NY-01A-11R-A21T-07	281.335852337497	366.256701347652	647.592553685149	0.76481237425657
TCGA-B6-A0IA-01A-11R-A034-07	-708.9409054	-246.3859123	-955.3268177	0.893930816672617
TCGA-C8-A1HG-01A-11R-A137-07	617.394277437391	1488.39315711523	2105.78743455262	0.610509677698059
TCGA-A1-A0SN-01A-11R-A144-07	997.176208500575	1171.47019752099	2168.64640602156	0.603175953416363
TCGA-A8-A09T-01A-11R-A00Z-07	698.097862530262	405.32045526026	1103.41831779052	0.720026229389284
TCGA-AO-A03L-01A-41R-A056-07	901.51321342007	776.172402914422	1677.68561633449	0.659042033979776
TCGA-V7-A7HQ-01A-11R-A33J-07	341.109629559349	486.4588541	827.568483628149	0.747527354558821
TCGA-BH-A0BO-01A-23R-A12D-07	1458.44506867656	1230.46640703582	2688.91147571238	0.540564637581819
TCGA-B6-A0RN-01A-12R-A084-07	799.132106723109	-104.9393879	694.192718865356	0.760387590308652

TCGA-AR-A1AL-01A-21R-A12P-07	1124.62064296497	694.601287946958	1819.22193091193	0.643275256392962
TCGA-BH-A1F0-01A-11R-A137-07	1525.6740696115	2485.06658421575	4010.74065382725	0.36820618859201
TCGA-E9-A1R7-01A-11R-A14M-07	-151.3458872	-488.8492768	-640.195164	0.87224905808861
TCGA-E2-A15J-01A-11R-A12P-07	-155.7221061	-110.9450663	-266.6671723	0.844136647696059
TCGA-B6-A1KN-01A-11R-A13Q-07	460.964793273578	294.858152175154	755.822945448731	0.754481062346714
TCGA-BH-A0DI-01A-21R-A12P-07	1643.08034483283	1168.58103624212	2811.66138107495	0.52531890213886
TCGA-AR-A24U-01A-11R-A169-07	1397.70538670463	1930.19754611696	3327.90293282158	0.459392893086262
TCGA-A8-A0AB-01A-11R-A034-07	-85.07344661	-736.5418566	-821.6153033	0.884962149054628
TCGA-E2-A15G-01A-11R-A12D-07	-11.46712483	4.53788695291375	-6.929237874	0.823087446383455
TCGA-BH-A0HK-01A-11R-A056-07	888.992147025078	585.195727779903	1474.18787480498	0.681210931606171
TCGA-A8-A06Z-01A-11R-A00Z-07	-485.5650948	-98.36622825	-583.9313231	0.868180253047859
TCGA-E2-A1LL-01A-11R-A144-07	-316.4411146	-78.55829577	-394.9994104	0.85408569169529
TCGA-E2-A1L6-01A-11R-A13Q-07	635.572615147935	464.199592694708	1099.77220784264	0.720397531212279
TCGA-A2-A1G0-01A-11R-A13Q-07	1117.94411291734	879.396760235249	1997.34087315259	0.623039542016881
TCGA-BH-A1FL-01A-11R-A13Q-07	302.968894450607	-59.74415706	243.224737386647	0.801683638505055
TCGA-B6-A0IN-01A-11R-A034-07	1131.32939487808	1559.40203620216	2690.73143108024	0.540339865735332
TCGA-B6-A0IP-01A-11R-A034-07	204.341108545443	423.56000075932	627.901109304762	0.7666713745393
TCGA-B6-A0IB-01A-11R-A034-07	-514.6995797	-656.9439831	-1171.643563	0.90770957272049
TCGA-B6-A1KI-01A-11R-A14M-07	755.94273126565	-312.3075009	443.635230349116	0.783754776519438
TCGA-E2-A14U-01A-11R-A22K-07	-357.6441178	-79.53440983	-437.1785277	0.857289651050151
TCGA-BH-A0E0-01A-11R-A056-07	-524.0524523	395.499139941983	-128.5533124	0.833094722270107
TCGA-E2-A154-01A-11R-A115-07	432.510756537758	-257.6750175	174.835738991106	0.807643768388516

TCGA-BH-A0EA-01A-11R-A115-07	1015.28862669088	569.61045653675	1584.89908322763	0.669224265954034
TCGA-A2-A04U-01A-11R-A115-07	-43.0798652	-188.5295459	-231.6094111	0.841366528585437
TCGA-Z7-A8R5-01A-42R-A41B-07	727.267180722776	1622.00329723673	2349.2704779595	0.581819038511895
TCGA-BH-A18U-01A-21R-A12D-07	636.84091216158	579.848327768552	1216.68923993013	0.708389081497064
TCGA-BH-A0HW-01A-11R-A034-07	-1002.046735	-944.2811386	-1946.327874	0.949458882891093
TCGA-D8-A27T-01A-11R-A16F-07	1382.00733319949	1253.34827159857	2635.35560479807	0.547161664786777
TCGA-A2-A0ST-01A-12R-A084-07	789.974076808824	3005.51123523569	3795.48531204451	0.39739462731909
TCGA-B6-A1KC-01B-11R-A157-07	-142.7361901	-242.3136928	-385.0498829	0.853325140721885
TCGA-BH-A0HF-01A-11R-A056-07	1227.5977471346	1112.57896752279	2340.17671465739	0.582904185015513
TCGA-LL-A6FR-01A-12R-A31O-07	499.890198028844	316.584892121758	816.475090150602	0.748607984497962
TCGA-AN-A0FX-01A-11R-A034-07	446.704666538423	-108.1134774	338.591189174288	0.793237666649613
TCGA-D8-A13Y-01A-11R-A115-07	-771.0955168	-897.1098308	-1668.205348	0.935856655528003
TCGA-A2-A3Y0-01A-11R-A239-07	-194.0348418	1458.77296662282	1264.73812477996	0.703393326415649
TCGA-B6-A0WS-01A-11R-A115-07	1044.16820632079	1463.08395140025	2507.25215772104	0.562803541964853
TCGA-B6-A0RM-01A-11R-A084-07	-240.2696319	-289.3791091	-529.648741	0.864198596368554
TCGA-E9-A54Y-01A-11R-A466-07	-987.6045826	180.87471484512	-806.7298678	0.883942559459696
TCGA-B6-A0X1-01A-11R-A109-07	-461.8549703	1138.22048028855	676.365509967891	0.762084534676894
TCGA-A7-A0D9-01A-31R-A056-07	228.904113367136	-368.6761279	-139.7720145	0.834004473656775
TCGA-BH-A0B3-01A-11R-A056-07	769.305488109959	1391.95820010674	2161.2636882167	0.604039964893853
TCGA-AC-A3W6-01A-12R-A22K-07	1614.77422991121	1686.06560645811	3300.83983636932	0.462917808410369
TCGA-E2-A15K-01A-11R-A12P-07	510.152408243467	1287.87127291292	1798.02368115639	0.645654531938811
TCGA-GM-A2D9-01A-11R-A18M-07	654.526370591538	699.766379268883	1354.29274986042	0.693988931667498

TCGA-5L-AAT1-01A-12R-A41B-07	1118.99243577271	2056.71508308498	3175.70751885769	0.479120198126611
TCGA-E9-A22H-01A-11R-A157-07	68.5317045183969	541.247178790895	609.778883309291	0.768376570692101
TCGA-E2-A15E-06A-11R-A12D-07	-371.7978046	648.606671787164	276.808867222433	0.798727151624451
TCGA-AO-A03M-01B-11R-A10J-07	467.407825547163	1557.91162462568	2025.31945017284	0.619821895278529
TCGA-BH-A0H9-01A-11R-A056-07	115.009643766997	-2.69350792	112.316135847326	0.813021180753742
TCGA-A7-A0CD-01A-11R-A00Z-07	202.598025170506	-221.3003006	-18.70227545	0.824067655895843
TCGA-E2-A15E-01A-11R-A12D-07	390.759955534633	-340.5572512	50.2027042972445	0.818295852157952
TCGA-E2-A15S-01A-11R-A115-07	-723.666124	-738.078085	-1461.744209	0.924749257991611
TCGA-S3-AA11-01A-31R-A41B-07	-256.6310546	-483.4078693	-740.0389239	0.879322771255988
TCGA-D8-A1XS-01A-11R-A14M-07	936.124429763217	419.313622024289	1355.43805178751	0.693867880003307
TCGA-E2-A1BD-01A-11R-A12P-07	496.714840987637	107.039704517712	603.754545505349	0.768942222282275
TCGA-AN-A0FS-01A-11R-A034-07	1017.85895287927	46.6732054791306	1064.5321583584	0.723975552452492
TCGA-BH-A0DG-01A-21R-A12P-07	1502.44997309425	1232.98416032582	2735.43413342007	0.534806834346126
TCGA-A2-A0YT-01A-11R-A109-07	-0.333099384	224.253947870598	223.920848486364	0.803374190330215
TCGA-A2-A0T2-01A-11R-A084-07	292.422995367241	587.532217027617	879.955212394858	0.74239752355087
TCGA-S3-AA10-01A-21R-A41B-07	438.886891502091	2330.61520777597	2769.50209927806	0.530574632112731
TCGA-E2-A1LK-01A-21R-A14D-07	-896.423569	191.982058233067	-704.4415108	0.876822359147003
TCGA-B6-A0IH-01A-11R-A115-07	1097.48745564547	1060.36384974421	2157.85130538968	0.604439081950389
TCGA-A8-A0A4-01A-11R-A00Z-07	729.197763029357	-96.42902875	632.768734281313	0.766212434508116
TCGA-D8-A143-01A-11R-A115-07	-205.6771891	632.837068237689	427.159879139817	0.785254473854206
TCGA-D8-A140-01A-11R-A115-07	483.827019329999	749.252780787709	1233.07980011771	0.706688864213497
TCGA-A8-A076-01A-21R-A00Z-07	1007.09557216901	678.056771716447	1685.15234388546	0.658217308199919

TCGA-BH-A8FZ-01A-11R-A352-07	1241.48505969205	1794.82398489032	3036.30904458237	0.496979220840584
TCGA-E9-A1RE-01A-11R-A157-07	48.5481824228014	-334.1904021	-285.6422197	0.845626657943562
TCGA-A8-A08S-01A-11R-A034-07	222.127213884716	291.867143058144	513.99435694286	0.777298735786008
TCGA-A2-A0T6-01A-11R-A084-07	1766.1918649611	1442.83443823919	3209.02630320029	0.474821578991238
TCGA-AC-A62Y-01A-11R-A29R-07	780.436226104585	1329.8649885347	2110.30121463929	0.609984782020441
TCGA-AC-A3BB-01A-21R-A19W-07	1368.72133211735	1836.84274181128	3205.56407392863	0.475268788651171
TCGA-AR-A24S-01A-11R-A169-07	1584.2540889206	1650.27837047382	3234.53245939442	0.471523224540583
TCGA-BH-A0W4-01A-11R-A109-07	667.015440876271	-196.3027731	470.712667773492	0.781280052398005
TCGA-EW-A6SA-01A-21R-A32P-07	-723.4413972	-943.7399238	-1667.181321	0.93580367674011
TCGA-E9-A1N8-01A-11R-A144-07	-550.5793009	-416.8730522	-967.452353	0.894727154882629
TCGA-PE-A5DD-01A-12R-A27Q-07	804.560564577292	928.064522488288	1732.62508706558	0.652955326767978
TCGA-BH-A18L-01A-32R-A12D-07	-493.894271	-148.5303925	-642.4246635	0.872409064069945
TCGA-E9-A5FK-01A-11R-A27Q-07	927.75813749642	2430.92821282674	3358.68635032316	0.455374603182618
TCGA-A7-A0CH-01A-21R-A00Z-07	488.626336925984	49.9375408137987	538.563877739783	0.775024709144942
TCGA-AR-A24T-01A-11R-A169-07	1610.46769635161	765.425408364206	2375.89310471582	0.578636243350958
TCGA-A8-A07B-01A-11R-A00Z-07	699.58747070001	74.686447290609	774.273917990619	0.75270072270719
TCGA-AO-A1KR-01A-12R-A144-07	-756.8465967	503.24027615583	-253.6063205	0.843107242119326
TCGA-AO-A1KS-01A-11R-A13Q-07	383.792426291514	265.523322249059	649.315748540573	0.764649389185848
TCGA-BH-A2L8-01A-11R-A18M-07	1091.06635280721	2098.60047484831	3189.66682765552	0.477320628528917
TCGA-LD-A7W6-01A-81R-A352-07	405.688738940822	477.804840967175	883.493579907997	0.742049452217776
TCGA-A8-A09Q-01A-11R-A00Z-07	1034.18366500234	1499.69643783503	2533.88010283737	0.559568377915589
TCGA-B6-A0RO-01A-22R-A084-07	-245.6786446	-78.12613413	-323.8047788	0.848603485260125

TCGA-HN-A2NL-01A-11R-A18M-07	-668.8185578	652.816366031312	-16.00219176	0.823843067431732
TCGA-D8-A27R-01A-11R-A16F-07	795.793467040104	942.441694050153	1738.23516109026	0.652331394189559
TCGA-BH-A42T-01A-11R-A24H-07	536.351744332527	860.811306661024	1397.16305099355	0.689444449483114
TCGA-AR-A24O-01A-11R-A169-07	1707.62579092759	1204.35094425423	2911.97673518183	0.51273271030339
TCGA-BH-A0DP-01A-21R-A056-07	1077.66494933634	1065.81512469659	2143.48007403293	0.606118295789916
TCGA-AO-A03O-01A-11R-A00Z-07	-23.09135028	949.136984894984	926.045634613765	0.737847929205611
TCGA-A1-A0SP-01A-11R-A084-07	140.722070997626	1116.03434741008	1256.75641840771	0.704225633283256
TCGA-AR-A0TP-01A-11R-A084-07	-451.644461	-586.3825462	-1038.027007	0.899305768652558
TCGA-E9-A1NF-01A-11R-A14D-07	1841.85373336141	934.942388149494	2776.79612151091	0.529666780699245
TCGA-AR-A24M-01A-11R-A169-07	1073.96427465523	353.349010719123	1427.31328537435	0.686231999384126
TCGA-A2-A0EX-01A-21R-A034-07	1117.29060969006	591.155311656511	1708.44592134657	0.655639375825509
TCGA-D8-A27V-01A-12R-A17B-07	819.166566950388	136.804676936119	955.971243886506	0.73487587067225
TCGA-AN-A0XV-01A-11R-A109-07	932.301155387793	631.569311749243	1563.87046713704	0.67151472118852
TCGA-A8-A083-01A-21R-A00Z-07	-637.8619254	-130.5178558	-768.3797813	0.881296309808881
TCGA-A2-A0CV-01A-31R-A115-07	1059.24723325578	1057.88304177196	2117.13027502774	0.609190139484641
TCGA-A2-A4S2-01A-12R-A266-07	773.555424319034	1406.64351517344	2180.19893949247	0.601822520513801
TCGA-AO-A124-01A-11R-A10J-07	-594.3863887	237.83755825612	-356.5488304	0.851136425130748
TCGA-E9-A1NA-01A-11R-A144-07	1065.12219161698	1070.21418193889	2135.33637355587	0.607068653793631
TCGA-A8-A09A-01A-11R-A00Z-07	1043.23628895615	595.633790693453	1638.87007964961	0.663316566236597
TCGA-E9-A2JT-01A-22R-A18M-07	1123.74667778344	3139.87977978507	4263.62645756851	0.333447702702623
TCGA-OL-A5RU-01A-11R-A28M-07	528.151286214916	1666.49571821742	2194.64700443234	0.600127427472059
TCGA-A2-A0D0-01A-11R-A00Z-07	-751.3683137	985.001651155455	233.63333744169	0.802524417616319

TCGA-E2-A15R-01A-11R-A115-07	-363.874514	-805.7544432	-1169.628957	0.907585448907249
TCGA-OL-A6VO-01A-12R-A33J-07	-327.0925616	551.773462593253	224.680901031585	0.803307750085434
TCGA-AR-A1AU-01A-11R-A12P-07	702.350691952532	165.932350093767	868.283042046299	0.743544301441479
TCGA-AO-A0J3-01A-11R-A034-07	-792.4956632	-354.6065141	-1147.102177	0.90619212664309
TCGA-D8-A1XQ-01A-11R-A14M-07	764.197906481267	1548.05003496363	2312.2479414449	0.586230386152975
TCGA-AR-A24V-01A-21R-A169-07	1624.87903342697	898.309155680726	2523.1881891077	0.56086842266582
TCGA-E9-A1NE-01A-21R-A14M-07	1210.86649712382	2410.08521062004	3620.95170774386	0.420771331909145
TCGA-AO-A1KP-01A-11R-A13Q-07	-373.7608067	-632.9545297	-1006.715336	0.897286269162347
TCGA-E9-A1RH-01A-21R-A169-07	379.839522751615	195.460549076553	575.300071828168	0.771605803270818
TCGA-BH-A18S-01A-11R-A12D-07	-157.8610759	188.804098075788	30.9430222178435	0.819917588044465
TCGA-BH-A0BA-01A-11R-A056-07	665.910698102852	528.179512552478	1194.09021065533	0.710726585960846
TCGA-E9-A1R6-01A-11R-A14D-07	303.974607941336	-58.45063691	245.523971027111	0.801481852215967
TCGA-OL-A5S0-01A-11R-A28M-07	886.841965889745	1305.872369	2192.71433444174	0.600354331030536
TCGA-BH-A1EO-01A-11R-A137-07	1851.64733007982	1155.73744883407	3007.38477891389	0.500659029915691
TCGA-B6-A0RP-01A-21R-A084-07	1104.12827043069	800.80804729746	1904.93631772815	0.633591468953578
TCGA-UL-AAZ6-01A-11R-A41B-07	-627.0483831	117.183819451644	-509.8645637	0.862733759979351
TCGA-B6-A0RT-01A-21R-A084-07	1093.35970125854	3120.02553371943	4213.38523497797	0.340391328820268
TCGA-BH-A0AZ-01A-21R-A12P-07	1762.87639082275	791.409007102369	2554.28539792512	0.557083446882411
TCGA-BH-A1ES-06A-12R-A24H-07	-639.9583973	-232.2949741	-872.2533713	0.888398980424672
TCGA-AR-A252-01A-11R-A169-07	1396.35395378213	2449.04738232075	3845.40133610287	0.390660329051793
TCGA-AQ-A54N-01A-11R-A266-07	-1292.318911	98.5032699715675	-1193.815641	0.909070391437245
TCGA-A7-A5ZW-01A-12R-A29R-07	1413.44571046316	813.317056423108	2226.76276688627	0.596349846378318

TCGA-BH-A209-01A-11R-A157-07	682.770320354612	2387.45679782795	3070.22711818256	0.492652681007022
TCGA-3C-AALI-01A-11R-A41B-07	379.822528411364	1174.89316310701	1554.71569151837	0.672509869573906
TCGA-E2-A1L9-01A-11R-A13Q-07	1531.43261405849	963.889856267815	2495.32247032631	0.564250154107815
TCGA-BH-A0B9-01A-11R-A056-07	-509.0521963	1719.82270426094	1210.77050793904	0.709002033199699
TCGA-GM-A2DH-01A-11R-A180-07	123.771387583652	1632.86400424337	1756.63539182702	0.650281883041521
TCGA-A2-A0CK-01A-11R-A22K-07	646.710153081892	750.75890824157	1397.46906132346	0.689411912392735
TCGA-A8-A07F-01A-11R-A00Z-07	584.054426774752	537.903019500279	1121.95744627503	0.718135107907852
TCGA-AR-A254-01A-21R-A169-07	1349.99138741209	1573.21638501859	2923.20777243067	0.511316623500571
TCGA-B6-A0IM-01A-11R-A034-07	515.981092939391	20.1128423176079	536.093935256999	0.77525377038791
TCGA-B6-A0X7-01A-11R-A10J-07	824.558198060098	-67.1451885	757.413009558087	0.754327854338283
TCGA-EW-A1OX-01A-11R-A144-07	-1136.166718	-1098.767752	-2234.93447	0.961900756130927
TCGA-GM-A4E0-01A-12R-A266-07	1673.00946751417	1958.9095703193	3631.91903783346	0.419310360568641
TCGA-E2-A14X-01A-11R-A115-07	255.945341333521	1301.75761234401	1557.70295367753	0.672185279654565
TCGA-B6-A0I1-01A-11R-A21T-07	-1015.905294	-438.9325732	-1454.837867	0.924362965046103
TCGA-A2-A0YM-01A-11R-A109-07	317.062415815125	1112.80110575644	1429.86352157156	0.685959659152091
TCGA-A8-A08R-01A-11R-A034-07	711.206936192756	1501.76366511744	2212.97060131019	0.597973763198258
TCGA-E9-A1N4-01A-11R-A14M-07	1148.75887546408	1440.41402053624	2589.17289600032	0.552823326303089
TCGA-D8-A1Y3-01A-11R-A157-07	298.035325389985	1290.7588774479	1588.79420283789	0.668799305535162
TCGA-LL-A441-01A-11R-A24H-07	552.513505904123	2270.99941766782	2823.51292357194	0.523837814195247
TCGA-D8-A1XT-01A-11R-A14M-07	1297.55407235482	943.903271315916	2241.45734367073	0.594616988646734
TCGA-A8-A06U-01A-11R-A00Z-07	93.6986675010301	700.747953838532	794.446621339562	0.750747935035292
TCGA-A2-A0EP-01A-52R-A22U-07	2005.63674572435	3037.67789934101	5043.31464506536	0.223602540007964

TCGA-D8-A27G-01A-11R-A16F-07	1141.77625599809	1035.72745970636	2177.50371570444	0.602138433411506
TCGA-A7-A26J-01A-11R-A169-07	878.040685430321	405.155053134365	1283.19573856469	0.701464929182506
TCGA-E2-A1B5-01A-21R-A12P-07	981.514529368024	2652.84990223918	3634.36443160721	0.418984458396806
TCGA-OL-A5D8-01A-11R-A27Q-07	178.880500333846	518.608553465659	697.489053799505	0.76007324654284
TCGA-A8-A06X-01A-21R-A00Z-07	446.456844096426	641.59178505578	1088.04862915221	0.721590003549391
TCGA-E2-A1LE-01A-12R-A19W-07	1336.31140749096	1531.95773040025	2868.26913789121	0.51823036686459
TCGA-A2-A04X-01A-21R-A034-07	470.933168706089	1752.42914748048	2223.36231618656	0.596750449445326
TCGA-AR-A2LL-01A-11R-A180-07	417.305789875538	771.032435724939	1188.33822560048	0.711320288249391
TCGA-A8-A0A9-01A-11R-A00Z-07	644.283676144607	503.647430031463	1147.93110617607	0.715476670157339
TCGA-BH-A1EX-01A-11R-A13Q-07	1207.4144299788	957.792998891023	2165.20742886982	0.603578510702162
TCGA-BH-A0AV-01A-31R-A115-07	1310.85583044262	644.285204639603	1955.14103508222	0.627872794073839
TCGA-BH-A1FM-01A-11R-A13Q-07	908.018382483003	82.3482009697572	990.36658345276	0.7314423895658
TCGA-AQ-A1H3-01A-31R-A13Q-07	976.65636768386	767.134916196384	1743.79128388024	0.651713025862779
TCGA-OL-A5RY-01A-21R-A28M-07	1447.830439	2374.85755594774	3822.68799464174	0.393727266135751
TCGA-E9-A245-01A-22R-A16F-07	-59.80037997	-182.4016885	-242.2020684	0.842205868905722
TCGA-C8-A12O-01A-11R-A115-07	1311.88837028327	915.499987076105	2227.38835735938	0.596276130121466
TCGA-E2-A1AZ-01A-11R-A12P-07	1100.53292837904	1531.88161314694	2632.41454152598	0.547522970015025
TCGA-BH-A0HY-01A-11R-A056-07	453.152887623951	-240.9606598	212.192227860559	0.804398183326966
TCGA-EW-A1PF-01A-11R-A144-07	1224.61992118467	784.08885030537	2008.70877149004	0.621733453260186
TCGA-BH-A0BV-01A-11R-A00Z-07	1107.04740318869	1128.94562119204	2235.99302438073	0.595261691944853
TCGA-PE-A5DE-01A-11R-A27Q-07	819.361086617823	2583.37077072758	3402.7318573454	0.449609007724468
TCGA-AR-A251-01A-12R-A169-07	317.446278575837	329.858988141784	647.305266717621	0.764839541982964

TCGA-E2-A1B1-01A-21R-A12P-07	1608.03422678803	1646.80779510163	3254.84202188966	0.468892143782117
TCGA-BH-A0E2-01A-11R-A056-07	864.468483691201	433.401947482827	1297.87043117403	0.699928085398356
TCGA-AR-A0TW-01A-11R-A084-07	311.473278989445	1256.00785320315	1567.48113219259	0.671121898771832
TCGA-A7-A13E-01A-11R-A12P-07	-39.43046842	-330.9816658	-370.4121343	0.852202909639254
TCGA-C8-A1HM-01A-12R-A137-07	153.445185119915	2176.6161946542	2330.06137977412	0.584110013973265
TCGA-E2-A1LS-01A-12R-A157-07	-192.777772	-214.8198775	-407.5976495	0.855046098623111
TCGA-A2-A0YK-01A-22R-A109-07	1398.62431558553	2122.09241318194	3520.71672876747	0.434072751946622
TCGA-LL-A50Y-01A-11R-A266-07	186.433974546259	1002.78207016202	1189.21604470828	0.711229715226566
TCGA-B6-A0RS-01A-11R-A084-07	543.89892015199	1169.07731583425	1712.97623598624	0.655137108351403
TCGA-PL-A8LZ-01A-31R-A36F-07	1293.21646126482	2092.94924546707	3386.16570673189	0.451779749374618
TCGA-A7-A2KD-01A-31R-A21T-07	1057.97412223533	1128.55764727454	2186.53176950987	0.601079865468322
TCGA-LD-A9QF-01A-32R-A41B-07	1546.38036070449	2686.04071191011	4232.4210726146	0.337762624468027
TCGA-AR-A1AK-01A-21R-A12P-07	1223.22547796972	1691.48666682927	2914.71214479899	0.51238793917502
TCGA-LL-A6FQ-01A-11R-A31O-07	145.629040213431	665.049076762593	810.678116976023	0.749171889908862
TCGA-E9-A1RB-01A-11R-A157-07	162.914670908657	217.92753064952	380.842201558176	0.789445987369669
TCGA-AN-A0AJ-01A-11R-A00Z-07	501.533343743135	1432.84602535409	1934.37936909722	0.630241848417939
TCGA-A2-A0D2-01A-21R-A034-07	365.66799373715	1177.19747727221	1542.86547100936	0.673796216267146
TCGA-EW-A6S9-01A-22R-A33J-07	759.787942491083	198.509454209325	958.297396700408	0.734644254364026
TCGA-A8-A093-01A-11R-A00Z-07	766.144400161107	-295.0071671	471.137233080768	0.781241151155095
TCGA-AC-A2FB-01A-11R-A17B-07	1041.64946312514	2385.96189007943	3427.61135320457	0.446343938180486
TCGA-C8-A137-01A-11R-A115-07	242.463412342645	-519.1911435	-276.7277311	0.844927466590324
TCGA-E9-A1N3-01A-12R-A157-07	-380.8180838	-537.4368996	-918.2549834	0.891478589267994

TCGA-AO-A03U-01B-21R-A10J-07	1226.23722195835	1972.06993331777	3198.30715527613	0.476205752787376
TCGA-A2-A0D1-01A-11R-A034-07	-146.3531393	-298.4764943	-444.8296336	0.857867317473375
TCGA-E2-A572-01A-13R-A31O-07	-472.2566742	225.525427696424	-246.7312465	0.842564130322154
TCGA-B6-A0RE-01A-11R-A056-07	-84.01506136	177.457903120184	93.4428417617144	0.814631068829125
TCGA-AR-A5QN-01A-12R-A28M-07	780.488945109207	740.429884852833	1520.91882996204	0.676173146709675
TCGA-D8-A1JC-01A-11R-A13Q-07	747.522904088238	716.607201613533	1464.13010570177	0.682291015876731
TCGA-A2-A0T3-01A-21R-A115-07	-110.4970313	-364.5372612	-475.0342925	0.860137225864469
TCGA-B6-A0I6-01A-11R-A034-07	430.919045680517	-8.82955341	422.089492270557	0.785715090793388
TCGA-B6-A0IK-01A-12R-A056-07	162.770675643014	471.971101927719	634.741777570733	0.766026296312041
TCGA-B6-A0IO-01A-11R-A034-07	-381.1213558	309.816041917482	-71.3053139	0.828417218621191
TCGA-B6-A0WZ-01A-11R-A109-07	128.591352398983	-211.9868279	-83.39547554	0.829409940911117
TCGA-BH-A204-01A-11R-A157-07	363.844318492778	-384.3521163	-20.50779777	0.824217764014923
TCGA-A1-A0SQ-01A-21R-A144-07	81.9510932379276	242.641880493363	324.59297373129	0.794487169057919
TCGA-AO-A0JC-01A-11R-A056-07	859.810125186697	3653.83155866625	4513.64168385295	0.298631962737275
TCGA-BH-A0DX-01A-11R-A115-07	1428.48590221885	1280.80648449717	2709.29238671602	0.538045313340375
TCGA-A2-A0T1-01A-21R-A084-07	1508.50518009705	2154.8061712613	3663.31135135835	0.415122558325273
TCGA-AO-A0J8-01A-21R-A034-07	657.34950218607	952.506998423914	1609.85650060998	0.666497609353682
TCGA-D8-A146-01A-31R-A115-07	1245.91892444517	712.500403347227	1958.4193277924	0.627498183803546
TCGA-AN-A0AT-01A-11R-A034-07	-327.0331984	571.5690034 4844	244.535805095871	0.801568587229187
TCGA-BH-A1EY-01A-11R-A13Q-07	1432.10515699127	1554.76627590525	2986.87143289652	0.503263317054247
TCGA-A8-A08F-01A-11R-A00Z-07	981.550019172616	1538.17296050785	2519.72297968046	0.561289466055452
TCGA-GM-A3XN-01A-12R-A22U-07	1326.76056769933	1165.60586255844	2492.36643025777	0.564608340731678

TCGA-E2-A15K-06A-11R-A12P-07	-1020.2986	-78.40752093	-1098.706121	0.903165252892208
TCGA-E9-A1R5-01A-11R-A14M-07	732.153507359633	-0.574138095	731.579369264716	0.756811916519888
TCGA-BH-A0B7-01A-12R-A115-07	1178.59864584865	1701.90116395768	2880.49980980633	0.516694100467715
TCGA-AO-A1KT-01A-11R-A13Q-07	895.795642227486	605.607400195468	1501.40304242295	0.678280909817497
TCGA-AR-A1AJ-01A-21R-A12P-07	1356.9873613104	2621.44421986093	3978.43158117133	0.37261143311966
TCGA-OL-A5RV-01A-12R-A28M-07	199.390960318613	7.32538682177415	206.716347140387	0.804875450877679
TCGA-EW-A1P3-01A-11R-A144-07	575.438086617013	479.826693909219	1055.26478052623	0.72491328214809
TCGA-BH-A0EI-01A-11R-A115-07	977.946722697555	-131.4703232	846.476399511291	0.74568092930725
TCGA-BH-A0H7-01A-13R-A056-07	940.853444076993	314.578161674865	1255.43160575186	0.70436368700763
TCGA-BH-A0GZ-01A-11R-A056-07	801.054686584299	212.60717458545	1013.66186116975	0.72910636566277
TCGA-D8-A1XZ-01A-11R-A14M-07	816.039406121776	807.018817301679	1623.05822342346	0.665051670461547
TCGA-A8-A06Q-01A-11R-A034-07	-873.3646883	-419.0607563	-1292.425445	0.915005756159471
TCGA-OL-A66K-01A-11R-A29R-07	759.27911044843	90.3754468233343	849.654557271765	0.745370006610133
TCGA-D8-A1JN-01A-11R-A13Q-07	-768.7088095	175.347138498477	-593.361671	0.868866361460413
TCGA-D8-A1XL-01A-11R-A14M-07	357.030526961295	1698.38953733921	2055.4200643005	0.616348533544235
TCGA-E2-A3DX-01A-21R-A213-07	1604.53509896011	1983.34921896765	3587.88431792776	0.42516966285377
TCGA-S3-AA12-01A-11R-A41B-07	-267.0534766	-227.807901	-494.8613775	0.86161806031504
TCGA-A8-A095-01A-11R-A00Z-07	434.068156613808	309.19520349488	743.263359663296	0.755689775357847
TCGA-A2-A0EM-01A-11R-A034-07	861.650199112767	184.575675189619	1046.22587430239	0.725826601432679
TCGA-AO-A03P-01A-11R-A00Z-07	231.914335815121	48.0113178229167	279.925653638038	0.798451788446886
TCGA-A2-A0CW-01A-21R-A115-07	-295.1629816	604.33005388121	309.167071667594	0.795860223751001
TCGA-BH-A0BC-01A-22R-A084-07	1241.21955991253	1554.22898759811	2795.44854751063	0.527342444906888

TCGA-A2-A0CU-01A-12R-A034-07	391.559901447914	-80.7752943	310.784607145159	0.795716438812844
TCGA-OL-A66I-01A-21R-A29R-07	1124.79074432362	2667.64583019394	3792.43657451756	0.397805252622155
TCGA-A7-A13H-01A-11R-A22K-07	879.53558947452	1609.41922491773	2488.95481439225	0.565021597829365
TCGA-E2-A15L-01A-11R-A12D-07	648.100100492848	-13.24791231	634.852188180305	0.766015878204262
TCGA-AQ-A54O-01A-11R-A266-07	-11.24866624	659.620434957438	648.371768717407	0.764738679761769
TCGA-A7-A26E-01B-06R-A277-07	1198.01905393604	463.561865044453	1661.5809189805	0.660818156695033
TCGA-A2-A0CT-01A-31R-A056-07	-9.646728729	11.6086710434934	1.96194231414375	0.822345547618222
TCGA-AO-A0JE-01A-11R-A056-07	1617.35571506406	1768.04318328991	3385.39889835397	0.451880163338496
TCGA-B6-A0IE-01A-11R-A034-07	223.815764997523	-9.469629391	214.346135606443	0.804210310310155
TCGA-A8-A092-01A-11R-A00Z-07	351.362036015763	238.778818538836	590.140854554599	0.770218256226837
TCGA-LD-A66U-01A-11R-A31O-07	815.137808200977	1459.22218998357	2274.35999818454	0.590726923373899
TCGA-UU-A93S-01A-21R-A41B-07	-622.9433711	436.398320592881	-186.5450505	0.837773011454209
TCGA-BH-A0BR-01A-21R-A115-07	1512.20214978081	1183.79279455566	2695.99494433647	0.539689583582182
TCGA-C8-A12Z-01A-11R-A115-07	65.4446816836857	-237.7481677	-172.303486	0.836629731591802
TCGA-BH-A1FE-01A-11R-A13Q-07	1308.28161796486	1563.99351572414	2872.27513368901	0.517727366713726
TCGA-D8-A145-01A-11R-A115-07	1004.12537278518	597.330410247057	1601.45578303224	0.667416408063105
TCGA-EW-A1PD-01A-11R-A144-07	968.358421204461	142.137865671725	1110.49628687619	0.719304855278981
TCGA-E2-A150-01A-11R-A12D-07	646.411965034455	1094.22238998456	1740.63435501902	0.652064429261697
TCGA-D8-A1JD-01A-11R-A13Q-07	1211.48620476722	841.659274048882	2053.14547881611	0.616611423007547
TCGA-A8-A06Y-01A-21R-A00Z-07	-675.0131405	-472.5924273	-1147.605568	0.906223370543045
TCGA-B6-A409-01A-11R-A24H-07	44.3701689294991	490.972802778253	535.342971707752	0.775323394175049
TCGA-E9-A1RA-01A-11R-A14D-07	527.933241355835	517.60939862879	1045.54263998463	0.725895585637932

TCGA-E9-A248-01A-11R-A169-07	544.613197564636	2077.60513418302	2622.21833174765	0.548774768589602
TCGA-BH-A18P-01A-11R-A12D-07	493.832379312111	408.829875915669	902.66225522778	0.740160340865649
TCGA-LQ-A4E4-01A-11R-A266-07	9.1487489508169	-445.7003258	-436.5515768	0.857242267668639
TCGA-E2-A15M-01A-11R-A12D-07	1769.84412425337	2043.25496489436	3813.09908914772	0.395020724978108
TCGA-A8-A07U-01A-11R-A034-07	983.298874744162	2375.16479649787	3358.46367124203	0.45540370391154
TCGA-C8-A1HK-01A-21R-A13Q-07	-129.734995	182.429878943616	52.6948839496764	0.818085523310525
TCGA-C8-A273-01A-11R-A16F-07	754.17816397418	234.252388545304	988.430552519485	0.731636147983486
TCGA-BH-A8FY-01A-11R-A36F-07	-544.5879017	253.259975243326	-291.3279264	0.846071849598453
TCGA-BH-A0B2-01A-11R-A10J-07	1616.50176025003	1848.12227438577	3464.62403463581	0.441475563185229
TCGA-AC-A62X-01A-11R-A29R-07	-685.0539834	-217.5377059	-902.5916893	0.890434556420136
TCGA-BH-A0E1-01A-11R-A056-07	407.408288000404	-111.4783678	295.929920209894	0.797035205533191
TCGA-A1-A0SO-01A-22R-A084-07	-974.5981933	-516.9918469	-1491.59004	0.926407694039477
TCGA-A7-A0CG-01A-12R-A056-07	1346.82653837034	1480.78875623971	2827.61529461006	0.523324771062106
TCGA-C8-A278-01A-11R-A169-07	1328.06637958792	1745.04982063669	3073.11620022462	0.492283588017718
TCGA-D8-A27E-01A-11R-A16F-07	126.722519701951	363.684739493814	490.407259195764	0.77947232285393
TCGA-AR-A24H-01A-11R-A169-07	-41.77504052	688.862205378404	647.087164856034	0.764860166206969
TCGA-C8-A12K-01A-21R-A115-07	680.486862085264	2415.24364093219	3095.73050301746	0.489391457400241
TCGA-A8-A09K-01A-11R-A00Z-07	-481.7165084	794.95192859437	313.235420155059	0.795498497224031
TCGA-BH-A18V-06A-11R-A213-07	407.821161759901	2527.47006049291	2935.29122225281	0.509791506850621
TCGA-E2-A9RU-01A-11R-A41B-07	415.675186720862	563.238683012622	978.913869733484	0.732587720374181
TCGA-E2-A15A-01A-11R-A12D-07	756.618529409251	951.760474202088	1708.37900361134	0.655646792696676
TCGA-AR-A0U4-01A-11R-A109-07	759.257682317129	2151.92001104885	2911.17769336598	0.512833405965201

TCGA-A2-A0EV-01A-11R-A034-07	1088.09525248001	1130.86621727324	2218.96146975325	0.597268687209752
TCGA-D8-A1XF-01A-11R-A14D-07	-695.8830611	-342.0056152	-1037.888676	0.899296888514765
TCGA-LL-A5YP-01A-21R-A28M-07	1283.08123033298	1022.03308557757	2305.11431591055	0.587078393914725
TCGA-E2-A1IO-01A-11R-A144-07	1418.14796520696	976.855225654618	2395.00319086158	0.576346138425518
TCGA-D8-A3Z5-01A-41R-A24H-07	90.6682079716084	13.6141311892857	104.282339160894	0.813707225420488
TCGA-D8-A1XR-01A-11R-A14M-07	275.332841422055	-25.01893066	250.313910762792	0.801061182374105
TCGA-A1-A0SM-01A-11R-A084-07	1017.36987013923	542.376602677471	1559.7464728167	0.671963160507988
TCGA-D8-A1JP-01A-11R-A13Q-07	1328.91598928765	914.575990907204	2243.49198019485	0.594376835936186
TCGA-AC-A6IX-01A-12R-A32P-07	1272.42197696621	1049.74266671573	2322.16464368195	0.585050473240875
TCGA-D8-A4Z1-01A-21R-A266-07	900.119247179838	519.23801155254	1419.35725873238	0.687081006910316
TCGA-B6-A402-01A-11R-A239-07	-294.6448382	-307.0859065	-601.7307448	0.869473862117117
TCGA-A2-A1FZ-01A-51R-A14D-07	1254.06946527894	895.988440678157	2150.0579059571	0.605350039971656
TCGA-E9-A3HO-01A-11R-A213-07	8.77787591253497	861.965240888978	870.743116801513	0.743302783229135
TCGA-AC-A2BM-01A-11R-A21T-07	246.381739954493	801.428694745517	1047.81043470001	0.725666584825464
TCGA-BH-A0E9-01B-11R-A115-07	533.812974035422	565.26374353385	1099.07671756927	0.720468333073725
TCGA-BH-A0C3-01A-21R-A12P-07	1450.33547581659	1269.47053515632	2719.80601097292	0.536743819465401
TCGA-B6-A40B-01A-11R-A239-07	592.21759639822	339.635486402742	931.853082800962	0.737272274752907
TCGA-BH-A0HO-01A-11R-A034-07	653.658484629202	912.115045298707	1565.77352992791	0.671307700915968
TCGA-AR-A1AS-01A-11R-A12P-07	255.866400420845	176.744532749994	432.610933170839	0.784758790192069
TCGA-E9-A1RG-01A-11R-A14D-07	432.00492443834	785.139449655543	1217.14437409388	0.70834192505652
TCGA-D8-A27K-01A-11R-A16F-07	768.788529520448	868.194698112384	1636.98322763283	0.663523806716314
TCGA-E9-A1R2-01A-11R-A14D-07	495.736841382643	512.884077716102	1008.62091909875	0.729612589335591

TCGA-A7-A13D-01B-04R-A277-07	195.406856483356	-615.7029058	-420.2960494	0.856011182073553
TCGA-A7-A13D-01A-13R-A277-07	188.969449608382	-318.0351602	-129.0657106	0.833136323123197
TCGA-C8-A26V-01A-11R-A16F-07	259.961935424129	498.48052138479	758.442456808919	0.754228641728574
TCGA-BH-A1ET-01A-11R-A137-07	773.308519113269	453.284613676662	1226.59313278993	0.707362225562533
TCGA-AO-A0JA-01A-11R-A056-07	1339.29134880536	858.247695163687	2197.53904396904	0.59978779969925
TCGA-5L-AAT0-01A-12R-A41B-07	1196.25030010135	1318.7811633109	2515.03146341224	0.561859281537576
TCGA-AC-A6NO-01A-12R-A33J-07	88.4790103106111	-127.0921949	-38.61318459	0.825719812908859
TCGA-C8-A3M7-01A-12R-A21T-07	875.769319115385	599.192571353369	1474.96189046875	0.681127750009076
TCGA-A2-A0ET-01A-31R-A034-07	-531.7900526	481.87078896788	-49.91926367	0.826654824027712
TCGA-A7-A6VW-01A-21R-A33J-07	94.1287440152293	915.907801233977	1010.03654524921	0.729470469040271
TCGA-AR-A0TS-01A-11R-A115-07	784.191174800623	2194.60512006528	2978.7962948659	0.504287251862545
TCGA-A2-A0SW-01A-11R-A084-07	923.845970761137	1762.32897520926	2686.1749459704	0.540902537448574
TCGA-OL-A66J-01A-11R-A29R-07	950.561177766279	540.36534488446	1490.92652265074	0.679410110123026
TCGA-A2-A1G6-01A-11R-A13Q-07	691.536551373197	658.777975279702	1350.3145266529	0.694409254001473
TCGA-E2-A14V-01A-11R-A12D-07	-67.02563991	-233.0132046	-300.0388446	0.846752772529984
TCGA-AO-A03R-01A-21R-A034-07	245.503539538774	1261.47475094578	1506.97829048455	0.677679333632003
TCGA-A8-A06T-01A-11R-A00Z-07	468.047325641157	550.29212838362	1018.33945402478	0.728636273315002
TCGA-AN-A0FK-01A-11R-A034-07	199.194247286398	64.8744129971063	264.068660283505	0.799850989854635
TCGA-A8-A0A2-01A-11R-A034-07	803.083724166713	705.305106056946	1508.38883022366	0.67752706268625
TCGA-AN-A049-01A-21R-A00Z-07	923.464464287822	861.94617812004	1785.41064240786	0.647067244922485
TCGA-EW-A1P8-01A-11R-A144-07	409.239227481099	1424.23709536849	1833.47632284959	0.641671850537403
TCGA-AN-A0AM-01A-11R-A034-07	480.55800804415	979.031631971694	1459.58964001584	0.682778120581953

TCGA-A8-A09G-01A-21R-A00Z-07	314.252282225414	1156.05617423677	1470.30845646219	0.681627710832254
TCGA-A7-A26E-01A-11R-A169-07	1020.95301935533	253.421480710774	1274.3745000661	0.702387189517642
TCGA-A2-A0T5-01A-21R-A084-07	504.96070620355	180.948622503624	685.909328707174	0.761176721695703
TCGA-A2-A25D-01A-12R-A16F-07	717.526689258507	1991.3108135851	2708.83750284361	0.538101595049166
TCGA-D8-A1Y1-01A-21R-A14M-07	1215.81177107976	749.126274878425	1964.93804595818	0.626752858696895
TCGA-3C-AALJ-01A-31R-A41B-07	639.740164180761	1181.96122604073	1821.70139022149	0.642996556447185
TCGA-D8-A141-01A-11R-A115-07	1722.53529333709	2178.03355268044	3900.56884601753	0.383193155379113
TCGA-A2-A4S3-01A-21R-A266-07	167.210574846151	1053.96476653023	1221.17534137638	0.707924138651817
TCGA-B6-A0I9-01A-11R-A034-07	-223.8191736	353.177200895458	129.358027298219	0.811562149535623
TCGA-AR-A2LQ-01A-22R-A18M-07	1798.18102261739	833.106521762147	2631.28754437954	0.547661392890406
TCGA-BH-A0BZ-01A-31R-A12P-07	1416.04628410693	1826.6269565294	3242.67324063633	0.470469097498055
TCGA-B6-A401-01A-11R-A239-07	604.780339158418	1029.66848828563	1634.44882744405	0.66380209
TCGA-E9-A2JS-01A-11R-A180-07	362.607232823759	96.3785974825037	458.985830306262	0.78235333627797
TCGA-AR-A24X-01A-11R-A169-07	705.463361968807	257.539429573789	963.002791542596	0.734175473788557
TCGA-BH-A1FE-06A-11R-A213-07	1256.47462383881	578.656925936365	1835.13154977517	0.641485480088317
TCGA-BH-A18K-01A-11R-A12D-07	843.333823682752	265.026040740307	1108.35986442306	0.719522677530236
TCGA-AN-A04D-01A-21R-A034-07	-948.4095244	-723.9121427	-1672.321667	0.936069403091687
TCGA-BH-A1ES-01A-11R-A137-07	545.344281682826	-533.285812	12.0584696691616	0.821501373772669
TCGA-EW-A1P5-01A-11R-A144-07	326.66570879543	1050.30296968617	1376.9686784816	0.691588573770333
TCGA-AO-A0JG-01A-31R-A084-07	761.77036967678	1303.6403952582	2065.41076493498	0.61519302638176
TCGA-EW-A1P4-01A-21R-A144-07	308.368565522574	1612.0735682399	1920.44213376247	0.631828904224197
TCGA-C8-A26Y-01A-11R-A16F-07	-509.3173585	570.861516564755	61.5441580237014	0.817337799677808

TCGA-A8-A07I-01A-11R-A00Z-07	1150.16813938533	864.895651594657	2015.06379097999	0.621002553140435
TCGA-E9-A1RC-01A-11R-A157-07	65.6107340871226	-441.4591694	-375.8484354	0.852620153625634
TCGA-D8-A13Z-01A-11R-A115-07	791.163442373015	1228.83154340943	2019.99498578244	0.620435036808701
TCGA-BH-A42V-01A-11R-A24H-07	908.597135123464	969.899755712901	1878.49689083637	0.636589295392148
TCGA-BH-A0HL-01A-11R-A10U-07	-876.3325314	-1059.320098	-1935.65263	0.948965848879321
TCGA-A2-A4RY-01A-31R-A266-07	956.625849988263	857.698674153416	1814.32452414168	0.643825491727616
TCGA-EW-A1IY-01A-11R-A13Q-07	1303.91467200103	1604.88910309453	2908.80377509556	0.513132526748848
TCGA-A8-A082-01A-11R-A00Z-07	430.647054205243	407.171848320387	837.81890252563	0.746527078465161
TCGA-B6-A0RU-01A-11R-A084-07	375.944027055241	-95.42215336	280.521873693607	0.798399094298013
TCGA-AR-A0TV-01A-21R-A084-07	-38.93432576	1306.14165387596	1267.2073281156	0.703135650166579
TCGA-A2-A0EN-01A-13R-A084-07	1645.35132453028	2144.92167107518	3790.27299560547	0.398096610242887
TCGA-GM-A5PV-01A-11R-A28M-07	-380.9918923	607.672328141948	226.680435845497	0.803132912336207
TCGA-BH-A1F2-01A-31R-A13Q-07	1200.47234638178	344.771297413166	1545.24364379495	0.673538227950789
TCGA-E9-A1NG-01A-21R-A14M-07	1969.13332526656	1332.41011393309	3301.54343919965	0.462826257774401
TCGA-AC-A2B8-01A-11R-A17B-07	1398.24319300565	1218.3452505137	2616.58844351934	0.549465428842894
TCGA-GM-A2DB-01A-31R-A18M-07	-280.2297384	1093.66296155553	813.433223115354	0.748903952272842
TCGA-AO-A12C-01A-11R-A10J-07	1231.24103440065	-25.0802886	1206.16074579959	0.709479055559448
TCGA-AC-A2BK-01A-11R-A21T-07	-1174.283279	-614.8325308	-1789.115809	0.9419630793982
TCGA-AC-A3QP-01A-11R-A22U-07	1186.35188044309	1355.51765648067	2541.86953692376	0.558596031501684
TCGA-E2-A15A-06A-11R-A12D-07	478.393019236462	1739.10308868664	2217.4961079231	0.597441190948361
TCGA-A2-A0YI-01A-31R-A10J-07	1252.43703975799	1778.97316793777	3031.41020769576	0.497603093695375
TCGA-A8-A07R-01A-21R-A034-07	-475.63045	-91.57798984	-567.2084399	0.866959483681041

TCGA-BH-A0DT-01A-21R-A12D-07	887.850526919642	866.559559270506	1754.41008619015	0.650530001348382
TCGA-A8-A08A-01A-11R-A32Y-07	760.846169392244	824.366812535541	1585.21298192778	0.669190027477621
TCGA-AC-A23H-01A-11R-A157-07	349.216155237531	-102.9422789	246.273876347333	0.801416018971683
TCGA-B6-A0WV-01A-11R-A109-07	-545.5977383	-900.2331127	-1445.830851	0.923857747598174
TCGA-C8-A12V-01A-11R-A115-07	951.600515656599	2931.76744	3883.3679552446	0.385524093286178
TCGA-A2-A1G1-01A-21R-A13Q-07	1070.91823811682	1469.07518579894	2539.99342391577	0.558824431107658
TCGA-E9-A6HE-01A-11R-A31O-07	838.455310929606	960.777806856721	1799.23311778633	0.645518953626934
TCGA-JL-A3YX-01A-11R-A22U-07	96.617263075883	-219.5060434	-122.8887804	0.832634513311825
TCGA-PE-A5DC-01A-12R-A27Q-07	73.4172977360752	186.956460520839	260.373758256915	0.800176401179093
TCGA-AC-A6IW-01A-12R-A33J-07	9.74351642981946	2325.17782040773	2334.92133683755	0.583530828717136
TCGA-AC-A3TN-01A-11R-A22K-07	220.358949887068	202.962934194964	423.321884082032	0.785603174760128
TCGA-EW-A1P0-01A-11R-A144-07	334.565364039916	414.786582455366	749.351946495282	0.755104140705499
TCGA-BH-A0GY-01A-11R-A056-07	585.754829720766	771.429567456415	1357.18439717718	0.693683263839321
TCGA-D8-A1X8-01A-11R-A14M-07	1019.58093605937	952.913049774269	1972.49398583364	0.625888223618145
TCGA-A7-A13D-01A-13R-A12P-07	121.86887569414	-152.5762514	-30.70737572	0.825064652349167
TCGA-BH-A18H-01A-11R-A12D-07	690.958508104614	1864.34250100004	2555.30100910465	0.556959636283956
TCGA-C8-A1HN-01A-11R-A137-07	162.455432595254	274.352446410061	436.807879005315	0.784376804674249
TCGA-E2-A106-01A-11R-A10J-07	-384.5745657	-505.033085	-889.6076506	0.889565540218656
TCGA-A2-A0YE-01A-11R-A109-07	135.809917019343	-159.3128011	-23.50288411	0.824466642826506
TCGA-A7-A4SD-01A-11R-A266-07	640.672909262703	1447.15669029551	2087.82959955822	0.612595290881683
TCGA-E9-A227-01A-11R-A157-07	957.718236551174	1417.89215009267	2375.61038664384	0.578670089443444
TCGA-EW-A1J6-01A-11R-A13Q-07	23.7149852881875	1240.14230415911	1263.8572894473	0.703485224531584

TCGA-AQ-A04J-01A-02R-A034-07	1115.18524727918	1899.79296357025	3014.97821084943	0.499693847661113
TCGA-B6-A0RG-01A-11R-A056-07	223.678978766478	-285.5450746	-61.86609586	0.827640351238435
TCGA-A8-A09X-01A-11R-A00Z-07	1244.64077213478	1757.04233724569	3001.68310938047	0.501383346146345
TCGA-A2-A0YH-01A-11R-A109-07	1243.57301026879	1714.00259002502	2957.57560029381	0.506974673903789
TCGA-AN-A041-01A-11R-A034-07	958.673693279112	262.953094566145	1221.62678784526	0.707877333404871
TCGA-E2-A1IE-01A-11R-A13Q-07	-205.9774456	-677.0323881	-883.0098337	0.889122712742079
TCGA-A8-A079-01A-21R-A00Z-07	-94.37524268	324.023759262573	229.648516582598	0.802873258124092
TCGA-AO-A12D-01A-11R-A115-07	1277.80246252806	1728.40365717914	3006.2061197072	0.500808790619109
TCGA-BH-A28Q-01A-11R-A16F-07	629.501361974657	252.635836638205	882.137198612862	0.742182903924178
TCGA-B6-A0I2-01A-11R-A034-07	-186.0049836	2032.58475258656	1846.57976895553	0.640195430519211
TCGA-E2-A14Q-01A-11R-A12D-07	349.133488698841	261.181894191369	610.3153828 9021	0.768326167240975
TCGA-GI-A2C8-01A-11R-A16F-07	1609.02551791557	1228.25171063485	2837.27722855042	0.522115698700434
TCGA-C8-A27A-01A-11R-A169-07	-354.306144	-347.6033858	-701.9095298	0.876643596472832
TCGA-AC-A2FG-01A-11R-A17B-07	513.356129193048	242.670917130223	756.027046323271	0.75446139884224
TCGA-A8-A07W-01A-11R-A00Z-07	551.523003118737	638.419006756729	1189.94200987547	0.711154801511014
TCGA-A8-A09B-01A-11R-A00Z-07	562.94004596157	142.643531964601	705.583577926172	0.75930058492428
TCGA-B6-A0IG-01A-11R-A034-07	49.5905122098405	69.1196617832719	118.710173993112	0.812474354897326
TCGA-PL-A8LV-01A-21R-A41B-07	105.937628910075	1060.3313956594	1166.26902456948	0.713593494678953
TCGA-A8-A06N-01A-11R-A00Z-07	-1370.134208	-493.8998766	-1864.034084	0.945597965537771
TCGA-AR-A5QP-01A-11R-A28M-07	724.450783352025	439.826464037899	1164.27724738992	0.71379828675608
TCGA-BH-A0W3-01A-11R-A109-07	-263.2814699	132.440040118628	-130.8414298	0.833280454669454
TCGA-AN-A0XT-01A-11R-A109-07	234.78534020349	-17.60231595	217.18302425 1002	0.803962742121372

TCGA-BH-A1FD-01A-11R-A13Q-07	710.282851512428	364.375453565915	1074.65830507834	0.722949395639141
TCGA-AR-A1AN-01A-11R-A12P-07	1264.76938878262	682.436548966888	1947.20593774951	0.628778935014625
TCGA-BH-A0HX-01A-21R-A056-07	781.480348725703	439.550539297669	1221.03088802337	0.707939114687629
TCGA-AN-A0AL-01A-11R-A00Z-07	1004.90169898157	1309.39395071264	2314.29564969421	0.585986846620925
TCGA-EW-A6SD-01A-12R-A33J-07	566.500450592249	1269.50892917869	1836.00937977094	0.641386625360052
TCGA-A2-A0D3-01A-11R-A115-07	-50.92125398	522.912804412546	471.99155042847	0.78116286423377
TCGA-C8-A12L-01A-11R-A115-07	-143.8709867	344.891376939223	201.020390206364	0.805371348066834
TCGA-AO-A12F-01A-11R-A115-07	631.088487061121	-50.16888454	580.919602519596	0.771080832603477
TCGA-BH-A0DZ-01A-11R-A00Z-07	1396.31804915857	1134.72259961329	2531.04064877186	0.559913765654911
TCGA-AC-A3W5-01A-11R-A22K-07	1572.02420181196	1822.56394050818	3394.58814232014	0.450676450543142
TCGA-E2-A15H-01A-11R-A12D-07	283.724387468389	-68.75308793	214.971299539478	0.804155765792906
TCGA-E2-A108-01A-13R-A10J-07	2043.21365687754	2684.07847568456	4727.2921325621	0.268559688069293
TCGA-AQ-A1H2-01A-11R-A13Q-07	-6.78165597	352.383825923314	345.602169953813	0.792610596541835
TCGA-D8-A1JK-01A-11R-A13Q-07	1058.35970917533	1715.6287845797	2773.98849375503	0.530016304382693
TCGA-GM-A2DN-01A-11R-A180-07	786.266767004865	1955.14077544368	2741.40754244854	0.534065732401193
TCGA-B6-A0RL-01A-11R-A084-07	-1928.255743	-806.4409107	-2734.696654	0.979352003164243
TCGA-BH-A0BW-01A-11R-A115-07	442.027860409795	1871.34697377209	2313.37483418188	0.586096368275653
TCGA-A8-A08O-01A-21R-A056-07	626.518971046639	-346.7307784	279.788192666929	0.798463936430793
TCGA-EW-A1J5-01A-11R-A13Q-07	658.818636312333	1617.63228940138	2276.45092571371	0.590479247384964
TCGA-A8-A06P-01A-11R-A00Z-07	1402.37301451218	572.136404696751	1974.50941920893	0.625657465090057
TCGA-GM-A2DO-01A-11R-A18M-07	325.665527465528	3058.53501905097	3384.20054651649	0.452037076682251
TCGA-HN-A2OB-01A-21R-A27Q-07	1369.53501019399	350.772536265455	1720.30754645944	0.654323686049599

TCGA-AN-A04C-01A-21R-A034-07	-121.5296891	498.749632878906	377.219943738304	0.789772247206389
TCGA-AR-A24W-01A-11R-A169-07	849.187542832722	-90.39366418	758.793878655026	0.754194769630937
TCGA-E2-A1IF-01A-11R-A144-07	971.954699684032	607.899199182222	1579.85389886625	0.669774374061202
TCGA-GM-A3XG-01A-31R-A24H-07	-1.207904453	-146.156534	-147.3644384	0.834618878350829
TCGA-A2-A0ER-01A-21R-A034-07	-0.257751462	223.523323469891	223.265572007562	0.803431463513503
TCGA-A2-A0D4-01A-11R-A00Z-07	-211.5007809	-439.0246654	-650.5254463	0.872989651705185
TCGA-AC-A6IX-06A-11R-A32P-07	264.369801817639	1933.22525453236	2197.595056	0.599781220796586
TCGA-GM-A2DM-01A-11R-A180-07	-179.8829534	725.529384198856	545.646430790894	0.774367311880222
TCGA-AQ-A04L-01B-21R-A10J-07	602.478032858332	191.26076229327	793.738795151602	0.750816566602235
TCGA-AC-A2FK-01A-12R-A180-07	545.840926454225	676.573551567408	1222.41447802163	0.707795659505262
TCGA-A2-A0CY-01A-12R-A034-07	-874.6992636	-417.4981179	-1292.197381	0.914992249651978
TCGA-A8-A08I-01A-11R-A00Z-07	139.428298860948	2246.1668469321	2385.59514579304	0.577474142895993
TCGA-OL-A5RZ-01A-11R-A28M-07	-332.1514505	-255.8270714	-587.9785219	0.868474912330113
TCGA-A8-A08C-01A-11R-A00Z-07	114.526840381956	-16.04874728	98.4780931064886	0.814202173942211
TCGA-A8-A09C-01A-11R-A00Z-07	290.334892795541	-286.6803115	3.65458128858972	0.822204151511643
TCGA-AN-A0AS-01A-11R-A00Z-07	1047.92609349566	749.279390581912	1797.20548407757	0.645746240606757
TCGA-BH-A0DE-01A-11R-A115-07	1252.83507930558	1082.49088248591	2335.32596179149	0.583482594153111
TCGA-D8-A1JG-01B-11R-A13Q-07	1001.16094025184	1385.60607963609	2386.76701988793	0.577333697703013
TCGA-A8-A09Z-01A-11R-A00Z-07	57.3493158032415	1254.36941364353	1311.71872944678	0.698474809195187
TCGA-BH-A28O-01A-11R-A22K-07	1201.04353730669	593.617611493569	1794.66114880026	0.646031366161388
TCGA-C8-A132-01A-31R-A115-07	1211.7646075298	1525.82546323653	2737.59007076632	0.534539401416071
TCGA-EW-A1PB-01A-11R-A144-07	241.669989266369	2450.58950886196	2692.25949812833	0.540151113494035

TCGA-A8-A07P-01A-11R-A00Z-07	718.706930298191	538.497501471553	1257.20443176974	0.704178941476329
TCGA-MS-A51U-01A-31R-A266-07	951.895377215635	727.384861890282	1679.28023910592	0.658865968851953
TCGA-A2-A0CO-01A-13R-A22K-07	1259.74949605629	1937.38294897873	3197.13244503502	0.476357372622561
TCGA-BH-A6R8-01A-21R-A33J-07	110.199544714789	990.920591592403	1101.12013630719	0.720260288873761
TCGA-EW-A1OZ-01A-11R-A144-07	793.005920946984	795.924022305868	1588.92994325285	0.668784492212729
TCGA-A2-A0EW-01A-21R-A115-07	1449.23146289494	1030.03055959218	2479.26202248712	0.566194935100278
TCGA-B6-A3ZX-01A-11R-A239-07	350.843673617671	2504.65588412285	2855.49955774053	0.519832541832958
TCGA-S3-AA14-01A-11R-A41B-07	875.527117697646	-15.00449457	860.522623124357	0.744305546242104
TCGA-AO-A03N-01B-11R-A10J-07	524.628049574507	108.604332302864	633.232381877372	0.766168699466568
TCGA-AR-A1AP-01A-11R-A12P-07	753.522104191258	915.168084356699	1668.69018854796	0.660034558459917
TCGA-A2-A0T0-01A-22R-A084-07	534.497720719673	609.360380653145	1143.85810137282	0.715894236039726
TCGA-AN-A0XS-01A-22R-A109-07	1261.19996710729	1222.16373237209	2483.36369947938	0.56569855635138
TCGA-D8-A73U-01A-11R-A33J-07	1686.86336252615	2962.60744900473	4649.47081153088	0.279545537781128
TCGA-BH-A0DV-01A-21R-A12P-07	1891.53133314074	1012.34616430385	2903.87749744459	0.513753053577624
TCGA-AR-A24Q-01A-12R-A169-07	953.470682393135	971.316900352156	1924.78758274529	0.631334364240604
TCGA-BH-A0BS-01A-11R-A12P-07	-220.5790645	-37.11722463	-257.6962891	0.843429930993523
TCGA-AN-A0FV-01A-11R-A00Z-07	628.385720180544	-57.31795712	571.067763063087	0.772000834527132
TCGA-A2-A3KC-01A-11R-A213-07	1186.83780918985	837.086590274521	2023.92439946437	0.619982579705252
TCGA-B6-A0IJ-01A-11R-A034-07	-13.50749801	1984.54505291751	1971.03755491174	0.626054944653993
TCGA-AR-A24N-01A-11R-A169-07	678.883289712303	228.869845791367	907.75313550367	0.739657638984656
TCGA-D8-A1XU-01A-11R-A14M-07	1067.68436526319	582.035451289002	1649.71981655219	0.66212390934266
TCGA-A8-A08T-01A-21R-A00Z-07	506.942340632022	155.416942835118	662.35928346714	0.763414105563734

TCGA-BH-A18T-01A-11R-A12D-07	-190.1926888	380.844848640132	190.652159876013	0.806272573872872
TCGA-LL-A440-01A-11R-A24H-07	1766.20171223582	1707.61106063973	3473.81277287555	0.440264919615972
TCGA-BH-A0AU-01A-11R-A12P-07	825.922978967226	598.598162831792	1424.52114179902	0.686530062671879
TCGA-AR-A2LH-01A-31R-A18M-07	917.433477252353	2080.00249951114	2997.43597676349	0.501922655884713
TCGA-A7-A26E-01A-11R-A277-07	1261.79902185883	485.907992388421	1747.70701424725	0.651276964470398
TCGA-OL-A6VQ-01A-12R-A41B-07	1067.07718648323	721.540799310598	1788.61798579383	0.646708219184667
TCGA-D8-A1X9-01A-12R-A157-07	1025.03787530861	811.016556692632	1836.05443200124	0.641381551623429
TCGA-A8-A09D-01A-11R-A00Z-07	1614.45910324366	1077.16832893452	2691.62743217818	0.540229191809822
TCGA-D8-A1X6-01A-11R-A14M-07	393.178038659234	935.183907270747	1328.36194592998	0.696724408846462
TCGA-A8-A084-01A-21R-A00Z-07	306.648035931999	-62.9087477	243.73928822705	0.801638488212929
TCGA-A7-A0DA-01A-31R-A115-07	545.816467867701	32.4329325757126	578.249400443413	0.771330345526218
TCGA-BH-A1EV-01A-11R-A137-07	634.202699390293	-119.5633367	514.639362669563	0.777239166517884
TCGA-D8-A1JM-01A-11R-A13Q-07	-1061.105059	186.994151148287	-874.1109081	0.888524120238995
TCGA-A8-A09W-01A-11R-A00Z-07	903.78685931737	643.376122493633	1547.162982	0.673329954973321
TCGA-E2-A56Z-01A-12R-A29R-07	-109.9685002	-16.78332982	-126.75183	0.832948425172033
TCGA-A1-A0SF-01A-11R-A144-07	673.504090565943	950.593338524754	1624.0974290907	0.664937743764982
TCGA-E9-A22A-01A-11R-A157-07	1763.77367791925	1616.19472801301	3379.96840593225	0.452591125288698
TCGA-A8-A0A6-01A-12R-A056-07	862.220073597765	1826.00672759346	2688.22680119122	0.540649187667507
TCGA-AN-A0XN-01A-21R-A109-07	1842.84935173086	1327.80682929265	3170.65618102351	0.479770897978913
TCGA-A2-A0CR-01A-11R-A22K-07	1178.07744977087	2750.50651992912	3928.58396969999	0.379391529579326
TCGA-B6-A0X5-01A-21R-A109-07	-283.9468839	-241.2503011	-525.197185	0.863869634613624
TCGA-E9-A22G-01A-11R-A157-07	-795.1060918	182.277493267243	-612.8285985	0.870277417663927

TCGA-AR-A24K-01A-11R-A169-07	-30.08519455	-504.2586115	-534.3438061	0.864545153162328
TCGA-BH-A0BG-01A-11R-A115-07	278.936317161233	1615.18734455784	1894.12366171907	0.634818614615387
TCGA-E2-A14R-01A-11R-A115-07	-236.156133	1426.80578294672	1190.64964990186	0.711081771028834
TCGA-E9-A247-01A-11R-A169-07	-142.9509318	-368.3176731	-511.2686049	0.862837956327451
TCGA-AR-A0TQ-01A-11R-A084-07	455.885738330886	1277.76454391045	1733.65028224134	0.652841341241559
TCGA-D8-A27L-01A-11R-A16F-07	1275.87981697042	1026.5337679895	2302.41358495992	0.587399274622078
TCGA-A2-A0CL-01A-11R-A115-07	1488.85175112016	2796.3731747962	4285.22492591635	0.330457073981498
TCGA-AR-A1AY-01A-21R-A12P-07	57.9676229776888	87.8016666987032	145.769289676392	0.810152308994944
TCGA-GM-A3XL-01A-11R-A22U-07	-451.6559806	1513.97481516014	1062.31883455397	0.724199631867564
TCGA-LL-A6FP-01A-11R-A31O-07	-889.054178	-424.6962203	-1313.750398	0.91626414412868
TCGA-E9-A1RD-01A-11R-A157-07	620.617493894131	259.283048826041	879.900542720172	0.742402899868699
TCGA-AR-A1AO-01A-11R-A12P-07	1751.53003069543	2670.22092474452	4421.75095543995	0.31147739716609
TCGA-WT-AB44-01A-11R-A41B-07	708.883563605828	548.842640156348	1257.72620376218	0.704124558730161
TCGA-E2-A573-01A-11R-A29R-07	107.072998528074	1902.27666535796	2009.34966388603	0.621659767804137
TCGA-A8-A09M-01A-11R-A00Z-07	374.013533446378	1261.02000305547	1635.03353650185	0.663737895595592
TCGA-4H-AAAK-01A-12R-A41B-07	1238.90752519716	582.901580866554	1821.80910606372	0.642984446876963
TCGA-AC-A3TM-01A-11R-A22K-07	341.697700842087	934.047938266844	1275.74563910893	0.70224391421389
TCGA-BH-A18N-01A-11R-A12D-07	179.184839115217	-2.984068937	176.20077017851	0.807525599418126
TCGA-BH-A18V-01A-11R-A12D-07	856.187665640945	1345.94824060552	2202.13590624647	0.599247742989282
TCGA-BH-A1FN-01A-11R-A13Q-07	519.934810813827	187.231800785786	707.166611599613	0.759149351321133
TCGA-GM-A3NW-01A-21R-A22K-07	116.543889718325	350.291571242282	466.835460960607	0.781635165145105
TCGA-A2-A04V-01A-21R-A034-07	909.84248512942	495.082633684317	1404.92511881374	0.688618704178861

TCGA-C8-A12W-01A-11R-A115-07	1127.11473546774	798.159216602685	1925.27395207042	0.631278996301995
TCGA-BH-A0E6-01A-11R-A034-07	140.387476748888	910.09633002923	1050.48380677812	0.72539652573793
TCGA-A8-A08J-01A-11R-A00Z-07	860.262853489512	548.832964952645	1409.09581844216	0.688174646984888
TCGA-LL-A9Q3-01A-11R-A41B-07	452.219654519184	963.171244411662	1415.39089893085	0.687503917130225
TCGA-A8-A06O-01A-11R-A00Z-07	610.194736230298	532.749306143108	1142.94404237341	0.715987910536772
TCGA-A8-A08G-01A-11R-A00Z-07	559.047836570359	566.441973647044	1125.4898102174	0.7177741783219
TCGA-A7-A13G-01B-04R-A22O-07	721.562829512999	148.213594062151	869.77642357515	0.743397700045603
TCGA-D8-A1XA-01A-11R-A14D-07	389.649479933652	321.549056335904	711.198536269556	0.758763980128463
TCGA-A7-A13G-01A-11R-A13Q-07	242.59999727763	-331.9061937	-89.30619638	0.829894319232052
TCGA-BH-A1EN-01A-11R-A13Q-07	482.73509886973	-27.94264924	454.792449625397	0.782736567584396
TCGA-C8-A12Y-01A-11R-A12D-07	521.813678558522	421.203715101431	943.017393659954	0.736164124799207
TCGA-AO-A12E-01A-11R-A10J-07	738.759137249519	736.798317236992	1475.55745448651	0.68106374019458
TCGA-A8-A07J-01A-11R-A00Z-07	1812.52524058974	1567.67324322879	3380.19848381852	0.452561009242973
TCGA-A7-A13F-01A-11R-A12P-07	260.947271844464	280.436197654966	541.38346949943	0.774763097073261
TCGA-A2-A0YD-01A-11R-A109-07	1592.92715485986	1270.48542537947	2863.41258023933	0.518839924793715
TCGA-5T-A9QA-01A-11R-A41B-07	-1318.045365	-289.7082691	-1607.753634	0.932692960639931
TCGA-BH-A42U-01A-12R-A24H-07	844.591411381214	1433.24330782748	2277.83471920869	0.590315302734741
TCGA-E2-A1LI-01A-12R-A157-07	-237.9429662	1010.28468261734	772.341716443275	0.75288742043986
TCGA-A8-A09N-01A-11R-A00Z-07	452.717128881497	287.061122475581	739.778251357078	0.756024721139139
TCGA-C8-A135-01A-11R-A115-07	846.118096865214	884.592490873553	1730.71058773877	0.653168149290363
TCGA-D8-A1XY-01A-11R-A14M-07	1143.27970746946	250.027580802533	1393.307288	0.68985430107219
TCGA-AN-A0XW-01A-11R-A109-07	1667.92301595438	1822.78880404418	3490.71181999856	0.438036330485866

TCGA-BH-A201-01A-11R-A14M-07	1018.24979658895	80.6087956331918	1098.85859222214	0.720490536985738
TCGA-A8-A085-01A-11R-A00Z-07	330.627369030939	-628.1361587	-297.5087897	0.846555143517007
TCGA-LD-A74U-01A-13R-A33J-07	1263.7205011085	1116.58740725864	2380.30790836714	0.578107588101946
TCGA-A8-A0A1-01A-11R-A00Z-07	1254.36000959534	1770.51611929592	3024.87612889127	0.498434816070494
TCGA-GM-A2DK-01A-21R-A180-07	916.296038249628	1004.08293836443	1920.37897661405	0.631836090018225
TCGA-LL-A442-01A-11R-A24H-07	-190.8186518	646.585314034053	455.766662242908	0.782647561141852
TCGA-A2-A259-01A-11R-A16F-07	939.239474230772	1000.60915890166	1939.84863313243	0.629618333329459
TCGA-AC-A2FF-01A-11R-A17B-07	1719.92829538817	1480.56433171502	3200.49262710319	0.475923636295936
TCGA-C8-A1HI-01A-11R-A137-07	433.461022725701	144.716368451419	578.17739117712	0.77133707268053
TCGA-LL-A73Z-01A-11R-A32P-07	1063.57772034356	1401.98296639941	2465.56068674297	0.567851561114737
TCGA-AO-A0JM-01A-21R-A056-07	907.233234122352	562.031176860223	1469.26441098258	0.681739838479008
TCGA-OL-A5RX-01A-11R-A28M-07	435.514909760741	627.657922544162	1063.1728323049	0.724113181221458
TCGA-BH-A0DH-01A-11R-A084-07	353.932596649472	533.296815142641	887.229411792113	0.741681738948522
TCGA-AO-A0JB-01A-11R-A32Y-07	437.990721221072	689.939684079298	1127.93040530037	0.717524690670201
TCGA-A2-A0T7-01A-21R-A084-07	910.702366961156	531.038021639602	1441.74038860076	0.68469006047722
TCGA-E9-A1R3-01A-31R-A14M-07	1074.38654476512	1038.25149983705	2112.63804460217	0.609712932959794
TCGA-AN-A0XL-01A-11R-A10J-07	1229.73788250524	193.429185810844	1423.16706831608	0.686674569445546
TCGA-E2-A10A-01A-21R-A115-07	358.339590889764	335.434363776523	693.773954666287	0.760427511588594
TCGA-EW-A1PH-01A-11R-A14M-07	-139.9435774	293.001084901068	153.057507510231	0.80952469278818
TCGA-S3-AA17-01A-11R-A41B-07	704.810832960595	2373.57398932915	3078.38482228975	0.491610270382176
TCGA-B6-A0X4-01A-11R-A109-07	-1120.621629	-882.8725904	-2003.494219	0.952059399020188
TCGA-A2-A3XX-01A-21R-A239-07	224.821197002495	837.315798026119	1062.13699502861	0.724218038109809

TCGA-OL-A5DA-01A-11R-A27Q-07	178.828678478235	390.283579168735	569.11225764697	0.772183255023053
TCGA-EW-A1PA-01A-11R-A144-07	458.945447126342	403.477645739157	862.423092865499	0.744119212231084
TCGA-EW-A1OY-01A-11R-A144-07	47.167254965032	286.046218028899	333.213472993931	0.793718086394711
TCGA-D8-A1JH-01A-11R-A13Q-07	1362.16793331801	1014.73585879492	2376.90379211293	0.578515238978415
TCGA-AR-A1AT-01A-11R-A12P-07	697.91451834266	1773.3698002321	2471.28431857476	0.56715979678835
TCGA-AC-A7VB-01A-11R-A352-07	-747.4022437	-813.8523319	-1561.254576	0.930209483236457
TCGA-AN-A0FJ-01A-11R-A00Z-07	330.322687258027	81.2605737278682	411.583260985896	0.786668138919216
TCGA-A2-A1FW-01A-11R-A13Q-07	428.604117015096	-50.21713701	378.386980002927	0.789667155634729
TCGA-BH-A8G0-01A-11R-A352-07	1448.82800961089	1807.08461326768	3255.91262287857	0.46875333280623
TCGA-A7-A6VV-01A-22R-A33J-07	1060.31744407896	1051.2414177868	2111.55886186576	0.609838485805199
TCGA-EW-A6SB-01A-12R-A32P-07	-312.2251862	2282.55024564212	1970.32505948895	0.626136495240049
TCGA-E2-A15I-01A-21R-A137-07	529.641946967538	938.23598691625	1467.87793388379	0.681888717599943
TCGA-EW-A1IW-01A-11R-A13Q-07	806.047702054053	636.735833126351	1442.7835351804	0.684578452009494
TCGA-D8-A1J8-01A-11R-A13Q-07	895.349654730796	1539.81786921111	2435.16752394191	0.571518184440432
TCGA-D8-A1XO-01A-11R-A14M-07	1250.95262722538	824.748777798866	2075.70140502425	0.614001445506419
TCGA-E9-A22E-01A-11R-A157-07	1971.50366577108	1246.88632541391	3218.38999118499	0.473611474954411
TCGA-BH-A0B0-01A-21R-A115-07	1290.31341870529	1364.09938244695	2654.41280115224	0.544818048424833
TCGA-GM-A2DI-01A-31R-A18M-07	1494.6856959498	2646.02033562993	4140.70603157974	0.350403152230282
TCGA-A7-A26J-01A-11R-A277-07	788.290686418685	387.876897426202	1176.16758384489	0.712574832508853
TCGA-B6-A0I8-01A-11R-A034-07	336.695982291795	-388.1085832	-51.41260095	0.82677815263582
TCGA-3C-AAAU-01A-11R-A41B-07	-483.3692911	-430.5977076	-913.9669987	0.891193242988271
TCGA-A7-A26J-01B-02R-A277-07	1159.43065509869	947.386416875155	2106.81707197385	0.610389967443054

TCGA-AR-A2LJ-01A-12R-A19W-07	1195.23333670225	1879.50958055406	3074.7429172563	0.492075728769257
TCGA-BH-A0H3-01A-11R-A12P-07	1231.50633797005	791.968313751226	2023.47465172127	0.620034376910322
TCGA-C8-A26X-01A-31R-A16F-07	1380.25106582141	2111.93653124038	3492.18759706179	0.437841581834427
TCGA-BH-A18G-01A-11R-A12D-07	303.548746223159	542.774643970531	846.323390193691	0.745695894284275
TCGA-E2-A14N-01A-31R-A137-07	-514.8211076	902.038779069231	387.217671503095	0.788871201324011
TCGA-A8-A075-01A-11R-A084-07	712.473219013768	1052.33752992469	1764.81074893846	0.649369747252167
TCGA-E2-A1L7-01A-11R-A144-07	495.704045354758	1478.83033293205	1974.53437828681	0.625654607038751
TCGA-C8-A12T-01A-11R-A115-07	1024.02323065898	509.112249604786	1533.13548026376	0.674850887023924
TCGA-A2-A0YJ-01A-11R-A109-07	-482.8360963	-262.2733056	-745.1094019	0.87967697685804
TCGA-A2-A25C-01A-11R-A169-07	807.648216246626	497.320365752036	1304.96858199866	0.699183548029786
TCGA-BH-A0C0-01A-21R-A056-07	530.058915202037	2301.8022360506	2831.86115125264	0.522793583759286
TCGA-A7-A6VX-01A-12R-A33J-07	698.887402671731	233.658914072332	932.546316744063	0.737203523191562
TCGA-AN-A0FZ-01A-11R-A034-07	297.041325253396	-293.1466335	3.89469173314842	0.822184089560537
TCGA-A8-A07E-01A-11R-A034-07	1489.41877683688	938.072451572071	2427.49122840895	0.572442454294266
TCGA-AQ-A04H-01B-11R-A10J-07	-4.294300579	-117.2391976	-121.5334982	0.832524319482773
TCGA-D8-A1XW-01A-11R-A14M-07	1268.79220384471	1512.54344370179	2781.33564754649	0.529101461406227
TCGA-AN-A03X-01A-21R-A00Z-07	1523.27863748068	703.040201954938	2226.31883943562	0.596402153379645
TCGA-A7-A13E-01A-11R-A277-07	127.562814593533	-460.3191151	-332.7563005	0.849297886323197
TCGA-A7-A13E-01B-06R-A277-07	1329.54296221478	1480.353726	2809.89668843779	0.525539299998752
TCGA-A8-A08L-01A-11R-A00Z-07	300.159022251564	1155.43683976637	1455.59586201793	0.683206325496197
TCGA-AC-A6IV-01A-12R-A33J-07	1806.30867488201	1892.36361188357	3698.67228676558	0.410394781242062
TCGA-E2-A10F-01A-11R-A10J-07	1437.81590934869	1365.97353511226	2803.78944446095	0.526301780083002

TCGA-W8-A86G-01A-21R-A36F-07	338.875857629571	-383.4599774	-44.5841198	0.826213891941665
TCGA-C8-A130-01A-31R-A115-07	663.597590434926	334.068284743097	997.665875178023	0.730711343703743
TCGA-A8-A07G-01A-11R-A034-07	1309.93216014686	665.309306192079	1975.24146633894	0.625573635256899
TCGA-A7-A3J1-01A-11R-A213-07	1041.16954988019	1752.23495010871	2793.4044999889	0.527597353098165
TCGA-D8-A1XM-01A-21R-A14M-07	1319.86350349182	1310.28892161537	2630.15242510719	0.547800798211608
TCGA-BH-A0BL-01A-11R-A115-07	1596.531431	1775.76795387798	3372.29938529999	0.453594666182128
TCGA-BH-A0AY-01A-21R-A00Z-07	1482.63302953461	1193.69092115136	2676.32395068598	0.542118190792033
TCGA-AC-A3QQ-01B-06R-A22O-07	1123.32029550452	738.441049542024	1861.76134504655	0.638481897586883
TCGA-AO-A0J7-01A-11R-A034-07	-220.788091	-418.3757498	-639.1638408	0.872175010826109
TCGA-E9-A1N6-01A-11R-A144-07	1069.26539902115	494.933417849003	1564.19881687015	0.671479006164437
TCGA-AN-A0G0-01A-11R-A034-07	69.6041197245468	169.92161050244	239.525730226986	0.802008080653167
TCGA-E9-A3X8-01A-31R-A22U-07	1042.28034830442	1529.19375476261	2571.47410306703	0.554986348951334
TCGA-BH-A0H0-01A-11R-A056-07	-309.753331	-133.796018	-443.549349	0.857770730022999
TCGA-E2-A10C-01A-21R-A10J-07	270.865248573588	1308.24396020972	1579.10920878331	0.669855541188885
TCGA-A8-A094-01A-11R-A00Z-07	864.588361412584	1591.25578631344	2455.84414772602	0.56902499444888
TCGA-AC-A5XU-01A-11R-A28M-07	662.376396786784	79.0138336777458	741.39023046453	0.7558698221052
TCGA-AR-A0U2-01A-11R-A109-07	-164.4801341	477.753331551399	313.273197451157	0.795495137025308
TCGA-BH-A1EW-01A-11R-A137-07	605.880113846259	1022.81224387022	1628.69235771648	0.664433822602074
TCGA-EW-A6SC-01A-12R-A32P-07	1138.47491514191	836.410330881723	1974.88524602363	0.625614428463957

Supplementary Table S3: Statistical analysis results

ScoreType	Group	Mean	SD	Median	Pvalue	Significance
StromalScore	Low	665.462849840393	678.13681228214	759.257682317129	0.000569681922127861	***
StromalScore	High	531.147991109411	646.364318966103	602.961799519901	0.000569681922127861	***
ImmuneScore	Low	900.456611039369	878.047046169796	791.409007102369	7.23100093875184e-05	***
ImmuneScore	High	677.762273279726	762.655367218304	594.148726993486	7.23100093875184e-05	***
ESTIMATEScore	Low	1565.91946087976	1367.90012960275	1567.48113219259	1.35653732992187e-05	***
ESTIMATEScore	High	1208.91026438914	1252.34552187139	1208.67060371685	1.35653732992187e-05	***

Supplementary Table S4: Code

**# Load required packages**
library(limma)   # For data processing and analysis
library(reshape2)   # For data reshaping (wide to long format)
library(ggpubr)   # For enhanced ggplot2-based visualization
# Define file paths and set working directory
expFile <- "geneExp.txt"   # Path to gene expression data file
scoreFile <- "TMEscores.txt"   # Path to tumor microenvironment (TME) score file
# Load and process gene expression data
exp <- read.table(expFile, header = TRUE, sep = "\t", check.names = FALSE, row.names = 1)   # Read expression data
gene <- colnames(exp)[1]   # Extract gene name from the first column header
exp <- exp[exp$Type == "Tumor", 1, drop = FALSE]   # Filter to retain only tumor samples, keeping first data column
# Classify samples into High/Low expression groups using median as cutoff
exp$Type <- ifelse(exp[, gene] > median(exp[, gene]), "High", "Low")
# Load and process TME score data
score <- read.table(scoreFile, header = TRUE, sep = "\t", check.names = FALSE, row.names = 1)   # Read TME scores
score <- score[, 1:3]   # Retain only the first 3 columns of TME score data
# Identify and retain common samples between datasets
sameSample <- intersect(row.names(exp), row.names(score))   # Find overlapping sample IDs
exp <- exp[sameSample, "Type", drop = FALSE]   # Keep only common samples with their

```
expression group
score <- score[sameSample, , drop = FALSE]        # Keep only common samples with their TME scores
rt <- cbind(score, exp)    # Combine TME scores and expression group classifications
rt$Type <- factor(rt$Type, levels = c("Low", "High"))    # Set factor levels for consistent plotting order

# Reshape data for visualization with ggplot2
data <- melt(rt, id.vars = c("Type"))    # Convert from wide to long format (required for ggviolin)
colnames(data) <- c("Type", "scoreType", "Score")    # Rename columns for clarity in plotting

# Perform statistical analysis and compile results
stat_results <- data.frame()    # Initialize empty data frame to store results
for (score_type in unique(data$scoreType)) {
  subset_data <- data[data$scoreType == score_type, ]    # Extract data for current score type
  test_result <- wilcox.test(Score ~ Type, data = subset_data)    # Non-parametric test between groups

  # Calculate descriptive statistics (mean, standard deviation, median)
  desc_stats <- aggregate(Score ~ Type, data = subset_data,
                          FUN = function(x) c(mean = mean(x), sd = sd(x), median = median(x)))

  # Append results to summary data frame
  stat_results <- rbind(stat_results, data.frame(
    ScoreType = score_type,
    Group = desc_stats$Type,
    Mean = desc_stats$Score[, "mean"],
    SD = desc_stats$Score[, "sd"],
    Median = desc_stats$Score[, "median"],
    Pvalue = test_result$p.value
  ))
}

# Add significance annotations based on p-values
stat_results$Significance <- ifelse(stat_results$Pvalue < 0.001, "***",
                                    ifelse(stat_results$Pvalue < 0.01, "**",
                                           ifelse(stat_results$Pvalue < 0.05, "*", "ns")))

# Save statistical results to tab-delimited file
write.table(stat_results, file = "statistical_analysis_results.txt", sep = "\t",
            quote = FALSE, row.names = FALSE, col.names = TRUE)

# Generate violin plot with statistical comparisons
```

```
p <- ggviolin(data, x = "scoreType", y = "Score", fill = "Type",
              xlab = "",    # Remove x-axis label (customize as needed)
              ylab = "TME score",    # Y-axis label
              legend.title = gene,    # Legend title using gene name
              add = "boxplot", add.params = list(color = "white"),    # Overlay boxplot for median/quartiles
              palette = c("blue", "red"), width = 1)    # Set color palette
p <- p + rotate_x_text(45)    # Rotate x-axis labels to prevent overlap
# Add statistical significance markers
p1 <- p + stat_compare_means(aes(group = Type),
                             method = "wilcox.test",
                             symnum.args = list(cutpoints = c(0, 0.001, 0.01, 0.05, 1),
                                                symbols = c("***", "**", "*", " ")),
                             label = "p.signif")

# Save plot to PDF file
pdf(file = "vioplot.pdf", width = 6, height = 5)    # Set PDF dimensions
print(p1)    # Render plot
dev.off()    # Close PDF device

# Print summary to console
cat("Statistical analysis results saved to 'statistical_analysis_results.txt'\n")
cat("\nStatistical results summary:\n")
print(stat_results)
```
